# Multi-Omics Analysis of the Effect of cAMP on Actinorhodin Production in *Streptomyces coelicolor*

**DOI:** 10.3389/fbioe.2020.595552

**Published:** 2020-11-05

**Authors:** Katsuaki Nitta, Francesco Del Carratore, Rainer Breitling, Eriko Takano, Sastia P. Putri, Eiichiro Fukusaki

**Affiliations:** ^1^Department of Biotechnology, Graduate School of Engineering, Osaka University, Osaka, Japan; ^2^Department of Chemistry, Manchester Synthetic Biology Research Centre SYNBIOCHEM, Manchester Institute of Biotechnology, The University of Manchester, Manchester, United Kingdom

**Keywords:** *Streptomyces coelicolor*, metabolomics, transcriptomics, cAMP, secondary metabolites, actinorhodin, 7-methylguanine

## Abstract

Cyclic adenosine monophosphate (cAMP) has been known to play an important role in regulating morphological development and antibiotic production in *Streptomyces coelicolor*. However, the functional connection between cAMP levels and antibiotic production and the mechanism by which cAMP regulates antibiotic production remain unclear. In this study, metabolomics- and transcriptomics-based multi-omics analysis was applied to *S. coelicolor* strains that either produce the secondary metabolite actinorhodin (Act) or lack most secondary metabolite biosynthesis pathways including Act. Comparative multi-omics analysis of the two strains revealed that intracellular and extracellular cAMP abundance was strongly correlated with actinorhodin production. Notably, supplementation of cAMP improved cell growth and antibiotic production. Further multi-omics analysis of cAMP-supplemented *S. coelicolor* cultures showed an increase of guanine and the expression level of purine metabolism genes. Based on this phenomenon, supplementation with 7-methylguanine, a competitive inhibitor of reactions utilizing guanine, with or without additional cAMP supplementation, was performed. This experiment revealed that the reactions inhibited by 7-methylguanine are mediating the positive effect on growth and antibiotic production, which may occur downstream of cAMP supplementation.

## Introduction

*Streptomyces* species are soil-dwelling Gram-positive Actinobacteria and well-known important sources of bioactive secondary metabolites such as antibiotics (e.g., streptomycin; Davies et al., 1964), immunosuppressants (e.g., FK-506; Kino et al., 1987a,b), and anthelmintics (e.g., avermectin; Ikeda and Omura, 1997). Secondary metabolism is not necessary for bacterial growth and often occurs under nutrient limitation (Strauch et al., 1991), in specific medium condition (Lim et al., 2018), or in co-culture with other microbes (Onaka et al., 2011). The regulation of secondary metabolite production has therefore been a focus of research in natural product discovery and production (Liu et al., 2018). One way of improving secondary metabolite production is the addition of exogenous compounds such as cyclic adenosine monophosphate (cAMP) supplementation in *Streptomyces coelicolor* (Susstrunk et al., 1998), rare earth elements (e.g., scandium) in *S. coelicolor* (Tanaka et al., 2010), S-adenosyl-L-methionine (SAM) in *Streptomyces lividans* (Kim et al., 2003), and hormones with a butanolide skeleton in *Streptomyces virginiae* (Kim et al., 1989). However, in most cases, it is not fully understood by which mechanism such approaches led to the increased production of secondary metabolites.

With the recent rapid progress of analytical equipment and methods that contribute to the accumulation of more analytical data, new opportunities arise for studying the regulation of secondary metabolism. Transcriptome (Hwang et al., 2019), proteome (Millan-Oropeza et al., 2017), metabolome (Senges et al., 2018), and lipidome analyses (Zhang et al., 2020) or various combinations of these (Wang et al., 2017; Gosse et al., 2019) in *Streptomyces* have been applied to studies of secondary metabolism regulation.

In this study, we exploit these new technological opportunities to develop a more detailed picture of the regulatory circuitry surrounding the production of antibiotics in the model *Streptomyces* species *S. coelicolor*. Liquid chromatography tandem mass spectrometry (LC-MS/MS)–based metabolome analysis with wide metabolite coverage and RNAseq-based transcriptome analysis were employed, for comparative analyses of strains with differing antibiotic production phenotypes to identify the possible factors affecting secondary metabolite production and to provide a more detailed understanding of the regulation of secondary metabolites. This multi-omics analysis revealed that antibiotic production is correlated with cAMP levels, and cAMP supplementation to *S. coelicolor* culture led to improvements of cell growth and antibiotic production. Furthermore, multi-omics analysis of cAMP-supplemented cultures was performed to explore how the effects of cAMP are mediated through changes in the *S. coelicolor* metabolome and transcriptome.

These results provide valuable mechanistic insights into the effects of cAMP and their regulation, which in the future might be exploited for manipulating the cell growth and antibiotic production of *S. coelicolor.*

## Results and Discussion

### Phenotypes of *S. coelicolor* Strains for Omics-Based Comparative Analyses

**FIGURE 1:**
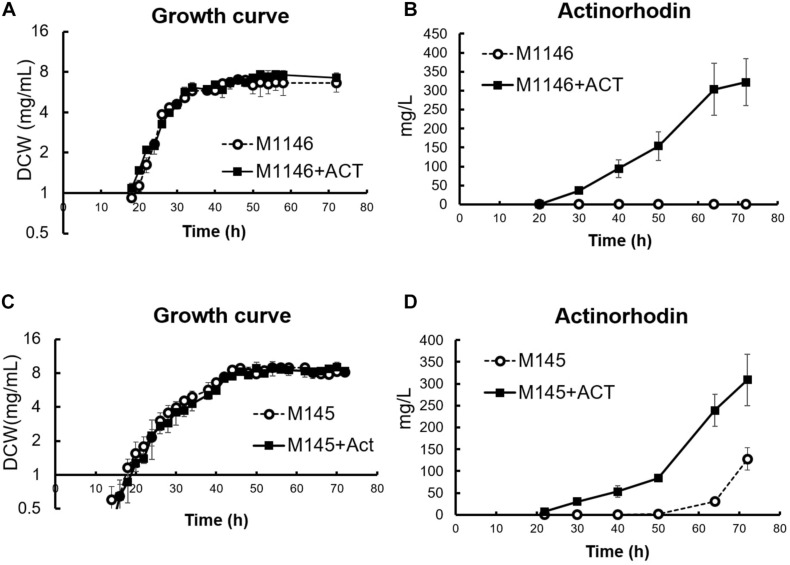
Cell growth and actinorhodin production in four *S. coelicolor* strains. **(A)** Growth curve (DCW: mg/mL) in M1146 (dot line chart) and M1146 + ACT (black line chart). Error bar means standard deviation from three replicates. **(B)** Extracellular actinorhodin production in M1146 (dot line chart) and M1146 + ACT (black line chart). Error bar means standard deviation from three replicates. **(C)** Growth curve (DCW: mg/mL) in M145 (dot line chart) and M145 + ACT (black line chart). Error bar means standard deviation from three replicates. **(D)** Extracellular actinorhodin production in M145 (dot line chart) and M145 + ACT (black line chart). Error bar means standard deviation from three replicates.

In a previous study, the M1146 strain was generated from strain M145 thorough step-by-step deletion of the four major native antibiotics biosynthetic gene clusters in *S. coelicolor*: *act* (actinorhodin, Act), *red* (undecylprodigiosin), *cpk* (coelimycin P1), and *cda* (calcium-dependent antibiotic). The original intention was to achieve higher antibiotic production ability by deleting these carbon-consuming pathways and increasing available carbon source for other (heterologously expressed) antibiotics producing pathways and to simplify the detection of the secondary produced that will be produced (Gomez-Escribano and Bibb, 2011). Here, we make use of this “clean” M1146 strain as a reference for an omics-based comparison against M1146 expressing a heterologous Act biosynthetic gene cluster by an integrated cosmid (M1146 + ACT) to identify the molecular consequences of the difference in antibiotic production phenotype. Act was chosen as the target compound for this study, as its intense blue coloration allows easy detection and quantitation of metabolite production. Introduction of the Act biosynthetic genes into M1146 did not affect growth (Figure 1A), but it caused Act production as expected (Figure 1B). Glucose and phosphate consumption did not show any statistically significant difference, but the M1146 + ACT strain showed slightly higher glutamate consumption, presumably because glutamate was used as a nitrogen source for Act production in this nutrient condition (Supplementary Figure 1A). We also analyzed the effect of amplifying Act production in the M145 strain (parent of M1146). An additional Act biosynthetic genes cluster was introduced into M145, as was done in M1146. M145 showed reproducible antibiotic production as in a previous study (Nieselt et al., 2010), and while growth was not affected (Figure 1C), M145 with the additional Act biosynthesis gene cluster (M145 + ACT) showed earlier Act production and a 2.41-fold higher Act production than M145 (Figure 1D). Contrary to the analogous case of M1146/M1146 + ACT, there was no significant difference in glutamate consumption between M145 and M145 + ACT (Supplementary Figure 1B), probably because in this case both strains produce Act and utilize glutamate as a nitrogen source for this purpose. However, phosphate was consumed more slowly in M145 + ACT compared to M145. Interestingly, the production of undecylprodigiosin (RED) also increased in M145 + ACT, whereas coelimycin production was not observed (Supplementary Figure 1C). All the strains used in this study are summarized in Supplementary Table 1.

### Metabolome Analysis of *S. coelicolor* Strains: Correlation of cAMP Levels With Actinorhodin Production

**FIGURE 2:**
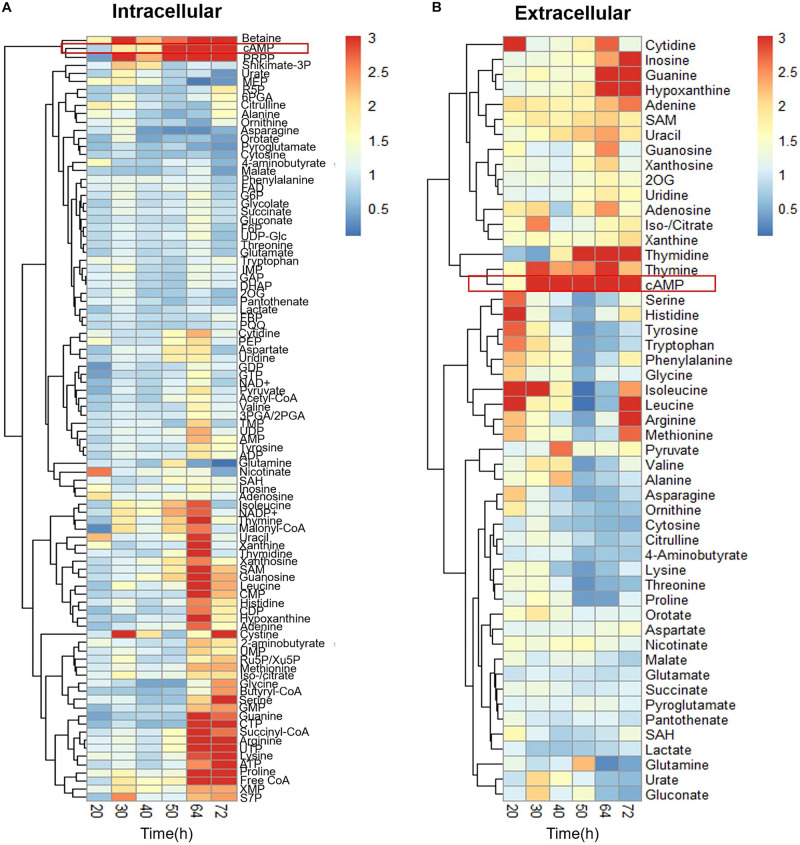
Time-course metabolome analysis of M1146 and M1146 + ACT. **(A)** Intracellular metabolite levels shown by fold change value using heatmap analysis. Comparison between M1146 and M1146 + ACT. Average values from three replicates were used to calculate the fold change value. **(B)** Extracellular metabolite levels shown by fold change value using heatmap analysis. Comparison between M1146 and M1146 + ACT. Average values from three replicates were used to calculate the fold change value. Metabolites circled by red box represent cAMP.

**FIGURE 3:**
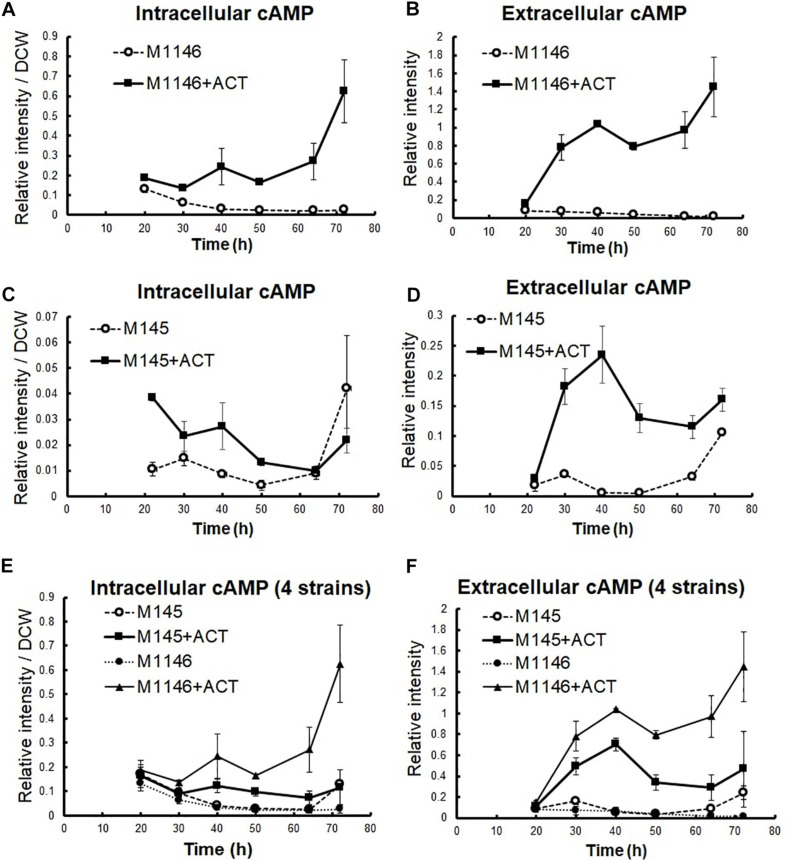
cAMP level measurement in four *S. coelicolor* strains. Error bar means standard deviation from three replicates. **(A)** Intracellular cAMP-level comparison between M1146 (dot line chart) and M1146 + ACT (black line chart). **(B)** Extracellular cAMP-level comparison between M1146 (dot line chart) and M1146 + ACT (black line chart). **(C)** Intracellular cAMP-level comparison between M145 (dot line chart) and M145 + ACT (black line chart). **(D)** Extracellular cAMP-level comparison between M145 (dot line chart) and M145 + ACT (black line chart). **(E)** Intracellular cAMP-level comparison between four strains: M145 (dot line chart with blank circle), M145 + ACT (black line chart with square), M1146 (dot line chart with black circle), and M1146 + ACT (black line chart with triangle). **(F)** Extracellular cAMP-level comparison between all four strains, in an independent replication experiment: M145 (dot line chart with blank circle), M145 + ACT (black line chart with square), M1146 (dot line chart with black circle), and M1146 + ACT (black line chart with triangle).

To understand the difference in metabolism with and without Act production, extracellular, and intracellular metabolome profiles of M1146 and M1146 + ACT were determined (Figure 2). Among the 99 metabolites analyzed (Supplementary Table 2), the major difference in both intracellular and extracellular metabolites between the two strains was that in cAMP levels (Figure 2). These were substantially higher in the M1146 + ACT compared to M1146 (Figures 3A,B). To support this observation, the intracellular and extracellular cAMP levels were also measured in M145 and M145 + ACT (Supplementary Figure 2) and showed higher cAMP levels in M145 + ACT compared to M145, as was observed in M1146 with and without ACT (Figures 3C,D). Comparing all four strains, cAMP production was strongly correlated with antibiotic production (e.g., production of Act and coelimycin P1; Figures 3E,F; and Supplementary Figure 1C).

### Transcriptome Analysis of *S. coelicolor* Strains

To further support the results of the targeted metabolome analysis, fine-grained transcriptome analysis was conducted on M1146 and M1146 + ACT at 10 time points during growth (Figure 1A).

**FIGURE 4:**
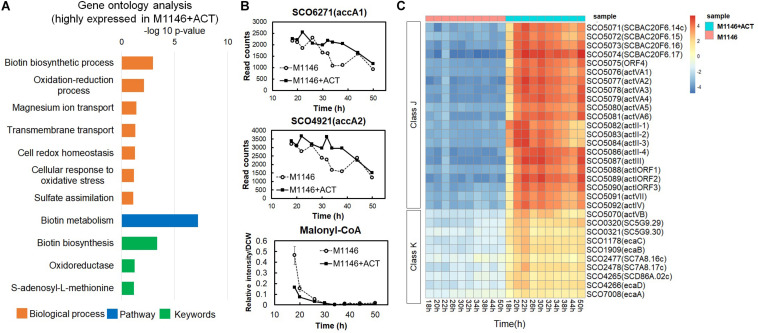
Transcriptome analysis of M1146 and M1146 + ACT. **(A)** Gene set enrichment analysis by means of highly expressed genes from identified differentially expressed genes (DEGs) in time-course transcriptome analysis of M1146 and M1146 + ACT. Biological process, pathway, and keywords were used to define the gene sets. **(B)** Gene expression level comparison of two acetyl-CoA carboxylases and metabolite-level comparison of malonyl-CoA between M1146 (dot line chart) and M1146 + ACT (black line chart). The error bar in malonyl-CoA–levels line chart means standard deviation from three replicates. **(C)** K-means clustering of transcriptome analysis of M1146 and M1146 + ACT (*k* = 20). Gene expression trends in the focus classes J and K are shown here. K-means clustering results using all genes are shown in Supplementary Figure 2.

Differentially expressed genes (DEGs) between M1146 and M1146 + ACT were identified in order to further evaluate the effects of Act production on gene expression levels. The false discovery rate (FDR) was controlled at 5%, and only genes with an absolute log2-fold change (FC) greater than 0.5 were included in the analysis. As expected, the genes of the Act biosynthetic gene cluster were only expressed in M1146 + ACT (at very high levels), whereas no expression was detected in M1146, which lacks this gene cluster (Supplementary Table 3). As this observation is trivial, these enzyme-coding genes were excluded from the subsequent analysis of differential expression. In total, 100 genes were identified as overexpressed in M1146 + ACT, whereas 15 genes were identified as less abundantly expressed in M1146 + ACT based on the DEG criteria described above (Supplementary Table 4). Based on the DEGs, Gene Set Enrichment Analysis (GSEA) was performed, using biological process, pathway, and keywords to define the gene sets (Figure 4A and Supplementary Table 5). The major overexpressed gene sets in M1146 + ACT were associated with biotin biosynthesis and oxidation/reduction processes (Figure 4A).

High expression of biotin biosynthetic genes is perhaps not surprising, as biotin is a necessary cofactor for malonyl-CoA synthesis, which is the essential building block for biosynthesis of actinorhodin (a polyketide; Katz and Donadio, 1993). This observation also matches the high levels of expression of the two acetyl-CoA carboxylase–encoding genes, SCO6271 (*accA1*) and SCO4921 (*accA2*) in M1146 + ACT (Figure 4B). When malonyl-CoA levels were analyzed by LC-MS/MS, malonyl-CoA decreased with time, and the malonyl-CoA level was lower in M1146 + ACT at 20 h (Figure 4B). Thus, malonyl-CoA was consumed earlier in the M1146 + ACT strain, which is presumably due to the malonyl-CoA being intensely utilized for Act biosynthesis. The difference in expression levels of genes encoding enzymes responsible for cAMP biosynthesis, adenylate cyclase (SCO4928), and cAMP degradation, phosphodiesterase (SCO6075), was not statistically significant (Supplementary Figure 3). The expression level of both genes is low and was only slightly increased in M1146 + ACT and showed a very similar trend, where a second peak of increased gene expression was seen at 30 h, which coincides with the cells entering the stationary phase. This in itself cannot explain the observed difference in cAMP production. The discrepancy in the expression of the genes encoding cAMP synthesis and degradation and cAMP levels is intriguing and will need further study to fully understand this phenomenon.

To reveal trends in the gene expression time courses, the transcriptome data were subjected to *k*-means clustering (*k* = 20; Figure 4C, Supplementary Figure 4, and Supplementary Excel File 1). All genes were clustered into 20 classes, and classes J and K genes were more highly expressed in M1146 + ACT at all sampling points. Class J consisted of 22 genes encoding enzymes involved in Act biosynthesis, whereas class K has 10 genes, most of which were reported as related to the *soxR* regulon (Table 1; Dela Cruz et al., 2010; Naseer et al., 2014). The *SoxR* regulon was reported to be upregulated by oxidative stress caused by high levels of Act production in previous studies (Shin et al., 2011; Mak and Nodwell, 2017), which would be in agreement with our results.

**TABLE 1:** List of selected class K genes in heatmap analysis of time-course RNAseq of M1146 and M1146 + ACT.

Locus tag	Product	Note	Reference
SCO1178	NAD-dependent epimerase/dehydratase	SoxR regulon	Naseer et al., 2014
SCO1909	Monooxygenase	SoxR regulon	
SCO4266	Oxidoreductase	SoxR regulon	
SCO7008	ABC transporter ATP- binding protein	SoxR regulon	
SCO2478	Flavoprotein reductase	SoxR regulon	
SCO0320	Quinone oxidoreductase	Potential SoxR regulon	
SCO0321	Carboxylesterase	Potential SoxR regulon	

### cAMP Supplementation to *S. coelicolor* Increased Cell Growth and Secondary Metabolite Production

**FIGURE 5:**
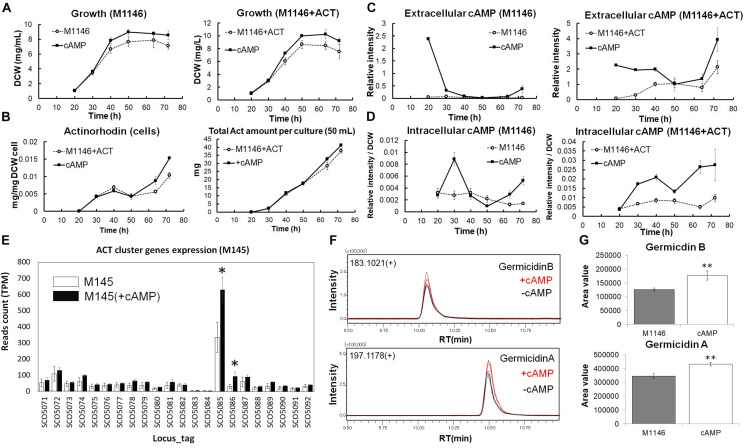
Analysis of cAMP-supplemented to M1146 and M1146 + ACT. **(A)** Cell growth (mg/mL) comparison between the control sample (dot line chart) and cAMP-supplemented sample (black line chart) in M1146 and M1146 + ACT. cAMP was supplemented at the time of the first sampling (20 h). The left panel shows the growth comparison between M1146- and cAMP-supplemented M1146, whereas the right panel shows the growth comparison between M1146 + ACT- and cAMP-supplemented M1146 + ACT. Error bars indicate the standard deviation from three replicates. **(B)** Actinorhodin production (mg/L) comparison between the control sample (dot line chart) and cAMP-supplemented sample (black line chart) in M1146 + ACT during the growth. The left panel shows intracellular actinorhodin production comparison between M1146 + ACT- and cAMP-supplemented M1146 + ACT, whereas the right panel shows total actinorhodin (inside and outside the cell, per 50-mL culture) comparison between M1146 + ACT- and cAMP-supplemented M1146 + ACT. Error bars indicate the standard deviation from three replicates. **(C)** Extracellular cAMP-level comparison between the control sample (dot line chart) and cAMP-supplemented sample (black line chart) during the growth. The left panel shows extracellular cAMP-level comparison between M1146- and cAMP-supplemented M1146, whereas the right panel shows extracellular cAMP-level comparison between M1146 + ACT- and cAMP-supplemented M1146 + ACT. Error bars indicate the standard deviation from three replicates. **(D)** Intracellular cAMP-level comparison between the control sample (dot line chart) and cAMP-supplemented sample (black line chart) during the growth. The left panel shows intracellular cAMP-level comparison between M1146- and cAMP-supplemented M1146, whereas the right panel shows intracellular cAMP-level comparison between M1146 + ACT- and cAMP-supplemented M1146 + ACT. Error bars indicate the standard deviation from three replicates. **(E)** Gene expression level comparison between non–cAMP-supplemented M145 (white bar graph) and cAMP-supplemented M145 (black bar graph). Read count normalized by TPM (transcripts per million) was used, and error bar means standard deviation from three replicates. Gene names are listed in Supplementary Table 7. Asterisk means significant difference (*p* < 0.01). **(F)** Chromatograms of germicidin B and germicidin A from ethyl acetate extracts from M1146 culture with (red line) and without (black line) the addition of cAMP. **(G)** Area value comparison between non–cAMP-supplemented and cAMP-supplemented M1146. Error bar means standard deviation from three replicates. Double asterisk means significant difference (*p* < 0.01).

To explore whether the observed correlation of cAMP levels with antibiotic production was the result of a causal connection between the two phenomena, we performed a cAMP supplementation experiment (Figure 5). cAMP has been previously reported as an inducer of Act production (Susstrunk et al., 1998), and the deletion of the *cya* (SCO4928) encoding the adenylate cyclase abolished antibiotic production (Susstrunk et al., 1998; Kang et al., 1999), whereas the cAMP receptor protein CRP encoded by gene SCO3571 was shown to be important for morphological development, and ChIP-chip experiments showed secondary metabolism gene clusters including Act contained Crp-associated sites (Derouaux et al., 2004; Gao et al., 2012). Before conducting the cAMP supplementation, cAMP stability in cell-free medium and cAMP uptake by M1146 at 1 h and 2 h after cAMP supplementation was confirmed (Supplementary Figures 5A,B). Interestingly, when M1146 and M1146 + ACT were supplemented with 10 μM cAMP at the mid–log phase (20 h), cell growth increased; the cell masses after supplementation were 1.20- and 1.22-fold higher in M1146 and M1146 + ACT, respectively (Figure 5A). Moreover, intracellular Act production increased in M1146 + ACT (Figure 5B and Supplementary Figure 6A) starting after 50 h, and at 72 h, the total Act production increase was 1.1-fold on average; this is a very minor increase, but statistically significant (*p* < 0.05; Figure 5B). Interestingly, extracellular supplemented cAMP rapidly decreased in M1146, but showed a slower decrease in M1146 + ACT (Figures 5C,D). After 50 h, a renewed increase of extracellular cAMP was observed with or without the addition of cAMP (Figure 5C), but the extracellular cAMP accumulation was higher in the cAMP-supplemented M1146 + ACT culture. The intracellular cAMP showed an increase in both strains with addition of external cAMP (Figure 5D).

To evaluate the effects of cAMP on gene expression levels, RNAseq-based transcriptome analysis of cAMP-supplemented cultures of M145 was performed, and DEGs were identified (Supplementary Table 6) based on DEG criteria. cAMP 3 μM was added to M145 cultures after 48 h of the growth and samples were taken at 50 h. Interestingly, cAMP addition increased expression of two genes from the Act biosynthetic gene cluster; SCO5086 (ketoacyl reductase) increased most dramatically by 3.05-fold on average, and SCO5085 (Act biosynthesis pathway-specific activator actII-ORF4) increased by 1.93-fold on average (Figure 5E). Act biosynthesis is known to be tightly controlled by *actII*-ORF4 (Gramajo et al., 1993), and Act production starts after the increased expression of this pathway-specific activator (Gramajo et al., 1993). It is also reported that increasing the transcription of *actII*-ORF4 results in the overproduction of Act (Gramajo et al., 1993). The increase of *actII*-ORF4 in our results is in line with these previous reports and provides a possible direct link between cAMP supplementation and Act production at the level of gene expression.

Interestingly, following the rapid disappearance of supplemented cAMP from the culture medium, a minor accumulation of extracellular cAMP was observed in M1146 at 72 h (Figure 5C). As we had previously observed that antibiotic production was correlated with extracellular cAMP accumulation, we hypothesized that this cAMP accumulation might be related to the production of other secondary metabolites in M1146—while this “clean” strain lacks the capacity to produce the four major antibiotics, 18 other potential biosynthetic gene clusters are known to be still present in the genome (Bentley et al., 2002). To understand if the effect of cAMP addition on the increase of Act production is accompanied by consequences for the production of other secondary metabolites, ethyl acetate extracts of cultures of M1146 and M1146 supplemented with cAMP were measured by LC-QTOF (Quadrupole Time-OF-Flight) MS. As shown in previous studies, antibiotic production heavily depends on the utilized medium (Cihak et al., 2017; Lim et al., 2018). Based on previous data on *S. coelicolor* secondary metabolites, candidate peaks were explored, and two secondary metabolites, namely, germicidin A (theoretical mass: 197.11777, observed mass: 197.118462, mass error: 4.0 ppm) and germicidin B (theoretical mass: 183.10212, observed mass: 183.102920, and mass error: 3.5 ppm), were detected in the extracts (Figure 5F). The annotation was confirmed by MS/MS analysis of each peak (Supplementary Figures 6B,C), and both MS/MS spectra matched the MS/MS spectra from a previous study (Cihak et al., 2017). Based on the peak areas, the production of the two germicidins significantly increased (*p* < 0.01) in the cAMP-supplemented culture (Figure 5G).

### Metabolomics and Transcriptomics Analysis of cAMP-Supplemented *S. coelicolor* Cultures

**FIGURE 6:**
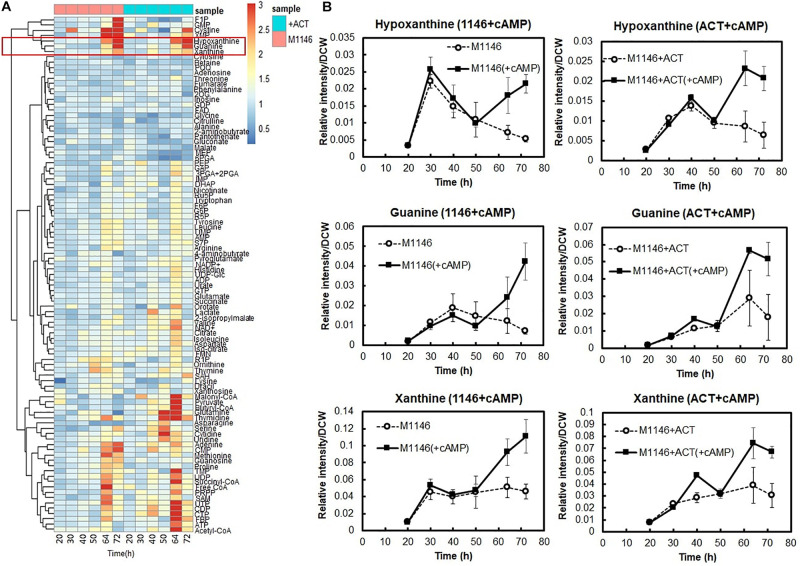
Metabolome analysis of cAMP-supplemented M1146 and M1146 + ACT. **(A)** Intracellular metabolome analysis of cAMP-supplemented M1146 and M1146 + ACT during growth. The metabolite amount difference is shown by fold change of area value (cAMP supplemented/non–cAMP supplemented) in heatmap clustering. Average values from three replicates were used to calculate the fold change values. This heatmap consists of data of cAMP-supplemented of M1146 (pink, left six panels) and M1146 + ACT (blue, right six panels). The metabolites are listed in Supplementary Table 2. **(B)** Intracellular selected metabolite-level (hypoxanthine, guanine, and xanthine) comparison between non–cAMP-supplemented and cAMP-supplemented culture. The left figure shows the comparison between M1146 (dot line, circle) and cAMP-supplemented M1146 (black line, square), whereas the right figure shows the comparison between M1146 + ACT (dot line, circle), and cAMP-supplemented M1146 + ACT (black line, square). Error bar means standard deviation from three replicates.

In order to understand how cAMP improves secondary metabolite production and cell growth, comparative metabolome analysis of M1146 and M1146 + ACT with and without cAMP supplementation was performed (Figure 6A and Supplementary Figure 7). We measured a total of 98 metabolites. The analysis here focuses on metabolites, which consistently increased in M1146 and M1146 + ACT when cAMP was supplemented.

Interestingly, levels of purine bases, such as hypoxanthine guanine and xanthine, substantially increased at 64 h and 72 h when cAMP was supplemented (Figures 6A,B). Therefore, we hypothesized that increased guanine, xanthine, and hypoxanthine levels and the purine base–utilizing reactions might be a key factor mediating increased cell growth and antibiotic production improvement following cAMP supplementation. This would be in line with earlier observations of a link between purine metabolism and antibiotic production, through their involvement in the synthesis of guanosine tetraphosphates and pentaphosphates (p)ppGpp and the second messenger cyclic-di-GMP (c-di-GMP; Sivapragasam and Grove, 2019).

**FIGURE 7:**
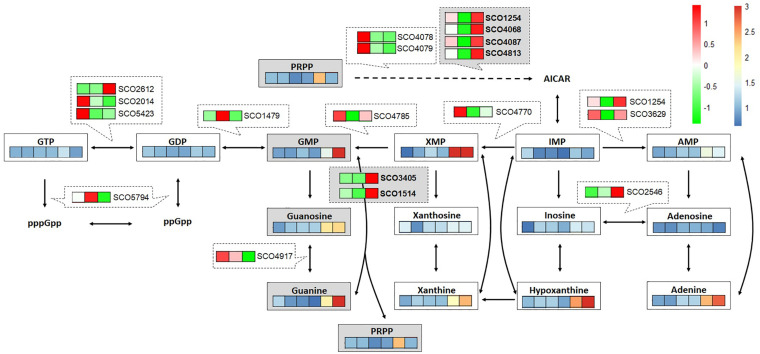
Multi-omics analysis of cAMP-supplemented M1146 mapped to purine metabolism. Time course (20, 30, 40, 50, 64, and 72 h: from left to right) of metabolite-level fold change between non–cAMP-supplemented M1146- and cAMP-supplemented M1146 is shown on a color scale from blue to red. Average values from three replicates were used to calculate the fold change. Time-course gene expression (0, 1, and 2 h: from left to right) changes in response to cAMP supplementation are shown on a color scale from green to red. The average value of read counts was normalized by autoscaling to see the difference well.

To support the metabolome analysis result and to understand the phenomenon caused by cAMP supplementation at the level of gene expression, transcriptome analysis of cAMP was performed. To distinguish between the direct effects of cAMP supplementation and indirect effects of Act production, we performed transcriptome analysis on M1146 with addition of cAMP. Based on the same differential expression criteria as described above, DEGs were identified between time points 0, 1, and 2 h (Supplementary Figure 8 and Supplementary Table 8) to identify genes that showed different expression in response to cAMP supplementation. Here, gene expression related to purine metabolism was mapped to the metabolic pathway (Figure 7). The results showed increased expression levels of genes, encoding enzymes involved in the synthesis of 5-aminoimidazole-4-carboxamide ribonucleotide (a shared precursor of purines) in response to cAMP supplementation: SCO1254 (2SCG1.29), SCO4068 (*purD*), SCO4087 (*purM*), and SCO4813 (*purN*). This transcriptome trend is consistent with purine bases (guanine, xanthine, hypoxanthine, and adenine) increasing at the metabolome level. In addition, expression levels of genes encoding salvage pathway enzymes [SCO1514, *apt*, and SCO3405, *hprT* (FDR = 0.077 and log2FC = 0.64)] increased. These phenomena at transcriptome level are consistent with the metabolome-based observation that guanine and GMP levels increase as cAMP is supplemented (Figure 7). Therefore, among the candidate key metabolites (guanine, xanthine, and hypoxanthine) identified to be increasing in the metabolome analysis of cAMP-supplemented culture, it was suggested that guanine or a guanine-related reaction may be mediating the effect of cAMP on cell growth and antibiotic production.

**FIGURE 8:**
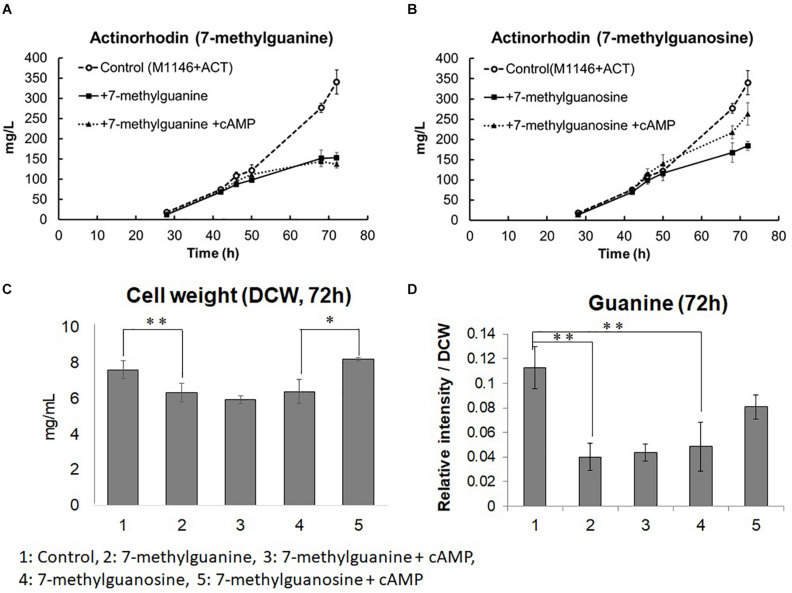
Actinorhodin production inhibition by 7-methylguanine and 7-methylguanosine in M1146 + ACT and restoration by addition of cAMP. **(A)** Actinorhodin production in control (M1146 + ACT; semi dotted line, circle), 7-methylguanine (20 μM)-supplemented (black line, square), and 7-methylguanine (20 μM)– and cAMP (10 μM)–supplemented (dotted line, triangle) cultures. Error bar means standard deviation from three replicates. Circle means control sample, square means 7-methylguanine–supplemented, and triangle means 7-methylguanine– and cAMP-supplemented culture. **(B)** Actinorhodin production in control (M1146 + ACT; semi dotted line, circle), 7-methylguanosine (20 μM)-supplemented (black line, square), and 7-methylguanosine (20 μM)– and cAMP (10 μM)–supplemented (dotted line, triangle) cultures. Error bar means standard deviation from three replicates. **(C)** Cell weight (DCW: mg/mL) comparison at 72 h. Error bar means standard deviation from three replicates. Asterisk means significant difference (**p < 0.01, *p < 0.05). **(D)** Guanine amount comparison at 72 h. Error bar means standard deviation from three replicates. Asterisk means significant difference (**p < 0.01).

To test this hypothesis, we supplemented M1146 + ACT at the mid–log phase with 20 μM 7-methylguanine, which is an analog of guanine that inhibits guanine utilizing reactions by competitive inhibition (Goodenough-Lashua and Garcia, 2003; Fernandez et al., 2010). In addition, 20 μM 7-methylguanosine, which is a possible competitive inhibitor of guanosine utilizing reactions (Pathak et al., 2005), was also supplemented independently at the mid–log phase of M1146 + ACT, to test the idea that guanosine instead of guanine might be the active metabolite in this regulatory system (Figures 8A,B). Interestingly, both 7-methylguanine, and 7-methylguanine supplementation impaired Act production by 2.22- and 1.85-fold, respectively. In addition, both 7-methylguanine and 7-methylguanosine supplementation impaired guanine levels by 2.81- and 2.31-fold, respectively (Figure 8D). In order to identify whether this impairment occurs downstream or upstream of the positive effect of cAMP supplementation, cAMP was also supplemented to the two inhibitor-treated cultures. cAMP supplementation recovered about 50% of Act production only in the 7-methylguanosine–supplemented culture (Figures 8A,B). Cell growth was monitored at 72 h after cAMP was supplemented to the 7-methylguanine– or 7-methylguanosine–treated M1146 + ACT. 7-methylguanine–treated cultures after cAMP supplementation still showed a decreased amount of cells (*p* < 0.01), whereas cAMP supplementation of 7-methylguanosine–treated cultures restored the cell amount to untreated levels (*p* < 0.05; Figure 8C). Guanine levels in the cells in inhibitor- and cAMP-supplemented cultures were also monitored and compared at 72 h; here, the addition of cAMP did not restore the guanine level in 7-methylguanine–supplemented culture, whereas addition of cAMP restored the guanine level in 7-methylguanosine–supplemented cultures (by 1.66-fold, statistically not significant; Figure 8D). This is in partial agreement with our original hypothesis that an increase in guanine is necessary for cell growth and antibiotic production improvement by cAMP supplementation. In summary, the addition of cAMP to 7-methylguanine–supplemented culture did not restore Act production and cell growth, whereas the addition of cAMP to 7-methylguanosine–supplemented culture restored the impaired Act production and cell growth. Therefore, the negative effect of 7-methylguanine supplementation occurs upstream of the effects of cAMP supplementation, and we suggest that the reaction(s) inhibited by 7-methylguanine are mediating the positive effect on growth and antibiotic production in *S. coelicolor*.

## Conclusion

In this study, a metabolomics- and transcriptomics-based approach was applied to elucidate the effect of Act production in *S. coelicolor*. The analysis showed that Act production was highly correlated with an increase in extracellular cAMP levels, and cAMP supplementation was found to increase antibiotic production and cell growth. Further multi-omics analysis of cAMP-supplemented cultures showed that guanine levels increased in response to cAMP supplementation, and inhibition of guanine utilizing reactions by the analog 7-methylguanine confirmed that some reaction or reactions inhibited by 7-methylguanine mediate the positive effect of cAMP supplementation on growth and antibiotic production.

## Materials and Methods

### Bacterial Strain, Medium, Growth Condition

Bacterial strains were grown in 50 mL liquid minimum nutrition medium with same composition as previous study (Nieselt et al., 2010) in well-siliconized 250-mL flasks containing stainless-steel springs. For inoculation, 1 × 10^9^ colony-forming units of spores were inoculated to the 50 mL medium. Incubation speed and temperature were set to 220 rpm and 30°C, respectively. To measure growth, cells were collected in 2 mL Eppendorf tube and centrifuged to discard supernatant and washed well by dH_2_O and lyophilized by freeze drying for measurement of dry cell weight (DCW).

### Antibiotic Production Quantification

Procedure to quantify extracellular actinorhodin was based on previous study (Nieselt et al., 2010). Cells for intracellular actinorhodin quantification were sampled by fast filtration with 5.0-μm pore size nylon membrane filter and subjected to vacuum fast filtration, and the cells were quenched in 15-mL Falcon tube by liquid nitrogen and kept at −80°C until extraction. The cells were extracted by 4 mL of mix-solvent solution [methanol, chloroform, and water 5:2:2 (vol/vol/vol)] with three cycles of freeze and thaw cycle (freezing at −80°C and thawing at −30°C). The extracts were centrifuged at 10,000 rpm for 10 min, and 2 mL supernatant was transferred to new 15-mL Falcon tube, and 1 mL ultrapure water was added to the supernatant and vortexed well. The mixture was separated to two layers by centrifugation at 10,000 rpm for 10 min, and the upper layer was used for intracellular actinorhodin quantification. The colored extracts were diluted with the same amount of 1 M NaOH solution, and OD608 was measured by photometer. Here, a culture with an already known actinorhodin concentration was extracted exactly the same way as was done for intracellular actinorhodin extraction, and the OD608 was measured. The value was used to quantify the unknown amount of intracellular actinorhodin.

### Culture Sampling for Metabolome Analysis

Based on the growth curve of each strain, more than 10 mg DCW cultures were sampled by nylon membrane filter with 5.0-μm pore size and 47-mm diameter and subjected to vacuum fast filtration. Cells on the filter were washed using twice amount of 3.5% (vol/vol) NaHCO_3_ solution to wash the medium component away. The filter with cells was immediately quenched in 15-mL Falcon tubes by liquid nitrogen and kept at −80°C until extraction. One-milliliter culture was sampled for extracellular metabolome analysis and medium components analysis and Act quantification. The culture was centrifuged at 10,000 rpm for 10 min, and supernatant was kept at −80°C until extracellular metabolites extraction and medium components consumption analysis. For time-course metabolome analysis and medium components analysis, 20, 30, 40, 50, 64, and 72 h were selected for sampling points based on the growth curve. In the case of comparing the metabolome analysis data with transcriptome analysis data, 18, 20, 22, 26, 30, 32, 34, 38, 44, and 50 h were selected for extracellular metabolome analysis, whereas less time points, 18, 22, 26, 30, 34, 38, 44, and 50 h, were selected for intracellular metabolome analysis due to lack of sampling volume.

### Medium Components (Glucose, Glutamate, and Phosphate) Consumption Quantification

Medium components, glucose, glutamate, and phosphate, were measured by commercially available quantification kits. F-kit-D-glucose (J. K. International), L-glutamate assay kit Yamasa NEO (Yamasa), and PiBlue phosphate assay kit (BioAssay Systems) were used for quantification of glucose, glutamate and phosphate in medium, respectively, by following manufacturing guides.

### Extraction of Cells and Medium for Metabolome Analysis

For intracellular metabolome analysis, 4 mL of mix solvent [methanol, chloroform, and water ratio 5:2:2 (vol/vol/vol)] with 50 μg/L (+) 10-camphorsulfonic acid as an internal standard was added to frozen cells in 15-mL Falcon tubes and extracted with three cycles of freeze and thawing cycles (freezing at –80°C, thawing at –30°C, vortexing for 10 s, and sonication for 10 s). The extracted cells were centrifuged at 10,000 rpm, 4°C for 10 min, and the supernatant was used for the following extraction procedures. To the supernatant (2 mL), 1 mL ultrapure water was added and vortexed for 5 s and separated to two layers by centrifugation (10,000 rpm, 4°C) for 10 min. The upper polar phase was filtered by 0.2 μm PTFE hydrophilic membrane filter, and the 2 mL extract solution was applied to centrifugal concentration by spin dryer for 2 h, and the samples were lyophilized overnight. The lyophilized sample was kept at −80°C until analysis. The lyophilized sample was reconstituted with 400 μL ultrapure water when analyzed. The extracted cells were washed by ultrapure water and subjected to freeze drying to calculate DCW used for intracellular metabolome analysis and normalize metabolite abundance by the cell amount.

For extracellular metabolome analysis, 50 μL culture supernatant was added to 1.8 mL mix solvent with the same composition for intracellular metabolites extraction. The mixture was vortexed for 5 s and kept at -30°C, and the extracts were centrifuged at 10,000 rpm and 4°C for 10 min. The supernatant (1.3 mL) was mixed with 0.65 mL ultrapure water and vortexed for 5 s and separated to two layers by centrifugation at 10,000 rpm and 4°C for 10 min, and supernatant was filtered by 0.2 μm PTFE hydrophilic membrane filter. Filtered sample (1 mL) was applied to centrifugal concentration by spin dryer for 1 h, and the sample was lyophilized overnight, and lyophilized sample was kept at -80°C until analysis. The lyophilized sample was reconstituted with 200 μL ultrapure water when analyzed. Intracellular and extracellular reconstituted sample solution (40 μL) was transferred to LC vial for following LC-MS/MS analysis.

### LC-MS/MS Analysis for Intracellular and Extracellular Metabolome Analysis

In this study, two kinds of LC-MS/MS platform were employed for achieving a wider range of metabolite coverage. One is ion-pair LC-MS/MS with negative ionization mode, which is previously described (Nitta et al., 2017). Acquired data from this analysis were analyzed by Lab solution (Shimadzu).

For sugar phosphate isomer separation, different gradient (see below) was employed for better chromatographic separation, whereas other parameters were set to be the same. The gradient is as follows: Percentage of mobile phase B was held at 0% for 1 min and raised to 50% in 30 min and raised to 100% in a minute. After holding at 100% for 1 min, the percentage was decreased to 0% in a minute and held at 0% for 6 min for column equilibration for the next analysis. All metabolites’ abundance was normalized by area value of internal standard [(+) 10-camphorsulfonic acid] and the DCW used for metabolite extraction.

The other platform is LC-MS/MS in positive ionization mode with ESI (electrospray ionization) mode. Nexera X2 UHPLC coupled to LCMS 8050 (Shimadzu) with Discovery HS F5-3 (3 μm, 150 × 2.1 mm) column was used. Formic acid 0.1% (vol/vol) in ultrapure water was used for mobile phase A, whereas acetonitrile was used for mobile phase B.

Column oven temperature was set to 40°C, and injection volume was set to 3 μL. The chromatographic separation was conducted by gradient mode as follows. The total flow rate was set to 0.2 mL/min, and percentage of mobile phase B was held at 0% for 5 min and raised with a gradient for 4%/min until 40% and held for 10 min and then raised with a gradient 60%/min until 100%. After holding at 100% for 2.5 min, the percentage was decreased to 0% in a minute and held at 0% for 6 min for column equilibration for the next analysis. Mass spectrometer parameters were set to as follows: DL (desolvent line) temperature was set to 250°C; heating block temperature was set to 400°C; nebulizer gas flow was set to 3 L/min; drying gas flow rate was set to 10 L/min; heating gas flow rate was set to 10 L/min; and interface temperature was set to 400°C. All analyses were performed by MRM mode. Acquired data from this analysis were analyzed by Lab solution (Shimadzu). All metabolites’ abundance was normalized by area value of internal standard [(+) 10-camphorsulfonic acid]. Metabolome analysis data have been deposited to the Metabolights public repository under accession numbers MTBLS1984 and MTBLS2025.

### Culture Sampling for RNAseq Analysis

Before sampling, all solutions used for RNA extraction was autoclaved two times to avoid RNase contamination. Based on the growth curve, more than 2.0 mg DCW cell cultures were sampled and mixed with twice the amount of RNA protection reagent (Qiagen), immediately. The cells were vortexed for 10 s, kept for 5 min at room temperature, and centrifuged at 10,000 rpm, 20°C for 10 min. Supernatant was eliminated as much as possible, and the cells were quenched by liquid nitrogen immediately and kept at −80°C until RNA isolation. For time-course RNAseq of M1146 and M1146 + ACT, the samples were collected at 18, 20, 22, 26, 30, 32, 34, 38, 44, and 50 h. For RNAseq of cAMP-supplemented M145, 3 μM cAMP was supplemented to M145 at 48 h, and the samples were collected at 50 h. For RNA-Seq of cAMP-supplemented M1146, 10 μM cAMP was supplemented at 20 h, and the samples were collected at 21 and 22 h.

### Cell Lysis, RNA Extraction, Purification, and Quality Check

Cells were resuspended by double-autoclaved empty tip, and 0.17 mL of 15 mg/mL lysozyme was added to the cells and incubated at 30°C for 10 min. The solutions including cells were transferred to tube containing lysing matrix E (MPbio medicals) and 0.6 mL RLT buffer (Qiagen) supplemented with β-mercaptoethanol (100:1, vol/vol) and vortexed for 5 s. Three pulses were applied to the tube by Fast Prep (6.5 m/s, 30 s), whereas the tubes were kept on ice between pulses for 30 s. The tubes were centrifuged at 10,000 rpm and 4°C for 1 min, and the lysate was recovered. After centrifuging heavy Phase Lock Gel (PLG) tube to pack the resin for 1 min, 0.65 mL recovered lysate was transferred to the heavy Phase Lock Gel (PLG) tube; 0.65 mL isoamyl alcohol and acid phenol mixture were added to the lysate in heavy PLG tube and mixed by inversion for 1 min. The heavy PLG tubes containing extracts were centrifuged for 5 min, and superior aqueous phase was recovered. RNA purification was performed with Direct-zol RNA MiniPrep Plus (Zymo research). After 0.6 mL ethanol was added to recovered 0.6 mL of aqueous phase and mixing by pipette, Zymo-spin IIICG column was assembled to collection tube, and 0.6 mL mixture solution was transferred to the assembled column-collection tube and centrifuged at 10,000 rpm for 30 s. Flow-through from the collection tube was discarded, and the remaining 0.6 mL of mixture of aqueous extract and ethanol was transferred to the assembled column-collection tube, consequently centrifuged at 10,000 rpm for 30 s. The flow-through in the collection tube was discarded again. RNA wash buffer completed with ethanol (0.4 mL) was added to the column and centrifuged at 10,000 rpm for 30 s, and the flow-through was discarded. To the column matrix, 80 μL of DNase (6 U/μL) was added and incubated at 30°C for 15 min. After incubation, RNA was washed for two cycles (adding 0.4 mL of Direct-zol RNA prewash to the column, centrifuging at 10,000 rpm for 30 s and discarding the flow through). To the column, 0.7 mL RNA wash buffer was added and centrifuged for 2 min to ensure complete removal of wash buffer. Consequently, the column was transferred to a new RNase-free tube; 50 μL of DNase/RNase-free water was added and incubated at room temperature for 1 min. The column with sample containing water was centrifuged at 10,000 rpm for 1 min, and the tube with the RNA sample was frozen by liquid nitrogen until the next step. RNA concentration was quantified by NanoDrop (ThermoFisher Scientific), and RNA quality based on RNA integrity number was evaluated by Bio-analyzer (Agilent Technologies).

### Ribosomal RNA Deletion, cDNA Library Construction, and Sequencing

All rRNA deletion, strand-specific cDNA library construction, and the sequencing experiment were outsourced to Vertis Co. (Germany) and Genewiz Japan (Japan) and performed by their in-house–developed methods. Time-course RNAseq analysis of M1146 and M1146 + ACT and RNAseq of cAMP-supplemented M145 was performed by Vertis (Germany), and cDNA pools were sequenced on an Illumina NextSeq 5000 system using 75-bp length with 1 × 10-M reading depth. Time-course RNAseq analysis of cAMP-supplemented M1146 was performed by Genewiz (Japan), and cDNA pools were sequenced by Illumina HiSeq X-ten system using 150-bp length with 2 × 10-M reading depth.

### RNASeq Data Analysis

Sequencing data were obtained from Vertis Co. and Genewiz Co. in FASTQ format. Reference genome data of *S. coelicolor* were downloaded from NCBI, and generation of reference genome library and read mapping to the reference genome library were performed by STAR version 2.7 (Dobin et al., 2013) on a Linux PC. Obtained SAM files were converted to BAM file by Samtools (Li et al., 2009). Defining gene model, reads counting, normalization, and differential gene expression analysis procedures were based in a previous study (Love et al., 2015). All data were transferred to R version 3.6.1, and BAM file list was generated by R package Rsamtools; reference gene model was generated by R package GenomicFeatures (Lawrence et al., 2013); sequencing reads were counted R package GenomicAlignments (Lawrence et al., 2013), and finally RLE normalization and differential gene expression analysis were performed by R package DESeq2 (Love et al., 2014). For defining differential gene expression, a threshold of FDR less than 5% and log2 FC greater than 0.5 was chosen. RNAseq analysis data have been deposited to the NCBI Gene Expression Omnibus public repository under accession numbers GSE155796, GSE158810, and GSE158811.

### GSEA

Gene Set Enrichment Analysis was performed using the DAVID Functional Annotation Bioinformatics Microarray Analysis version 6.8 (Huang da et al., 2009a,b). The GSEA was performed based on biological process, pathway, and keywords.

### Multivariate Analysis (Heatmap Analysis and K-Means Clustering)

Heatmap analysis of metabolome data was conducted using the R package pheatmap under R version 3.6.1. K-means clustering of time-course transcriptome data was performed using iDEP90 (Ge et al., 2018).

### cAMP Addition and Analysis of the Culture Extracts by LC-QTOF MS

cAMP (10 μM) was supplemented to culture of M1146 at the mid–log phase, and culture was sampled at 72 h. Medium (20 mL) was vortexed well with 20 mL ethyl acetate. After centrifugation (10,000 rpm, 4°C) for 10 min, the 20 mL supernatant was subjected to centrifugal concentration and lyophilized by freeze drying. The lyophilized sample was reconstituted with 400 μL 50% acetonitrile, and the 40 μL solution was transferred to LC vial and subjected to LC-QTOF MS analysis. LC-QTOF MS 9030 (Shimadzu) was used for secondary metabolite detection and Nexera XR UHPLC (Shimadzu), and the Inert Sustain AQ-C18 (3 μm, 150 × 2.1 mm; GLscience) column was used for chromatographic separation. Formic acid 0.1% in ultrapure water (vol/vol) was used for mobile phase A. whereas acetonitrile with 0.1% formic acid (vol/vol) was used for mobile phase B. The chromatographic separation was performed by gradient mode. Gradient condition is as follows. The ratio of mobile phase B was kept at 0% for 3 min, raised until 100% by 8 min, and kept at 100% for 2 min. The B ratio was reduced to 0% by 3 min and kept at 0% for the next analysis. The flow rate was set to 0.2 mL/min. Column oven temperature was set to 40°C. The injection volume was set to 3 μL. For mass spectrometer parameter, the following settings were used. Interface voltage was set to 4 kV, needle voltage was set to 4.5 kV, flow rate of nebulizer gas was set to 2.0 L/min, flow rate of heating gas was set to 10 L/min, flow rate of drying gas was set to 10 L/min, interface temperature was set to 300°C, DL temperature was set to 250°C, and heat block temperature was set to 250°C. Metabolites were ionized in positive ionization mode by DUIS (dual ion source) mode. For secondary metabolite peak discovery, TOF *m/z* range was set to 100 to 700. For specific germicidin analysis, TOF *m/z* range was set to 100–250. For MS/MS analysis, collision energy was set to -15 eV.

## Data Availability Statement

The datasets presented in this study can be found in online repositories. The metabolome analysis data and transcriptome analysis data were uploaded to public data repositories “Metabolights database” and “NCBI GEO database”. The accession numbers can be found in the article.

## Author Contributions

EF, SP, ET, and RB conceived the idea of this study. The experiments were designed by all authors. All experiments were performed by KN. The data interpretation was done by KN, ET, SP, RB, and FC. The manuscript was written by all authors. All authors read and approved the final manuscript.

## Conflict of Interest

The authors declare that the research was conducted in the absence of any commercial or financial relationships that could be construed as a potential conflict of interest.
