# Supplementary material for: Multi-Omics Analysis of the Effect of cAMP on Actinorhodin Production in Streptomyces coelicolor
Source: Front Bioeng Biotechnol. 2020 Nov 5;8:595552. doi: 10.3389/fbioe.2020.595552 (PMC7674942; doi:10.3389/fbioe.2020.595552)
Supplement: Supplementary file 2 [file Data_Sheet_2.docx]

**Supplementary Materials**

Multi-omics analysis of the effect of cAMP on actinorhodin production in *Streptomyces coelicolor*

Katsuaki Nitta^1^, Francesco Del Carratore^2^, Rainer Breitling^2^, Eriko Takano^2^*, Sastia P. Putri^1^*, Eiichiro Fukusaki^1^

1: Department of Biotechnology, Graduate School of Engineering, Osaka University, Osaka, Japan

2: Manchester Institute of Biotechnology, Manchester Synthetic Biology Research Centre SYNBIOCHEM, Department of Chemistry, The University of Manchester, Manchester, UK


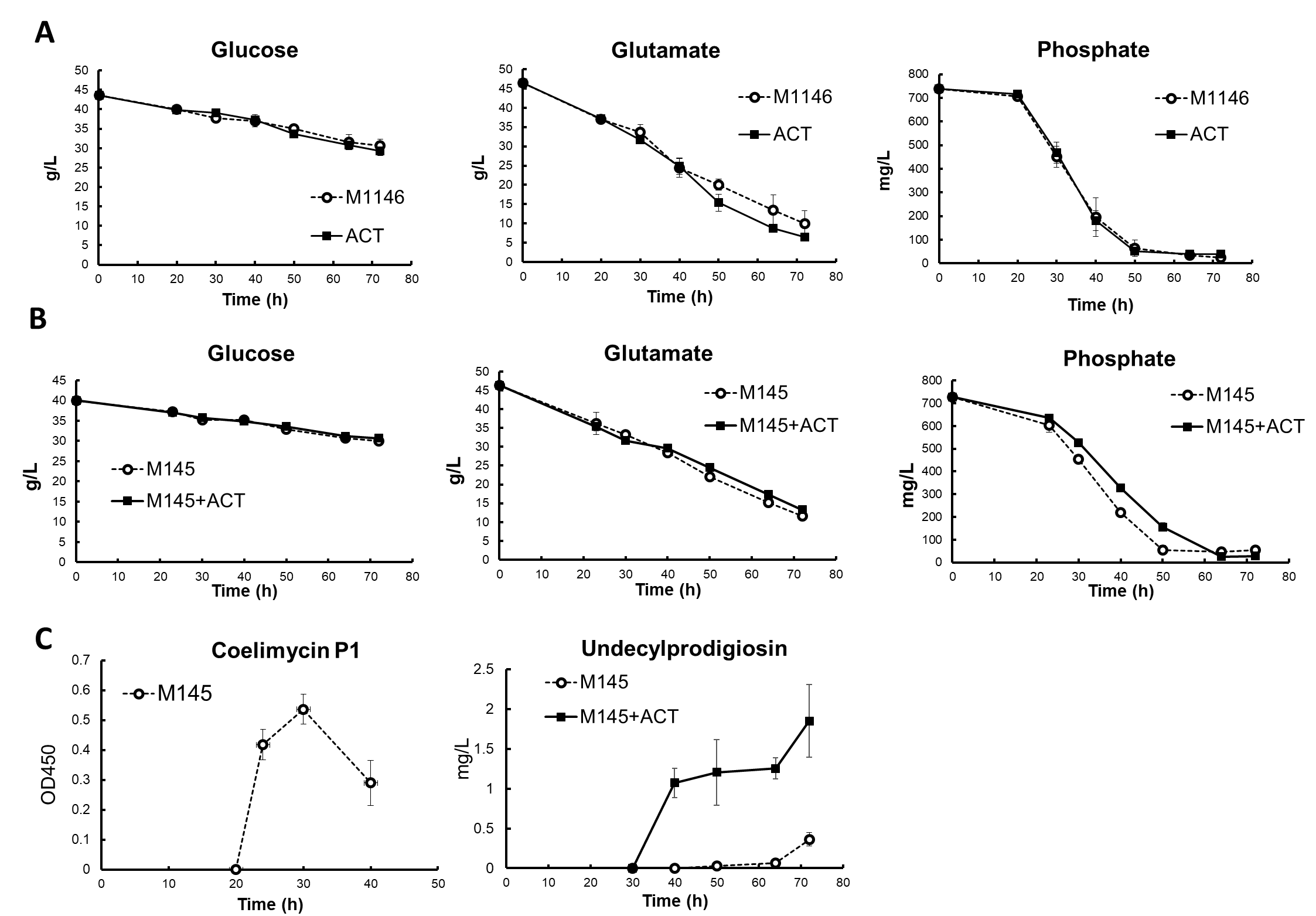


**Supplementary Figure S1. Time-course of the consumption of medium components in four strains M145, M145+ACT, M1146 and M1146+ACT**

(A) Consumption of medium components (glucose, glutamate, phosphate) during growth of M1146 (dotted line chart) and M1146+ACT (black line chart). Error bars indicate the standard deviation among three replicates.

(B) Consumption of medium components (glucose, glutamate, phosphate) during growth of M145 (dotted line chart) and M145+ACT (black line chart). Error bars indicate the standard deviation among three replicates.

(C) Coelimycin P1 production in M145 and Undecylporodigiosin production in M145 (dotted line chart) and M145+ACT (black line chart).


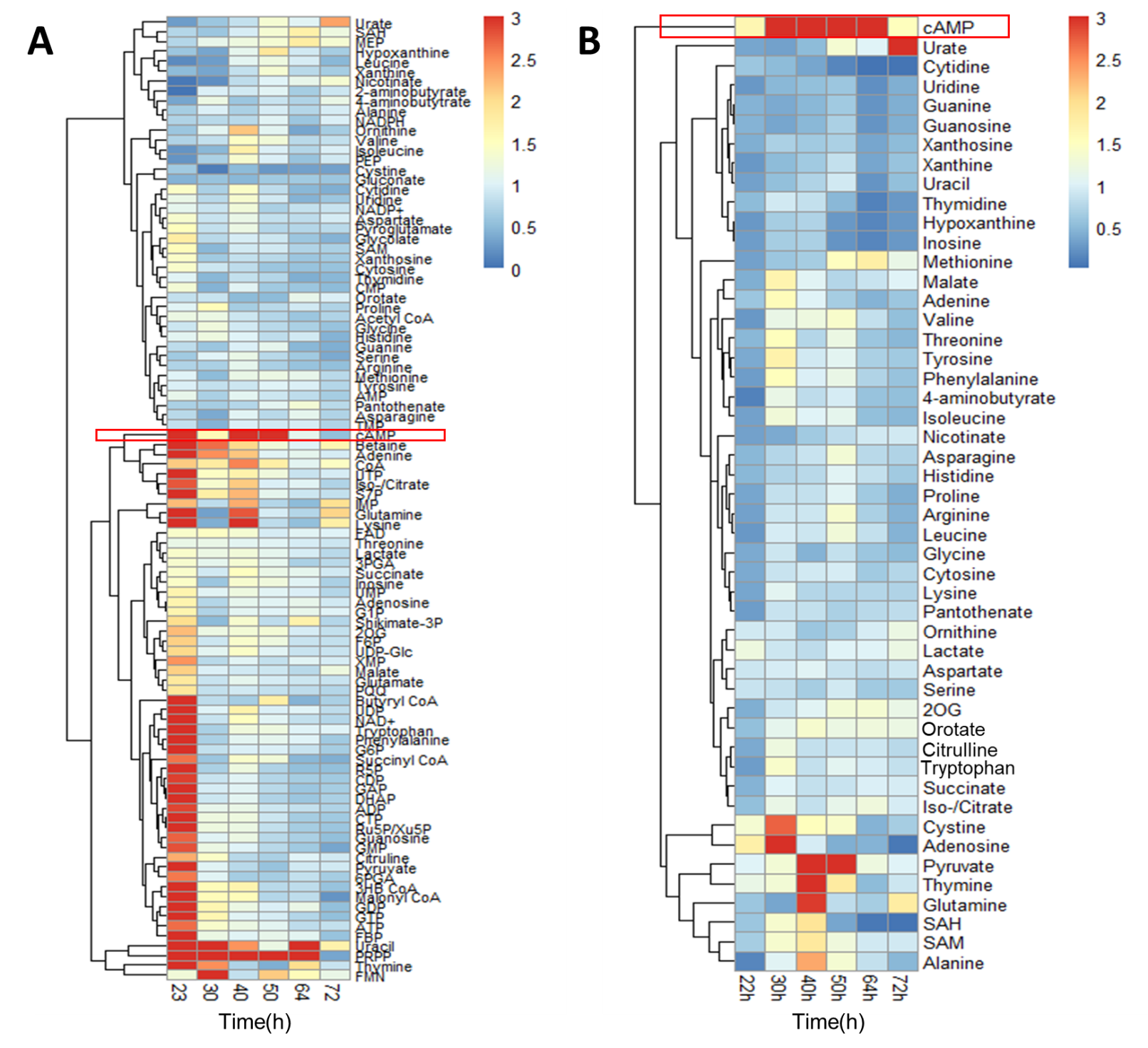


**Supplementary Figure S2.** **Heat map of relative metabolite levels during the growth of M145 and M145+ACT.**

Relative levels are indicated as fold-changes of M145 compared to M145+ACT (red: higher levels in M145+ACT, blue: lower levels in M145+ACT). cAMP results are boxed in red.

1. Intracellular metabolite levels (B) Extracellular metabolite levels.


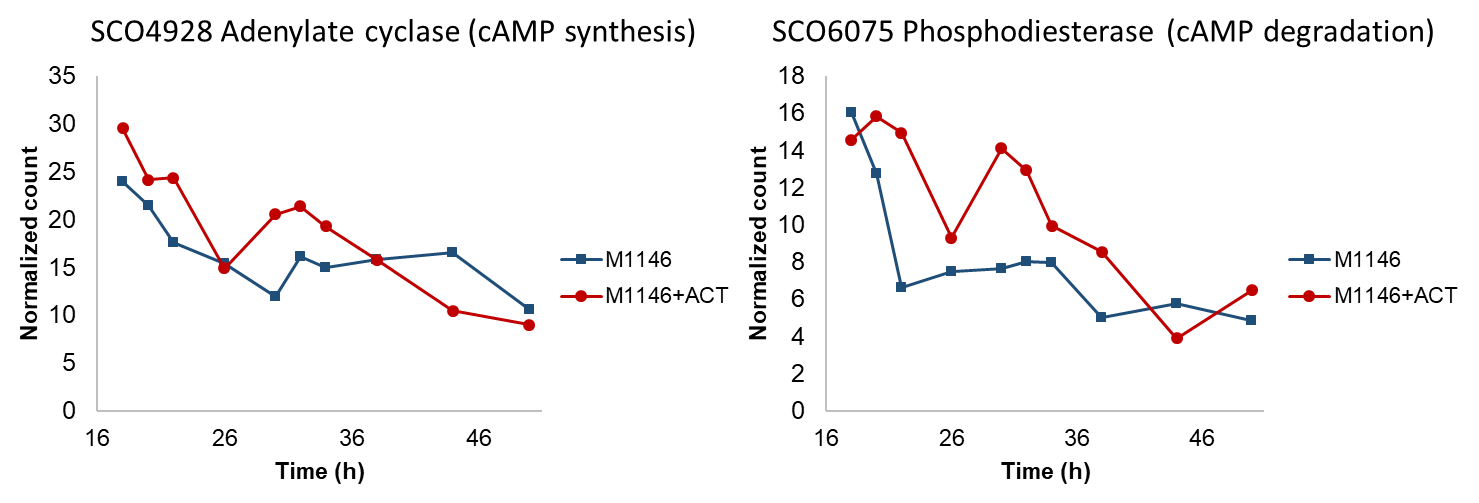


**Supplementary Figure S3 Gene expression level comparison of adenylate cyclase and phosphodiesterase between M1146 and M1146+ACT**

Blue line shows gene expression changes in M1146 while red line shows gene expression level in M1146+ACT. TPM normalized count was used to create the line chart graph


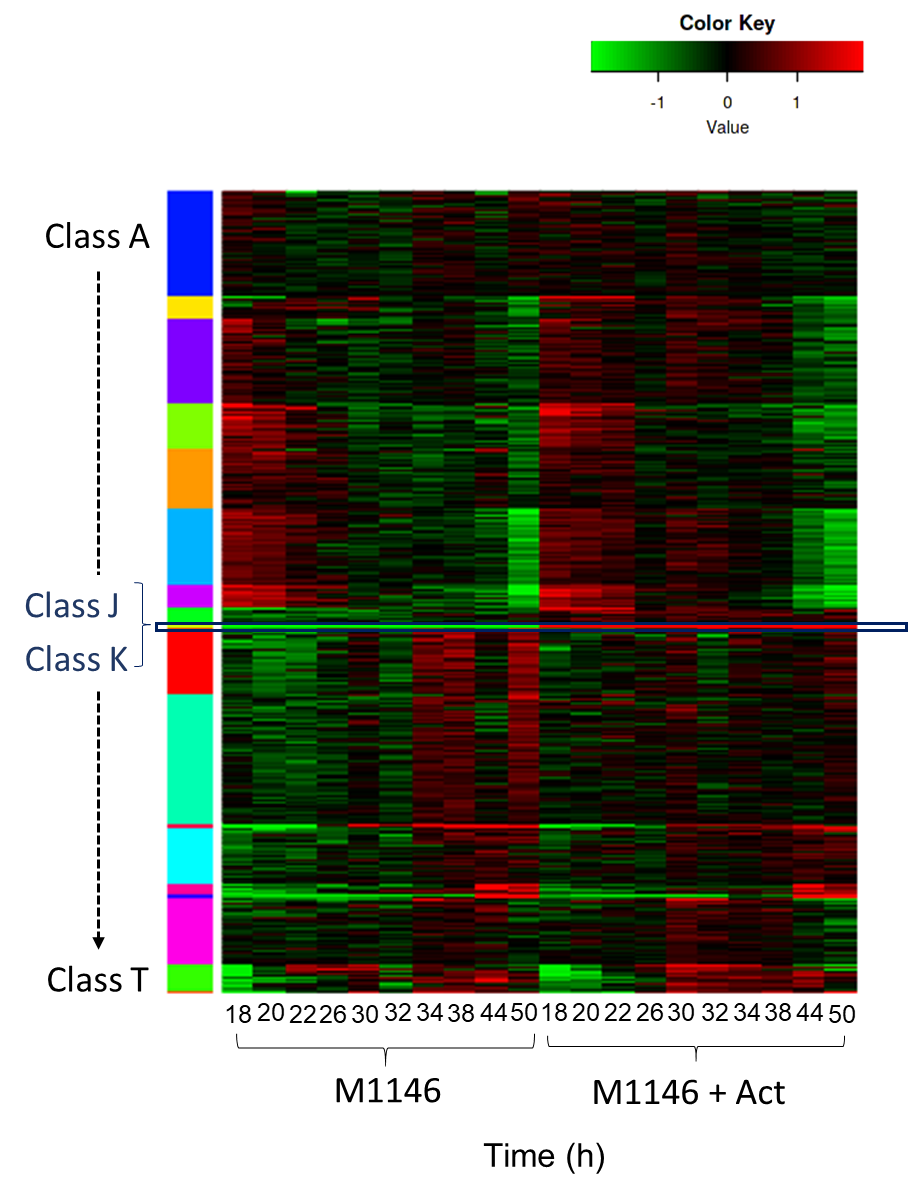


**Supplementary Figure S4 k-means clustering of time-course transcriptome analysis of M1146 and M1146+ACT**

k-means clustering of transcriptome analysis of M1146 and M1146+ACT (k=20). The read counts data from RNAseq were normalized by z-score. Genes from class J and class K are boxed in blue. The clustering information is shown in supplementary Excel file 1.


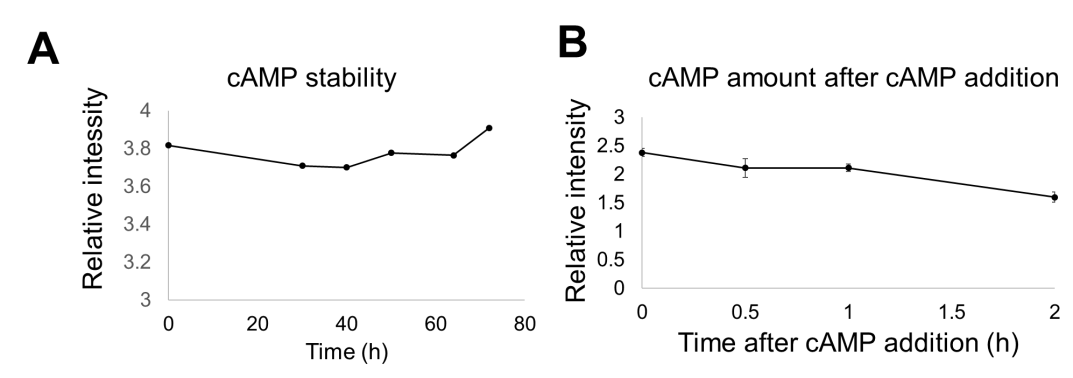


**Supplementary Figure S5.** **cAMP stability and cAMP uptake**

(A) cAMP stability in cell-free medium from 0h to 72h of incubation at 30°C.

(B) cAMP uptake by a growing culture of M1146. Error bars indicate standard deviation among three replicates.


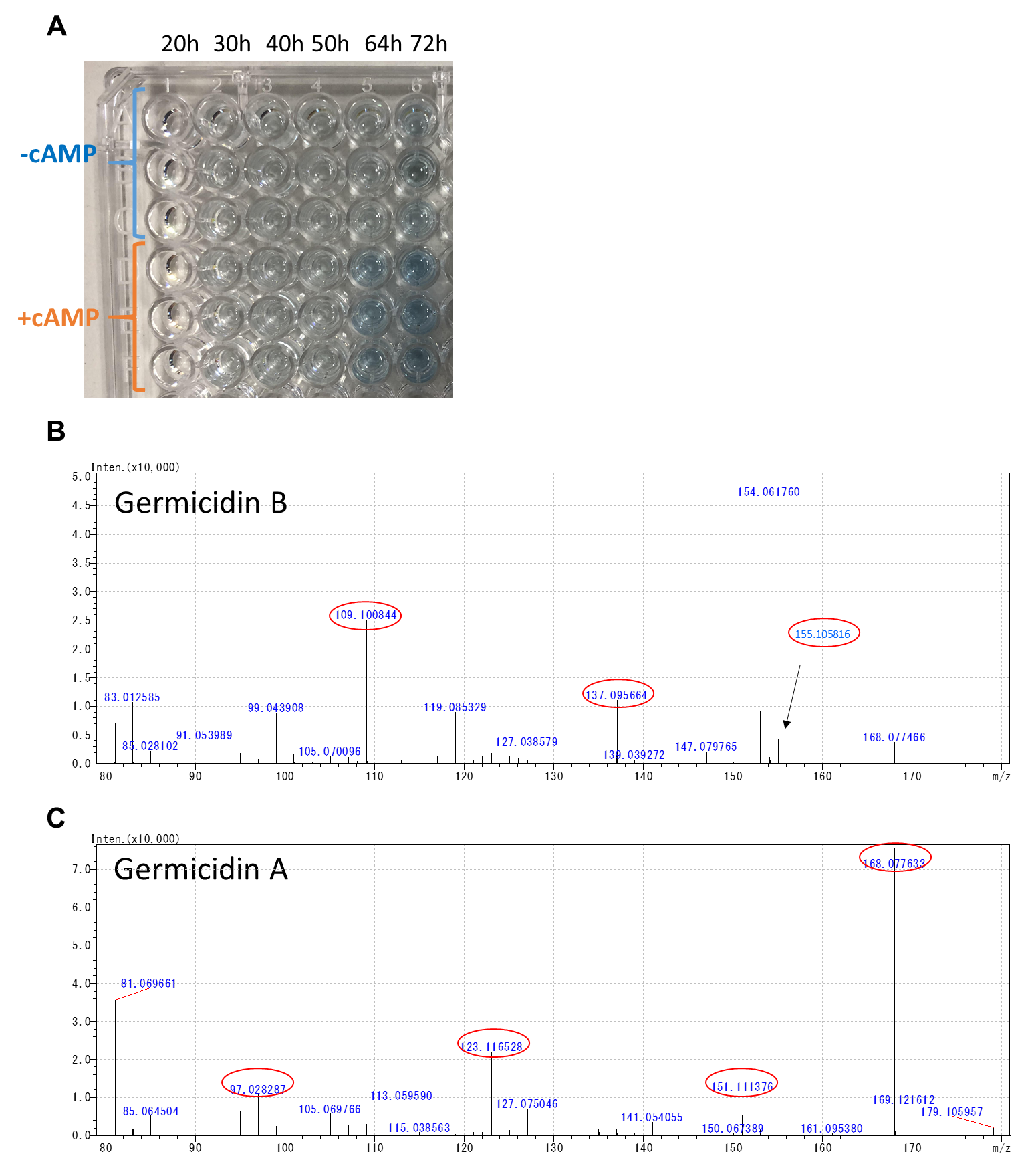


**Supplementary Figure S6. Intracellular actinorhodin production and MS/MS spectrum of two germicidins**

(A) Intracellular actinorhodin production by M1146+ACT, indicated by the presence of blue color, after addition of cAMP during growth.

(B) MS/MS spectrum of Germicidin B extracted from M1146 after addition of cAMP. The collision energy was set to −15 eV. Ions circled in red were observed in a previous study.

(C) MS/MS spectrum of Germicidin A extracted from M1146 after addition of cAMP. The collision energy was set to −15 eV. Ions circled in red were observed in a previous study.


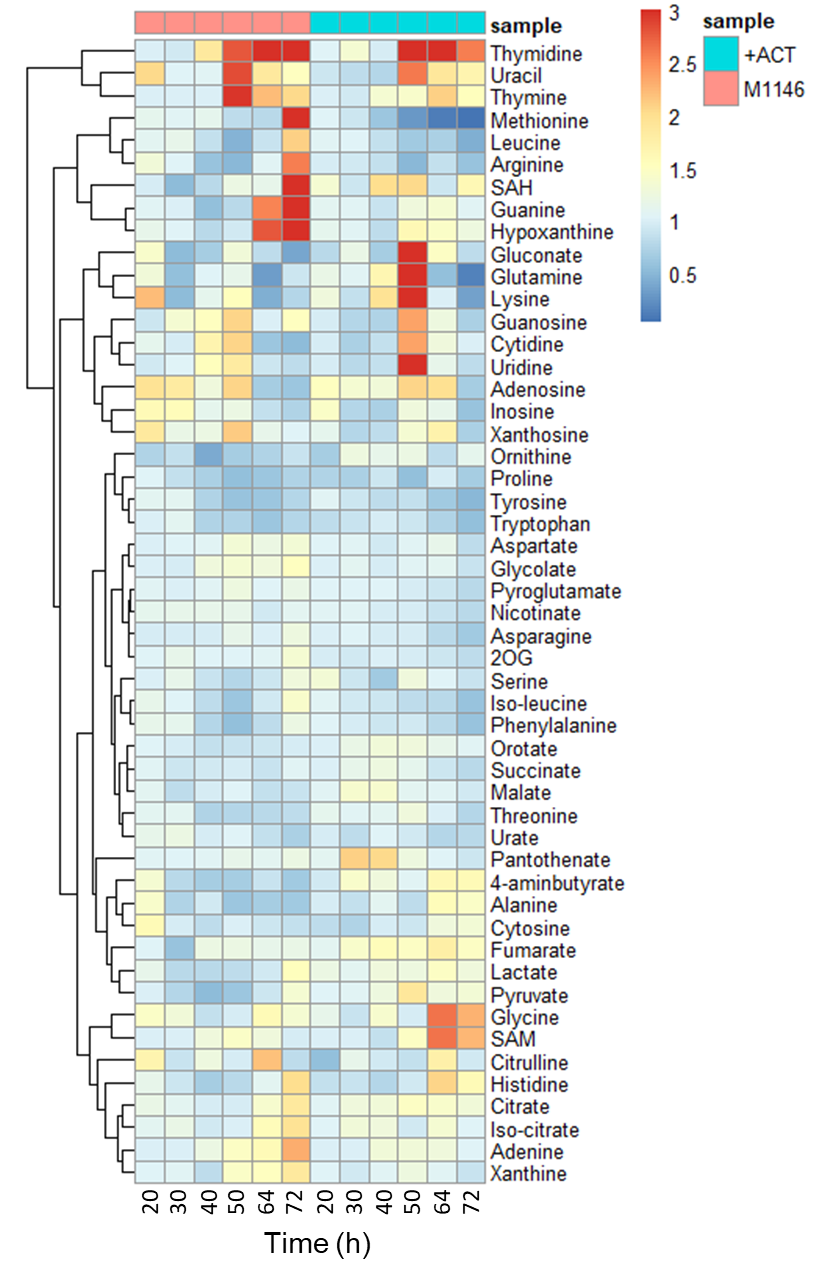


**Supplementary Figure S7. Extracellular metabolome analysis of cAMP supplemented M1146 and M1146+ACT during growth.**

The metabolites amount difference is shown by fold-change of area value (cAMP supplemented/non-cAMP supplemented) in heat map clustering. Average value from three replicates were used to calculate the fold-change value. This heat map consists of data of cAMP supplemented of M1146 (pink – left 6 panels) and M1146+ACT (blue – right 6 panels). The metabolites are listed in Supplementary Table S2.


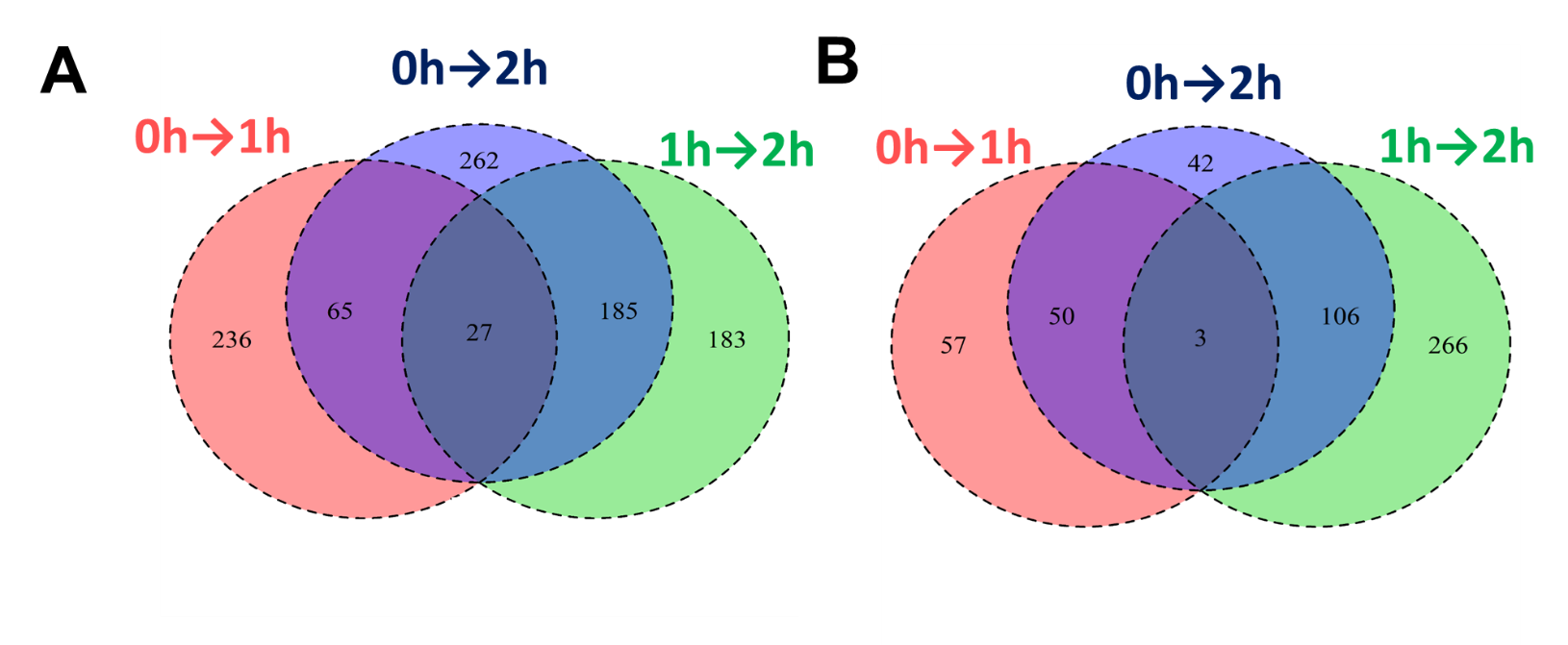


**Supplementary Figure S8 Summary of RNAseq analysis of cAMP supplemented M1146.** See main text and Supplementary Table S8 for details.

(A) Venn diagram of upregulated genes after cAMP supplementation.

(B) Venn diagram of downregulated genes after cAMP supplementation.

**Supplementary Table S1. Strains used in this study**

| Name | Relevant characteristics | Reference |
| --- | --- | --- |
| **Strain** |  |  |
| M145 | SCP1- SCP2- derivative of *S.coelicolor* A3(2) | Kieser et al., 2000 |
| M1146 | M145 Δ*act* Δ*red* Δ*cpk* Δ*cda* | Gomez-Escribano et al., 2010 |
| M145+ACT | M145 + pAH88 | Gomez-Escribano et al., 2010 |
| M1146+ACT | M1146 + pAH88 | Gomez-Escribano et al., 2010 |
| **Plasmid** |  |  |
| pAH88 | BAC derivative SCBAC28G1 containing Act gene cluster | Gomez-Escribano et al., 2010 |

**Supplementary Table S2 Analyzed metabolites in this study**

| **Amino acids** | **Organic acids** | **Base, nucleoside, nucleotide** | **Sugar-phosphate** | **Others** |
| --- | --- | --- | --- | --- |
| Serine | Urate | Adenine | G6P | NAD+ |
| Glutamine | Lactate | Guanine | R5P | NADP+ |
| Threonine | Pyruvate | Cytosine | S7P | MEP |
| Valine | Succinate | Thymine | F6P | Pantothenate |
| Tyrosine | Malate | Uracil | GAP | Nicotinate |
| Methionine | 2OG | Hypoxanthine | Ru5P | UDP-Glc |
| Isoleucine | Fumarate | Xanthine | F1P | Orotate |
| Leucine | Cirtrate | Adenosine | R1P | FMN |
| Glutamate | Iso-citrate | Guanosine  Cytidine | DHAP | FAD |
| Aspartate | 2-isopropylmalate |  | 6PGA | PRPP |
| Phenylalanine | Gluconate | Uridine | 3PGA/2PGA | PQQ |
| Pyroglutamate |  | Thymidine | FBP | CoA |
| Tryptophan |  | Inosine | PEP | Malonyl-CoA |
| Cystine |  | Xanthosine |  | Acetyl-CoA |
| Asparagine |  | AMP |  | Succinyl-CoA |
| Glycine |  | GMP |  | Butyryl-CoA |
| Alanine |  | CMP |  | shikimate-3P* |
| Citrulline |  | TMP |  |  |
| Betaine |  | UMP |  |  |
| Proline |  | IMP |  |  |
| Ornithine |  | XMP |  |  |
| Histidine |  | ADP |  |  |
| Lysine |  | GDP |  |  |
| Arginine |  | CDP |  |  |
| 4-aminobutyrate |  | UDP |  |  |
| 2-aminobutyrate |  | ATP |  |  |
| SAM |  | GTP |  |  |
| SAH |  | CTP |  |  |
|  |  | UTP |  |  |

*Not detected in metabolome analysis in Fig.6

**Supplementary Table S3 ACT gene cluster expression level compared in M1146 and M1146+ACT**

| gene name | Locus_tag | Product | log2FoldChange | FDR |
| --- | --- | --- | --- | --- |
| actVA4 | SCO5079 | hypothetical protein | 10.67202632 | 7.53E-61 |
| actIORF1 | SCO5087 | actinorhodin polyketide beta-ketoacyl synthase subunit alpha | 10.60719203 | 1.95E-55 |
| actIORF3 | SCO5089 | actinorhodin polyketide synthase | 10.54600035 | 8.52E-59 |
| actVA3 | SCO5078 | hypothetical protein | 10.51897457 | 5.41E-72 |
| actII-4 | SCO5085 | actinorhodin operon activator protein | 10.44942593 | 5.06E-119 |
| SCBAC20F6.17 | SCO5074 | dehydratase | 10.27604203 | 1.41E-75 |
| actIII | SCO5086 | ketoacyl reductase | 10.07689616 | 7.88E-88 |
| ORF4 | SCO5075 | oxidoreductase | 10.06820461 | 1.22E-118 |
| actVA6 | SCO5081 | hypothetical protein | 9.944051646 | 2.86E-46 |
| actIORF2 | SCO5088 | actinorhodin polyketide beta-ketoacyl synthase subunit beta | 9.914040984 | 3.33E-71 |
| actII-3 | SCO5084 | hypothetical protein | 9.895801902 | 2.07E-107 |
| actVA1 | SCO5076 | hypothetical protein | 9.892664608 | 1.38E-223 |
| actVA2 | SCO5077 | hypothetical protein | 9.863269995 | 3.61E-82 |
| actII-2 | SCO5083 | actinorhodin transporter | 9.627593745 | 7.36E-236 |
| actII-1 | SCO5082 | transcriptional regulator | 9.258018976 | 2.07E-164 |
| actVA5 | SCO5080 | hydrolase | 8.891071482 | 4.58E-122 |
| SCBAC20F6.16 | SCO5073 | oxidoreductase | 8.841816949 | 2.88E-221 |
| actVII | SCO5090 | actinorhodin polyketide synthase bifunctional cyclase/dehydratase | 7.976746342 | 3.98E-187 |
| SCBAC20F6.14c | SCO5071 | hydroxylacyl-CoA dehydrogenase | 7.52520911 | 3.70E-184 |
| actVB | SCO5092 | actinorhodin polyketide dimerase | 7.18577353 | 3.81E-145 |
| SCBAC20F6.15 | SCO5072 | hydroxylacyl-CoA dehydrogenase | 7.101926221 | 4.59E-239 |
| actIV | SCO5091 | cyclase | 6.953515873 | 3.59E-161 |
| SCBAC20F6.13c | SCO5070 | hydroxylacyl-CoA dehydrogenase | 4.60245964 | 7.85E-81 |
| ORF9 | SCO5095 | DNA-binding protein | 1.506216245 | 1.91E-15 |
| SCBAC28G1.29 | SCO5103 | regulatory protein | 0.982746855 | 1.67E-10 |
| SCBAC20F6.03c | SCO5060 | hypothetical protein | 0.973599023 | 6.40E-19 |
| SCBAC20F6.12 | SCO5069 | oxidoreductase | 0.963365034 | 2.85E-10 |
| SCBAC20F6.08c | SCO5065 | transcriptional regulator | 0.927815504 | 1.53E-11 |
| SCBAC20F6.09 | SCO5066 | beta-lactamase | 0.927487683 | 2.40E-06 |
| SCBAC28G1.28 | SCO5102 | mutT-like protein | 0.90661064 | 9.32E-09 |
| ispH | SCO5058 | 4-hydroxy-3-methylbut-2-enyl diphosphate reductase | 0.891435788 | 7.50E-19 |
| SCBAC28G1.30 | SCO5104 | hypothetical protein | 0.887510805 | 5.57E-16 |
| ppgK | SCO5059 | polyphosphate glucokinase | 0.853336599 | 2.02E-21 |
| SCBAC20F6.10 | SCO5067 | carboxylesterase | 0.811774781 | 0.003275409 |
| SCBAC28G1.26 | SCO5100 | GntR family transcriptional regulator | 0.80600886 | 3.16E-11 |
| ychF | SCO5061 | GTP-dependent nucleic acid-binding protein EngD | 0.795743072 | 1.04E-07 |
| SCBAC20F6.07 | SCO5064 | hypothetical protein | 0.739162933 | 1.48E-28 |
| SCBAC28G1.23 | SCO5097 | short-chain oxidoreductase | 0.705610289 | 1.25E-06 |
| SCBAC28G1.27 | SCO5101 | hypothetical protein | 0.670693538 | 3.40E-06 |
| SCBAC28G1.25 | SCO5099 | hypothetical protein | 0.620187399 | 2.00E-12 |
| SCBAC20F6.11c | SCO5068 | TetR family transcriptional regulator | 0.491076237 | 0.002505519 |
| SCBAC28G1.22c | SCO5096 | LysR family transcriptional regulator | 0.48180961 | 0.003249599 |
| SCK7.30c | SCO5057 | amino acid permease | 0.411141245 | 0.002353811 |

**Supplementary Table S4 List of DEGs in time-course RNAseq analysis of M1146 and M1146+ACT**

|  | Locus_tag | Log2 fold-change | FDR | product |
| --- | --- | --- | --- | --- |
| Upregulated | SCO0320 | 5.399 | 4.16E-72 | hypothetical protein |
|  | SCO4266 | 4.506 | 7.47E-105 | oxidoreductase |
|  | SCO1909 | 4.213 | 1.35E-105 | hypothetical protein |
|  | SCO2478 | 4.142 | 6.90E-71 | reductase |
|  | SCO1178 | 4.044 | 2.56E-119 | hypothetical protein |
|  | SCO0321 | 3.346 | 8.67E-64 | carboxylesterase |
|  | SCO4265 | 2.997 | 1.45E-73 | transport integral membrane protein |
|  | SCO7008 | 2.828 | 6.99E-48 | ABC transporter ATP-binding protein |
|  | SCO2477 | 2.465 | 1.36E-23 | short chain dehydrogenase |
|  | SCO5285 | 1.765 | 2.90E-16 | ATP-dependent protease |
|  | SCO4280 | 1.709 | 8.75E-28 | reductase |
|  | SCO5163 | 1.676 | 2.09E-08 | hypothetical protein |
|  | SCO0379 | 1.653 | 2.83E-11 | catalase |
|  | SCO0224 | 1.651 | 1.93E-23 | hypothetical protein |
|  | SCO2695 | 1.589 | 1.01E-25 | hypothetical protein |
|  | SCO0380 | 1.565 | 8.07E-13 | hypothetical protein |
|  | SCO2027 | 1.412 | 1.78E-15 | hypothetical protein |
|  | SCO3206 | 1.397 | 1.56E-16 | transmembrane efflux protein |
|  | SCO6810 | 1.325 | 4.80E-08 | hypothetical protein |
|  | SCO2476 | 1.302 | 3.84E-08 | dehydrogenase/reductase |
|  | SCO0888 | 1.298 | 4.59E-07 | hypothetical protein |
|  | SCO1366 | 1.188 | 6.56E-11 | hypothetical protein |
|  | SCO1802 | 1.177 | 2.18E-12 | two-component system sensor kinase |
|  | SCO4174 | 1.153 | 2.80E-02 | hypothetical protein |
|  | SCO4279 | 1.125 | 7.91E-13 | acetyltransferase |
|  | SCO1176 | 1.080 | 3.01E-11 | dihydroxy-acid dehydratase |
|  | SCO4983 | 1.035 | 2.11E-05 | hypothetical protein |
|  | SCO3207 | 1.028 | 6.76E-06 | TetR family transcriptional regulator |
|  | SCO6811 | 1.023 | 5.73E-05 | hypothetical protein |
|  | SCO1801 | 0.994 | 4.47E-09 | two component response regulator |
|  | SCO1661 | 0.991 | 1.72E-02 | glycerol-3-phosphate dehydrogenase |
|  | SCO1044 | 0.953 | 3.72E-02 | hypothetical protein |
|  | SCO2309 | 0.940 | 7.51E-11 | transmembrane transport protein |
|  | SCO1245 | 0.894 | 3.57E-04 | adenosylmethionine--8-amino-7-oxononanoate aminotransferase BioA |
|  | SCO7536 | 0.874 | 9.13E-06 | hypothetical protein |
|  | SCO4336 | 0.855 | 3.29E-06 | marR-family protein |
|  | SCO3366 | 0.851 | 2.87E-08 | exporter |
|  | SCO1145 | 0.826 | 6.34E-12 | MarR family regulatory protein |
|  | SCO6694 | 0.824 | 1.54E-06 | transcriptional regulator |
|  | SCO5485 | 0.814 | 9.26E-03 | small hydrophobic membrane protein |
|  | SCO1814 | 0.813 | 5.27E-06 | enoyl-ACP reductase |
|  | SCO6692 | 0.809 | 2.88E-04 | hypothetical protein |
|  | SCO0999 | 0.807 | 9.81E-04 | superoxide dismutase |
|  | SCO1089 | 0.803 | 9.41E-07 | hypothetical protein |
|  | SCO0955 | 0.797 | 1.07E-02 | hypothetical protein |
|  | SCO4587 | 0.791 | 2.11E-05 | hypothetical protein |
|  | SCO7305 | 0.779 | 6.95E-04 | hypothetical protein |
|  | SCO1046 | 0.758 | 2.44E-02 | metal transporter ATPase |
|  | SCO1803 | 0.757 | 4.86E-03 | oxidoreductase |
|  | SCO1304 | 0.753 | 8.18E-08 | hypothetical protein |
|  | SCO3694 | 0.740 | 2.03E-02 | transcriptional regulator |
|  | SCO1244 | 0.736 | 4.37E-03 | biotin synthase |
|  | SCO6520 | 0.732 | 2.86E-03 | RNA polymerase sigma factor |
|  | SCO2937 | 0.712 | 5.62E-03 | transmembrane transport protein |
|  | SCO2696 | 0.711 | 9.81E-04 | 2-hydroxyacid dehydrogenase |
|  | SCO1550 | 0.709 | 1.88E-03 | small membrane protein |
|  | SCO1247 | 0.709 | 6.28E-06 | hypothetical protein |
|  | SCO5484 | 0.705 | 3.50E-02 | small hydrophobic membrane protein |
|  | SCO4337 | 0.702 | 9.57E-05 | integral membrane efflux protein |
|  | SCO5031 | 0.698 | 5.59E-04 | hypothetical protein |
|  | SCO6693 | 0.686 | 4.10E-04 | hypothetical protein |
|  | SCO0887 | 0.684 | 1.67E-04 | TetR family transcriptional regulator |
|  | SCO5032 | 0.684 | 6.18E-04 | alkyl hydroperoxide reductase |
|  | SCO0885 | 0.683 | 9.76E-13 | thioredoxin |
|  | SCO3800 | 0.682 | 2.66E-02 | acyl-CoA dehydrogenase |
|  | SCO6828 | 0.678 | 1.11E-06 | hypothetical protein |
|  | SCO2634 | 0.667 | 6.68E-03 | hypothetical protein |
|  | SCO5206 | 0.663 | 1.60E-04 | hydrogen peroxide sensitive repressor |
|  | SCO7314 | 0.661 | 3.30E-04 | RNA polymerase sigma factor |
|  | SCO1804 | 0.655 | 1.22E-02 | S-adenosylmethionine:tRNA ribosyltransferase-isomerase |
|  | SCO2763 | 0.650 | 1.54E-02 | ABC transporter ATP-binding protein |
|  | SCO1905 | 0.648 | 3.92E-02 | hypothetical protein |
|  | SCO1246 | 0.643 | 2.54E-02 | dithiobiotin synthetase |
|  | SCO5490 | 0.641 | 1.69E-04 | hypothetical protein |
|  | SCO0463 | 0.639 | 1.24E-04 | hypothetical protein |
|  | SCO4297 | 0.639 | 2.03E-02 | oxidoreductase |
|  | SCO4501 | 0.631 | 1.43E-04 | 3-ketoacyl-ACP reductase |
|  | SCO5535 | 0.630 | 2.46E-04 | carboxyl transferase |
|  | SCO2498 | 0.624 | 7.03E-03 | efflux protein |
|  | SCO1084 | 0.620 | 5.89E-04 | thioredoxin |
|  | SCO5536 | 0.616 | 6.68E-03 | hypothetical protein |
|  | SCO5042 | 0.614 | 4.66E-09 | fumarate hydratase |
|  | SCO3111 | 0.612 | 1.54E-02 | ABC transporter ATP-binding protein |
|  | SCO3286 | 0.609 | 5.59E-04 | hypothetical protein |
|  | SCO0973 | 0.605 | 1.43E-05 | hypothetical protein |
|  | SCO1997 | 0.600 | 1.86E-05 | hypothetical protein |
|  | SCO1144 | 0.597 | 2.90E-05 | ABC transporter ATP-binding protein |
|  | SCO7784 | 0.596 | 2.73E-04 | oxidoreductase |
|  | SCO3110 | 0.567 | 3.50E-02 | ABC transporter |
|  | SCO6354 | 0.567 | 1.90E-03 | two-component regulator |
|  | SCO2816 | 0.554 | 1.23E-02 | hypothetical protein |
|  | SCO7226 | 0.546 | 1.99E-03 | hypothetical protein |
|  | SCO6515 | 0.535 | 4.88E-02 | protease |
|  | SCO6127 | 0.533 | 5.62E-03 | carboxylesterase |
|  | SCO1243 | 0.529 | 2.66E-02 | 8-amino-7-oxononanoate synthase |
|  | SCO4956 | 0.520 | 2.35E-03 | methionine sulfoxide reductase A |
|  | SCO2745 | 0.514 | 4.57E-02 | LacI family transcriptional regulator |
|  | SCO5191 | 0.511 | 1.72E-02 | hypothetical protein |
|  | SCO0917 | 0.507 | 2.30E-02 | oxygenase |
|  | SCO7442 | 0.507 | 1.57E-02 | hypothetical protein |
| Downregulated | SCOt14 | -0.508 | 4.73E-02 | tRNA-Ala |
|  | SCO4744 | -0.531 | 1.64E-02 | 4'-phosphopantetheinyl transferase |
|  | SCO4949 | -0.560 | 1.90E-02 | nitrate reductase subunit delta NarJ3 |
|  | SCO6296 | -0.567 | 3.10E-03 | hypothetical protein |
|  | SCOt59 | -0.577 | 6.16E-03 | tRNA-Gln |
|  | SCO1684 | -0.578 | 2.06E-02 | hypothetical protein |
|  | SCO4748 | -0.603 | 4.39E-02 | hypothetical protein |
|  | SCO1334 | -0.608 | 4.70E-02 | hypothetical protein |
|  | SCO0141 | -0.678 | 4.55E-03 | calcium-binding protein |
|  | SCO6992 | -0.749 | 3.68E-02 | regulatory protein |
|  | SCO1368 | -0.755 | 1.11E-02 | ABC transporter |
|  | SCO6886 | -0.775 | 3.55E-02 | hypothetical protein |
|  | SCOt65 | -0.804 | 1.82E-07 | tRNA-Pro |
|  | SCOt63 | -0.812 | 2.52E-12 | tRNA-Pro |
|  | SCOt64 | -0.856 | 4.47E-10 | tRNA-Pro |

**Supplementary Table S5 Gene set enrichment analysis of DEGs in time-course RNAseq analysis of M1146 and M1146+ACT**

| Class | Gene set | Gene list |
| --- | --- | --- |
| Biological process | biotin biosynthetic process | SCO1246 |
|  | (p-value: 1.10E-03) | SCO1245 |
|  |  | SCO1244 |
|  | oxidation-reduction process | SCO6811 |
|  | (p-value: 7.80E-03) | SCO1084 |
|  |  | SCO4297 |
|  |  | SCO0885 |
|  |  | SCO6828 |
|  | magnesium ion transport | SCO6692 |
|  | (p-value: 4.10E-02) | SCO0973 |
|  | transmembrane transport | SCO3366 |
|  | (p-value: 5.00E-02) | SCO4265 |
|  |  | SCO2309 |
|  |  | SCO2937 |
|  |  | SCO2498 |
|  |  | SCO3206 |
|  | cell redox homeostasis | SCO5032 |
|  | (p-value: 5.60E-02) | SCO1084 |
|  |  | SCO0885 |
|  | cellular response to oxidative stress | SCO1084 |
|  | (p-value: 6.70E-02) | SCO0885 |
|  | sulfate assimilation | SCO1084 |
|  | (p-value: 8.00E-02) | SCO0885 |
| Pathway | biotin metabolism | SCO1246 |
|  | (p-value: 6.30E-08) | SCO1245 |
|  |  | SCO1244 |
|  |  | SCO4501 |
|  |  | SCO1814 |
|  |  | SCO1243 |
| Keywords | biotin biosynthesis | SCO1246 |
|  | (p-value: 4.30E-04) | SCO1245 |
|  |  | SCO1244 |
|  | oxidoreductase | SCO0999 |
|  | (p-value: 6.00E-02) | SCO2696 |
|  |  | SCO5031 |
|  |  | SCO4956 |
|  |  | SCO1814 |
|  |  | SCO3800 |
|  |  | SCO0379 |
|  | S-adenosyl-L-methionine | SCO1245 |
|  | (p-value: 6.70E-02) | SCO1804 |
|  |  | SCO1244 |

**Supplementary Table S6 List of DEGs in cAMP supplementation RNAseq of M145 (WT)**

|  | Locus_tag | log2  FoldChange | FDR | Product |
| --- | --- | --- | --- | --- |
| Upregulated | SCO5086 | 1.612 | 1.01E-03 | ketoacyl reductase |
|  | SCO1803 | 1.601 | 1.64E-02 | oxidoreductase |
|  | SCO4468 | 1.400 | 1.83E-03 | hypothetical protein |
|  | SCO1804 | 1.350 | 1.54E-02 | S-adenosylmethionine:tRNA ribosyltransferase-isomerase |
|  | SCO7665 | 1.331 | 8.43E-04 | hypothetical protein |
|  | SCO5267 | 1.211 | 1.15E-02 | hypothetical protein |
|  | SCO2265 | 1.082 | 3.94E-02 | hypothetical protein |
|  | SCO6632 | 1.068 | 6.29E-04 | hypothetical protein |
|  | SCO1421 | 1.041 | 7.20E-03 | hypothetical protein |
|  | SCO1470 | 1.030 | 2.66E-02 | hypothetical protein |
|  | SCOs02 | 0.974 | 7.20E-03 | Note=ssrA gene coding for tmRNA%2C 397 bp%3B similar to SW:MT10SARNA (EMBL:X60301) Mycobacterium tuberculosis gene for 10Sa RNA%2C 606 bp%3B |
|  | SCO3663 | 0.952 | 6.29E-04 | hypothetical protein |
|  | SCO5085 | 0.947 | 4.09E-02 | actinorhodin operon activator protein |
|  | SCO3273 | 0.901 | 2.20E-02 | hypothetical protein |
|  | SCO3929 | 0.881 | 2.56E-02 | hypothetical protein |
|  | SCO4340 | 0.818 | 2.56E-02 | integrase |
|  | SCO3579 | 0.706 | 4.92E-02 | regulatory protein |
|  | SCO1784 | 0.676 | 4.89E-02 | hypothetical protein |
| Downregulated | SCO1116 | -0.663 | 3.84E-02 | hypothetical protein |
|  | SCO4294 | -0.724 | 4.80E-02 | hypothetical protein |
|  | SCOt06 | -0.817 | 4.59E-02 | tRNA-Val |
|  | SCO1523 | -0.868 | 1.01E-03 | pyridoxal biosynthesis lyase PdxS |
|  | SCO2146 | -0.889 | 9.85E-03 | aminotransferase |
|  | SCOt55 | -0.959 | 4.80E-02 | tRNA-Arg |

**Supplementary Table S7 Gene name of ACT biosynthetic gene clusters**

| Locus_tag | Gene |
| --- | --- |
| SCO5071 | SCBAC20F6.14c |
| SCO5072 | SCBAC20F6.15 |
| SCO5073 | SCBAC20F6.16 |
| SCO5074 | SCBAC20F6.17 |
| SCO5075 | ORF4 |
| SCO5076 | actVA1 |
| SCO5077 | actVA2 |
| SCO5078 | actVA3 |
| SCO5079 | actVA4 |
| SCO5080 | actVA5 |
| SCO5081 | actVA6 |
| SCO5082 | actII-1 |
| SCO5083 | actII-2 |
| SCO5084 | actII-3 |
| SCO5085 | actII-4 |
| SCO5086 | actIII |
| SCO5087 | actIORF1 |
| SCO5088 | actIORF2 |
| SCO5089 | actIORF3 |
| SCO5090 | actVII |
| SCO5091 | actIV |
| SCO5092 | actVB |

**Supplementary Table S8 List of DEGs in cAMP supplementation RNAseq of M1146**

0h-2h

|  | Locus_tag | log2 fold-change | FDR | Product |
| --- | --- | --- | --- | --- |
| Upregulated | SCO4225 | 4.268802 | 4.93E-46 | hypothetical protein |
|  | SCO7460 | 3.870598 | 3.11E-37 | lipoprotein |
|  | SCO7461 | 3.448306 | 1.80E-31 | hydrolase |
|  | SCO6682 | 3.397151 | 7.68E-30 | hypothetical protein |
|  | SCO1700 | 3.385493 | 6.03E-59 | hypothetical protein |
|  | SCO4425 | 3.286517 | 2.16E-26 | sigma-like protein |
|  | SCO1773 | 3.249582 | 4.44E-40 | L-alanine dehydrogenase |
|  | SCO7613 | 3.216213 | 4.51E-32 | hypothetical protein |
|  | SCO4224 | 3.122607 | 3.95E-35 | hypothetical protein |
|  | SCO1699 | 3.107479 | 7.63E-54 | transcriptional regulator |
|  | SCO0607 | 2.878871 | 1.46E-18 | lipoprotein |
|  | SCO7612 | 2.784227 | 1.48E-30 | hypothetical protein |
|  | SCO6729 | 2.757481 | 2.62E-26 | hypothetical protein |
|  | SCO1698 | 2.58548 | 9.71E-34 | hypothetical protein |
|  | SCO4924 | 2.575861 | 3.47E-15 | hypothetical protein |
|  | SCO4011 | 2.272175 | 4.92E-17 | hypothetical protein |
|  | SCO3900 | 2.13079 | 2.96E-11 | hypothetical protein |
|  | SCO0608 | 2.129755 | 1.26E-15 | regulatory protein |
|  | SCOt05 | 2.106527 | 1.16E-03 | tRNA-Val |
|  | SCO1550 | 2.100474 | 4.84E-07 | small membrane protein |
|  | SCO6728 | 2.02151 | 7.42E-03 | hypothetical protein |
|  | SCO2113 | 2.008261 | 9.64E-12 | bacterioferritin |
|  | SCOt40 | 1.918938 | 2.34E-05 | tRNA-Gly |
|  | SCO5436 | 1.878173 | 2.09E-20 | sodium:dicarboxylate symporter |
|  | SCO4683 | 1.858371 | 3.74E-18 | glutamate dehydrogenase |
|  | SCO4979 | 1.840807 | 2.49E-15 | phosphoenolpyruvate carboxykinase |
|  | SCO0609 | 1.826778 | 4.25E-10 | hypothetical protein |
|  | SCO6429 | 1.781823 | 1.09E-12 | hypothetical protein |
|  | SCO4635 | 1.761568 | 1.23E-05 | 50S ribosomal protein L33 |
|  | SCO6431 | 1.761275 | 1.35E-18 | peptide synthase |
|  | SCO6681 | 1.758479 | 3.47E-15 | Ser/Thr protein kinase |
|  | SCO0736 | 1.737275 | 2.57E-06 | hypothetical protein |
|  | SCOt59 | 1.718141 | 4.64E-08 | tRNA-Gln |
|  | SCO6434 | 1.689358 | 3.47E-15 | oxidoreductase |
|  | SCO6433 | 1.689342 | 7.44E-17 | hypothetical protein |
|  | SCO3097 | 1.673448 | 2.85E-14 | hypothetical protein |
|  | SCO4784 | 1.646582 | 2.09E-11 | hypothetical protein |
|  | SCO2100 | 1.627741 | 3.41E-08 | transcriptional regulator |
|  | SCO7511 | 1.600463 | 6.24E-10 | glyceraldehyde 3-phosphate dehydrogenase |
|  | SCO6436 | 1.579918 | 9.40E-14 | tRNA synthetase |
|  | SCO3924 | 1.543849 | 2.09E-06 | hypothetical protein |
|  | SCO5521 | 1.53667 | 9.93E-11 | hypothetical protein |
|  | SCO6435 | 1.517707 | 3.99E-03 | hypothetical protein |
|  | SCO5679 | 1.504608 | 1.62E-10 | aldehyde dehydrogenase |
|  | SCOt50 | 1.494434 | 3.04E-05 | tRNA-Met |
|  | SCO3111 | 1.454898 | 1.62E-10 | ABC transporter ATP-binding protein |
|  | SCOt51 | 1.451563 | 1.08E-03 | tRNA-Trp |
|  | SCO4492 | 1.42468 | 2.30E-07 | octaprenyl carboxylase |
|  | SCO3289 | 1.422715 | 5.18E-16 | large membrane protein |
|  | SCO3899 | 1.401718 | 4.64E-08 | hypothetical protein |
|  | SCO6432 | 1.39317 | 1.31E-13 | peptide synthase |
|  | SCO3194 | 1.39213 | 1.17E-07 | lipoprotein |
|  | SCO3356 | 1.384343 | 2.84E-05 | ECF sigma factor |
|  | SCO5783 | 1.381447 | 2.74E-05 | hypothetical protein |
|  | SCO3167 | 1.350914 | 6.60E-09 | TetR family transcriptional regulator |
|  | SCO6430 | 1.32058 | 1.36E-08 | hypothetical protein |
|  | SCO3327 | 1.313757 | 5.20E-04 | hypothetical protein |
|  | SCOt58 | 1.302183 | 1.51E-03 | tRNA-Glu |
|  | SCO3290 | 1.30145 | 1.11E-11 | hypothetical protein |
|  | SCOt48 | 1.300241 | 1.83E-03 | tRNA-Tyr |
|  | SCO2917 | 1.294454 | 4.79E-07 | nicotinate phosphoribosyltransferase |
|  | SCO3288 | 1.289339 | 2.19E-11 | hypothetical protein |
|  | SCO3328 | 1.284374 | 5.68E-04 | hypothetical protein |
|  | SCO4371 | 1.279278 | 4.86E-07 | hypothetical protein |
|  | SCO4908 | 1.273806 | 1.05E-08 | RNA polymerase sigma factor |
|  | SCO1563 | 1.268036 | 1.72E-04 | acetyltransferase |
|  | SCO3105 | 1.25621 | 1.48E-08 | hypothetical protein |
|  | SCO5676 | 1.252771 | 1.11E-11 | 4-aminobutyrate aminotransferase |
|  | SCO3714 | 1.249471 | 4.71E-05 | transposase |
|  | SCO3342 | 1.234117 | 1.45E-07 | glycine-rich hypothetical protein |
|  | SCO5862 | 1.227405 | 6.65E-07 | two-component regulator CutR |
|  | SCO3862 | 1.226094 | 3.76E-03 | hypothetical protein |
|  | SCO5157 | 1.221153 | 2.87E-06 | metal-transport protein |
|  | SCO5610 | 1.216817 | 1.64E-03 | hypothetical protein |
|  | SCO1174 | 1.213397 | 2.22E-06 | aldehyde dehydrogenase |
|  | SCO5782 | 1.205692 | 1.99E-05 | transmembrane transport protein |
|  | SCO2066 | 1.181556 | 3.99E-04 | hypothetical protein |
|  | SCO4002 | 1.180065 | 8.26E-08 | hypothetical protein |
|  | SCO0703 | 1.176685 | 6.00E-04 | regulator |
|  | SCO0955 | 1.167057 | 1.49E-04 | hypothetical protein |
|  | SCO2457 | 1.164014 | 1.15E-05 | lipoprotein |
|  | SCOt11 | 1.162557 | 1.32E-03 | tRNA-Met |
|  | SCO5797 | 1.158786 | 9.73E-05 | hypothetical protein |
|  | SCO2546 | 1.153891 | 5.89E-07 | adenosine deaminase |
|  | SCO3977 | 1.153317 | 2.26E-05 | protease |
|  | SCO2947 | 1.152469 | 6.81E-03 | hypothetical protein |
|  | SCO3613 | 1.151831 | 3.27E-04 | RNA polymerase sigma factor |
|  | SCO1645 | 1.14839 | 6.94E-03 | hypothetical protein |
|  | SCO6683 | 1.147794 | 3.49E-07 | ABC transporter ATP-binding protein |
|  | SCO5047 | 1.147497 | 1.62E-04 | fructose 1%2C6-bisphosphatase II |
|  | SCO1643 | 1.141117 | 4.39E-03 | 20S proteasome alpha-subunit |
|  | SCO1793 | 1.124369 | 1.57E-04 | hypothetical protein |
|  | SCOr09 | 1.114252 | 6.94E-03 | 16S ribosomal RNA |
|  | SCO5169 | 1.098247 | 1.48E-02 | ATP-binding protein |
|  | SCO4710 | 1.098211 | 1.05E-02 | 50S ribosomal protein L29 |
|  | SCO4923 | 1.094574 | 7.13E-04 | hypothetical protein |
|  | SCO1710 | 1.094024 | 1.36E-07 | integral membrane transport protein |
|  | SCO5724 | 1.093316 | 2.91E-03 | hypothetical protein |
|  | SCO4187 | 1.091317 | 1.52E-04 | hypothetical protein |
|  | SCO0735 | 1.089292 | 7.21E-06 | oxidoreductase |
|  | SCO5230 | 1.086772 | 8.28E-03 | hypothetical protein |
|  | SCO4651 | 1.084175 | 6.08E-05 | lipoprotein |
|  | SCO4783 | 1.082797 | 9.79E-05 | hypothetical protein |
|  | SCO5393 | 1.072674 | 4.35E-04 | ABC transporter ATP-binding protein |
|  | SCOt56 | 1.071584 | 1.78E-02 | tRNA-Gln |
|  | SCO1839 | 1.070556 | 4.84E-03 | transcriptional regulator |
|  | SCO6091 | 1.069547 | 2.49E-06 | hypothetical protein |
|  | SCOs01 | 1.069146 | 1.20E-02 | ribonuclease P RNA |
|  | SCO6715 | 1.067552 | 3.19E-02 | transcriptional regulator |
|  | SCO3357 | 1.061488 | 6.10E-05 | hypothetical protein |
|  | SCO5660 | 1.053593 | 8.26E-08 | peptidase |
|  | SCO3110 | 1.052863 | 1.14E-05 | ABC transporter |
|  | SCO3034 | 1.049396 | 6.94E-03 | sporulation regulatory protein |
|  | SCO5464 | 1.047168 | 1.65E-03 | hypothetical protein |
|  | SCO4424 | 1.041991 | 2.94E-06 | hypothetical protein |
|  | SCO5148 | 1.040423 | 3.02E-04 | hypothetical protein |
|  | SCO1875 | 1.038561 | 9.83E-06 | penicillin binding protein |
|  | SCO2628 | 1.030828 | 4.98E-04 | amino acid permease |
|  | SCO4317 | 1.030174 | 2.73E-03 | hypothetical protein |
|  | SCO1562 | 1.02911 | 1.17E-04 | hypothetical protein |
|  | SCO7646 | 1.025533 | 2.18E-02 | hypothetical protein |
|  | SCO3396 | 1.023308 | 2.30E-06 | hypothetical protein |
|  | SCO5195 | 1.022157 | 9.77E-04 | hypothetical protein |
|  | SCO1965 | 1.018369 | 1.37E-03 | export associated protein |
|  | SCO1638 | 1.017089 | 1.58E-02 | peptidyl-prolyl cis-trans isomerase |
|  | SCO3870 | 1.010125 | 1.12E-02 | hypothetical protein |
|  | SCO4137 | 1.004392 | 5.12E-03 | hypothetical protein |
|  | SCO4903 | 1.004367 | 4.39E-05 | hypothetical protein |
|  | SCO5663 | 1.001972 | 8.40E-03 | hypothetical protein |
|  | SCO5863 | 1.000496 | 1.79E-04 | two-component sensor (kinase) |
|  | SCO1940 | 0.999305 | 3.01E-04 | hypothetical protein |
|  | SCO2648 | 0.995574 | 6.81E-03 | hypothetical protein |
|  | SCOt31 | 0.98968 | 1.33E-02 | tRNA-Leu |
|  | SCO1385 | 0.987642 | 4.17E-03 | hypothetical protein |
|  | SCO5537 | 0.987164 | 2.73E-03 | ATP/GTP binding protein |
|  | SCO1375 | 0.986699 | 6.01E-03 | hypothetical protein |
|  | SCO1548 | 0.986581 | 5.12E-03 | hypothetical protein |
|  | SCO2078 | 0.983954 | 9.25E-03 | hypothetical protein |
|  | SCO2505 | 0.977474 | 4.29E-06 | ABC-transporter metal-binding lipoprotein |
|  | SCO5664 | 0.976657 | 1.25E-03 | hypothetical protein |
|  | SCO5145 | 0.976222 | 3.39E-02 | hypothetical protein |
|  | SCO5570 | 0.97038 | 1.06E-02 | hypothetical protein |
|  | SCO4725 | 0.970265 | 1.11E-02 | translation initiation factor IF-1 |
|  | SCO5798 | 0.965272 | 1.24E-03 | hypothetical protein |
|  | SCO4223 | 0.962988 | 3.59E-08 | AraC family transcription regulator |
|  | SCO4037 | 0.959077 | 1.15E-02 | small membrane protein |
|  | SCO0209 | 0.954496 | 6.86E-04 | hypothetical protein |
|  | SCO6592 | 0.950267 | 6.51E-03 | hypothetical protein |
|  | SCO4088 | 0.949882 | 1.84E-02 | hypothetical protein |
|  | SCO3546 | 0.945176 | 5.38E-03 | hypothetical protein |
|  | SCO4847 | 0.944654 | 1.74E-03 | D-alanyl-D-alanine carboxypeptidase |
|  | SCO0462 | 0.940064 | 2.35E-03 | oxidoreductase |
|  | SCO6773 | 0.939216 | 5.47E-06 | peptidase |
|  | SCO1964 | 0.938612 | 3.58E-03 | export associated protein |
|  | SCO5720 | 0.933486 | 3.58E-03 | hypothetical protein |
|  | SCO2258 | 0.932825 | 3.01E-04 | ABC transporter |
|  | SCO3397 | 0.931783 | 1.00E-04 | integral membrane lysyl-tRNA synthetase |
|  | SCO5856 | 0.931701 | 4.55E-04 | hypothetical protein |
|  | SCO3974 | 0.931569 | 9.93E-04 | hypothetical protein |
|  | SCO1440 | 0.930911 | 1.61E-02 | 6%2C7-dimethyl-8-ribityllumazine synthase |
|  | SCO3909 | 0.930116 | 2.73E-02 | 50S ribosomal protein L9 |
|  | SCO4493 | 0.929633 | 3.84E-04 | transcription regulator AsnC |
|  | SCO1644 | 0.928834 | 1.07E-02 | 20S proteasome subunit beta |
|  | SCO1470 | 0.924989 | 6.95E-03 | hypothetical protein |
|  | SCO0268 | 0.924309 | 3.22E-02 | hypothetical protein |
|  | SCO2456 | 0.922756 | 5.39E-04 | hypothetical protein |
|  | SCO2918 | 0.918224 | 1.65E-03 | nicotinamidase |
|  | SCO5568 | 0.914919 | 3.92E-03 | phosphopantetheine adenylyltransferase |
|  | SCO1777 | 0.913662 | 1.00E-04 | hypothetical protein |
|  | SCO3713 | 0.913393 | 1.00E-02 | hypothetical protein |
|  | SCO5839 | 0.912824 | 7.73E-04 | hypothetical protein |
|  | SCO2899 | 0.912693 | 1.97E-02 | GroES family molecular chaperone |
|  | SCO4584 | 0.912051 | 1.28E-03 | hypothetical protein |
|  | SCO7322 | 0.911638 | 2.50E-02 | anti-sigma factor |
|  | SCO1941 | 0.911361 | 1.24E-04 | hypothetical protein |
|  | SCO3579 | 0.90764 | 2.51E-02 | regulatory protein |
|  | SCO5725 | 0.905451 | 1.02E-02 | hypothetical protein |
|  | SCO5358 | 0.904316 | 1.73E-05 | hypothetical protein |
|  | SCO4713 | 0.904183 | 3.41E-02 | 50S ribosomal protein L24 |
|  | SCO5147 | 0.90332 | 1.89E-04 | RNA polymerase sigma factor SigE |
|  | SCO6671 | 0.895753 | 3.99E-04 | hypothetical protein |
|  | SCO3146 | 0.89483 | 1.14E-03 | hypothetical protein |
|  | SCO6008 | 0.894318 | 8.02E-03 | transcriptional repressor protein |
|  | SCO1944 | 0.890875 | 3.02E-02 | preprotein translocase subunit SecG |
|  | SCO7042 | 0.890422 | 1.48E-02 | MarR family transcriptional regulator |
|  | SCO4524 | 0.888925 | 4.91E-03 | hypothetical protein |
|  | SCO3712 | 0.888754 | 4.40E-04 | hydrolase |
|  | SCO3911 | 0.887112 | 1.02E-04 | replicative DNA helicase |
|  | SCO2224 | 0.886775 | 1.12E-02 | hypothetical protein |
|  | SCO7393 | 0.884327 | 4.01E-05 | lipoprotein |
|  | SCO1602 | 0.883529 | 4.99E-03 | hypothetical protein |
|  | SCO5413 | 0.882814 | 9.49E-03 | MarR-transcriptional regulator |
|  | SCO2257 | 0.882145 | 3.01E-04 | ABC transporter ATP-binding protein |
|  | SCO5742 | 0.881002 | 1.85E-02 | hypothetical protein |
|  | SCO6225 | 0.878917 | 1.20E-03 | hypothetical protein |
|  | SCO1691 | 0.877157 | 5.13E-03 | TetR family transcriptional regulator |
|  | SCO4884 | 0.87409 | 2.16E-04 | lipoprotein |
|  | SCO1950 | 0.873415 | 1.33E-02 | hypothetical protein |
|  | SCO5545 | 0.87242 | 2.41E-02 | hypothetical protein |
|  | SCO2303 | 0.871461 | 2.10E-02 | hypothetical protein |
|  | SCO3880 | 0.870431 | 1.66E-02 | 50S ribosomal protein L34 |
|  | SCO1963 | 0.869756 | 4.97E-04 | integral membrane export protein |
|  | SCO7615 | 0.868608 | 7.25E-04 | DNA-binding protein |
|  | SCO3881 | 0.868508 | 2.53E-02 | ribonuclease P component |
|  | SCO4228 | 0.865243 | 8.18E-03 | phosphate transport system regulator |
|  | SCO6437 | 0.862492 | 1.01E-07 | hypothetical protein |
|  | SCO5197 | 0.86179 | 3.81E-03 | hypothetical protein |
|  | SCO1319 | 0.859962 | 1.77E-02 | hypothetical protein |
|  | SCO4652 | 0.85892 | 3.88E-02 | 50S ribosomal protein L10 |
|  | SCO1543 | 0.857554 | 6.46E-03 | hypothetical protein |
|  | SCO2668 | 0.852316 | 1.14E-03 | hypothetical protein |
|  | SCO3761 | 0.852184 | 9.38E-03 | hypothetical protein |
|  | SCO4716 | 0.84966 | 2.83E-02 | 30S ribosomal protein S8 |
|  | SCO2097 | 0.848708 | 4.81E-02 | hypothetical protein |
|  | SCO1749 | 0.846225 | 1.33E-02 | hypothetical protein |
|  | SCO6760 | 0.845884 | 1.33E-02 | phytoene synthase |
|  | SCO4791 | 0.845867 | 1.62E-04 | two-component system sensor kinase |
|  | SCO6268 | 0.843992 | 1.35E-02 | histidine kinase |
|  | SCO3906 | 0.842258 | 3.32E-02 | 30S ribosomal protein S6 |
|  | SCO4798 | 0.84104 | 3.09E-03 | peptidase |
|  | SCO2579 | 0.840003 | 7.08E-03 | nicotinic acid mononucleotide adenylyltransferase |
|  | SCO2458 | 0.83923 | 8.09E-03 | hypothetical protein |
|  | SCO4736 | 0.839035 | 4.68E-03 | phosphoglucosamine mutase |
|  | SCO2041 | 0.837669 | 2.15E-02 | hypothetical protein |
|  | SCO4895 | 0.83701 | 6.42E-03 | RNA polymerase factor sigma-70 |
|  | SCO1600 | 0.836894 | 4.63E-02 | translation initiation factor IF-3 |
|  | SCO2167 | 0.835168 | 9.38E-03 | hypothetical protein |
|  | SCO5481 | 0.833656 | 4.93E-04 | hypothetical protein |
|  | SCO1320 | 0.833041 | 1.47E-03 | hypothetical protein |
|  | SCO1082 | 0.832364 | 2.67E-02 | electron transfer flavoprotein subunit beta |
|  | SCO4226 | 0.832336 | 1.24E-03 | hypothetical protein |
|  | SCO3086 | 0.830791 | 2.41E-04 | lipoprotein |
|  | SCO5733 | 0.828333 | 3.00E-02 | hypothetical protein |
|  | SCO2663 | 0.827567 | 2.21E-03 | hypothetical protein |
|  | SCO5151 | 0.824827 | 1.46E-02 | hypothetical protein |
|  | SCO2807 | 0.824511 | 2.50E-03 | hypothetical protein |
|  | SCO6722 | 0.823809 | 1.33E-02 | regulator |
|  | SCO5112 | 0.822456 | 4.79E-03 | ABC transporter integral membrane protein BldKA |
|  | SCO0596 | 0.820126 | 1.54E-02 | DNA-binding protein |
|  | SCO4885 | 0.818309 | 3.40E-03 | lipoprotein |
|  | SCO3884 | 0.817313 | 3.15E-02 | hypothetical protein |
|  | SCO5869 | 0.816639 | 3.02E-02 | hypothetical protein |
|  | SCO1114 | 0.815028 | 1.84E-02 | uracil-DNA glycosylase |
|  | SCO6493 | 0.810639 | 3.62E-02 | hypothetical protein |
|  | SCO0323 | 0.810305 | 2.31E-04 | hypothetical protein |
|  | SCO5244 | 0.809426 | 2.01E-02 | anti-sigma factor |
|  | SCO1514 | 0.80905 | 3.06E-02 | adenine phosphoribosyltransferase |
|  | SCO4227 | 0.808777 | 2.77E-02 | hypothetical protein |
|  | SCO2949 | 0.807902 | 5.49E-03 | UDP-N-acetylglucosamine 1-carboxyvinyltransferase |
|  | SCOs02 | 0.806989 | 2.11E-02 | Note=ssrA gene coding for tmRNA%2C 397 bp%3B similar to SW:MT10SARNA (EMBL:X60301) Mycobacterium tuberculosis gene for 10Sa RNA%2C 606 bp%3B |
|  | SCO5492 | 0.80573 | 1.68E-02 | short chain dehydrogenase |
|  | SCO3575 | 0.802873 | 2.22E-02 | hypothetical protein |
|  | SCO3202 | 0.802629 | 9.76E-04 | RNA polymerase principal sigma factor |
|  | SCO4091 | 0.802285 | 4.04E-02 | DNA-binding protein |
|  | SCO6224 | 0.800557 | 9.13E-03 | hypothetical protein |
|  | SCO4506 | 0.798919 | 7.79E-03 | hypothetical protein |
|  | SCO4684 | 0.797934 | 1.45E-02 | cold shock protein |
|  | SCO3180 | 0.796522 | 3.67E-02 | molybdenum cofactor biosynthesis protein MoaC |
|  | SCO5459 | 0.796441 | 3.14E-03 | enoyl-CoA hydratase |
|  | SCO7536 | 0.79458 | 8.43E-03 | hypothetical protein |
|  | SCO4686 | 0.794226 | 4.14E-02 | hypothetical protein |
|  | SCO2582 | 0.792697 | 1.11E-02 | hypothetical protein |
|  | SCO3811 | 0.79216 | 1.32E-04 | D-alanyl-D-alanine carboxypeptidase |
|  | SCO3074 | 0.79205 | 2.91E-02 | hypothetical protein |
|  | SCO3958 | 0.791937 | 3.98E-03 | ABC transporter ATP-binding protein |
|  | SCO4108 | 0.789926 | 1.12E-02 | peptidase |
|  | SCO6103 | 0.789871 | 2.45E-03 | acetyltransferase |
|  | SCO3612 | 0.789081 | 4.37E-04 | hypothetical protein |
|  | SCO3378 | 0.788588 | 9.65E-03 | small membrane protein |
|  | SCO5458 | 0.78727 | 2.94E-03 | lipoprotein |
|  | SCO2366 | 0.785655 | 1.43E-02 | hypothetical protein |
|  | SCO4737 | 0.783847 | 4.84E-03 | hypothetical protein |
|  | SCO0797 | 0.783698 | 6.40E-03 | hypothetical protein |
|  | SCO6618 | 0.782271 | 2.74E-02 | hypothetical protein |
|  | SCO4043 | 0.77911 | 3.49E-02 | hypothetical protein |
|  | SCO5562 | 0.779005 | 2.21E-02 | thiamine monophosphate kinase |
|  | SCO3925 | 0.776073 | 9.12E-05 | transcriptional regulator |
|  | SCO5732 | 0.774482 | 2.22E-02 | hypothetical protein |
|  | SCO5561 | 0.771992 | 1.13E-02 | hypothetical protein |
|  | SCO4585 | 0.770874 | 4.86E-03 | ABC transporter ATP-binding protein |
|  | SCO3572 | 0.770844 | 1.80E-02 | hydrolase |
|  | SCO1755 | 0.769301 | 8.58E-03 | hypothetical protein |
|  | SCOr13, SCOr14, SCOr15 | 0.769171 | 3.67E-02 | 16S ribosomal RNA, 23S ribosomal RNA, 5S ribosomal RNA |
|  | SCO5392 | 0.768937 | 3.31E-02 | ABC transporter |
|  | SCO1464 | 0.767658 | 2.73E-02 | ribulose-phosphate 3-epimerase |
|  | SCO2089 | 0.767515 | 1.15E-02 | UDP-N-acetylmuramoylalanyl-D-glutamate--2%2C6-diaminopimelate ligase |
|  | SCO2339 | 0.767409 | 2.73E-02 | hypothetical protein |
|  | SCO3898 | 0.767162 | 2.41E-04 | hypothetical protein |
|  | SCO6031 | 0.765233 | 3.61E-02 | uroporphyrinogen decarboxylase |
|  | SCO6218 | 0.76459 | 1.36E-03 | phosphatase |
|  | SCO6621 | 0.764556 | 8.33E-04 | hypothetical protein |
|  | SCO5754 | 0.764002 | 3.44E-03 | hypothetical protein |
|  | SCO3844 | 0.76214 | 2.09E-02 | hypothetical protein |
|  | SCO5243 | 0.762039 | 1.65E-02 | RNA polymerase sigma factor |
|  | SCO1542 | 0.761953 | 6.94E-03 | hypothetical protein |
|  | SCO5265 | 0.759483 | 1.84E-03 | hypothetical protein |
|  | SCO1384 | 0.75913 | 3.64E-02 | hypothetical protein |
|  | SCO5044 | 0.758843 | 1.60E-02 | fumarate hydratase class I |
|  | SCOt24 | 0.758215 | 3.28E-02 | tRNA-Leu |
|  | SCO1720 | 0.757788 | 6.94E-03 | ABC-transporter transmembrane protein |
|  | SCO6589 | 0.756978 | 4.31E-02 | elongation factor G |
|  | SCO0695 | 0.756843 | 1.98E-02 | hypothetical protein |
|  | SCO2939 | 0.755898 | 9.87E-04 | hypothetical protein |
|  | SCO2162 | 0.754582 | 1.77E-02 | quinolinate synthetase |
|  | SCO3887 | 0.75232 | 4.31E-02 | partitioning or sporulation protein |
|  | SCO5784 | 0.751329 | 9.92E-04 | two-component sensor |
|  | SCO2067 | 0.74797 | 6.10E-03 | hypothetical protein |
|  | SCO1639 | 0.746069 | 2.53E-02 | peptidyl-prolyl cis-trans isomerase |
|  | SCO5504 | 0.745064 | 1.65E-02 | hypothetical protein |
|  | SCO3913 | 0.744599 | 2.18E-02 | hypothetical protein |
|  | SCO6139 | 0.744332 | 1.65E-02 | sensor kinase |
|  | SCO3358 | 0.744079 | 2.03E-02 | two-component system response regulator |
|  | SCO5143 | 0.7438 | 2.20E-02 | DNA-3-methyladenine glycosylase I |
|  | SCO3045 | 0.741533 | 1.70E-03 | hypothetical protein |
|  | SCO5556 | 0.741431 | 9.49E-03 | histone-like DNA binding protein |
|  | SCO1391 | 0.741358 | 3.23E-02 | phosphoenolpyruvate-protein phosphotransferase |
|  | SCO1395 | 0.74105 | 3.03E-02 | mutT-like protein |
|  | SCO5875 | 0.741037 | 2.10E-02 | potassium uptake protein |
|  | SCO1540 | 0.74032 | 4.52E-02 | hypothetical protein |
|  | SCO5833 | 0.738778 | 2.36E-02 | hypothetical protein |
|  | SCO4005 | 0.737886 | 3.48E-03 | RNA polymerase sigma factor |
|  | SCO1462 | 0.736643 | 3.12E-02 | hypothetical protein |
|  | SCOr4, SCOr5, SCOr6 | 0.736391 | 4.96E-02 | 5S ribosomal RNA, 23S ribosomal RNA, 16S ribosomal RNA |
|  | SCO4289 | 0.735768 | 2.49E-03 | hypothetical protein |
|  | SCO2942 | 0.733219 | 4.47E-02 | oxidoreductase |
|  | SCO5115 | 0.731374 | 2.36E-02 | ABC transporter intracellular ATPase subunit BldKD |
|  | SCO5285 | 0.731053 | 7.52E-06 | ATP-dependent protease |
|  | SCO7204 | 0.730683 | 3.56E-02 | hypothetical protein |
|  | SCO7477 | 0.730588 | 3.83E-03 | hypothetical protein |
|  | SCO2769 | 0.727915 | 7.50E-04 | acetolactate synthase |
|  | SCO0730 | 0.727823 | 4.14E-02 | hypothetical protein |
|  | SCO2903 | 0.72778 | 1.17E-02 | hypothetical protein |
|  | SCO2838 | 0.724099 | 6.94E-03 | endoglucanase |
|  | SCO6537 | 0.723524 | 3.41E-02 | DNA-binding protein |
|  | SCO4012 | 0.722328 | 2.54E-03 | hypothetical protein |
|  | SCO3145 | 0.721804 | 9.21E-03 | hypothetical protein |
|  | SCO1748 | 0.720058 | 9.20E-03 | expression regulator |
|  | SCO2730 | 0.719972 | 3.67E-02 | regulator |
|  | SCOr16, SCOr17, SCOr18 | 0.719485 | 4.78E-02 | 16S ribosomal RNA, 23S ribosomal RNA, 5S ribosomal RNA |
|  | SCO7284 | 0.716372 | 4.80E-02 | ribonuclease H |
|  | SCO3044 | 0.715274 | 3.56E-03 | hypothetical protein |
|  | SCO1566 | 0.714814 | 3.67E-02 | acyltransferase |
|  | SCO3124 | 0.713471 | 5.00E-02 | 50S ribosomal protein L25/general stress protein Ctc |
|  | SCO1526 | 0.71296 | 4.63E-02 | lipid A biosynthesis lauroyl acyltransferase |
|  | SCO4240 | 0.708745 | 1.11E-02 | ABC transporter ATP-binding protein |
|  | SCO4439 | 0.707224 | 2.40E-02 | D-alanyl-D-alanine carboxypeptidase |
|  | SCO3580 | 0.707067 | 3.04E-02 | transpeptidase |
|  | SCO5793 | 0.705827 | 3.77E-02 | diaminopimelate epimerase |
|  | SCO4790 | 0.705302 | 2.65E-04 | hypothetical protein |
|  | SCO5806 | 0.704034 | 3.49E-02 | hypothetical protein |
|  | SCO4980 | 0.701185 | 1.39E-03 | hypothetical protein |
|  | SCO4529 | 0.700468 | 2.65E-02 | hypothetical protein |
|  | SCO2019 | 0.700381 | 4.52E-02 | chorismate mutase |
|  | SCO3760 | 0.698188 | 5.20E-04 | hypothetical protein |
|  | SCO1806 | 0.697974 | 2.76E-02 | ABC transporter ATP-binding protein |
|  | SCO0763 | 0.697789 | 1.58E-02 | oxidoreductase |
|  | SCO6721 | 0.695891 | 5.90E-04 | hypothetical protein |
|  | SCO2999 | 0.694715 | 1.94E-02 | hypothetical protein |
|  | SCO5032 | 0.689686 | 4.61E-02 | alkyl hydroperoxide reductase |
|  | SCO2080 | 0.688 | 3.56E-02 | hypothetical protein |
|  | SCO5114 | 0.687703 | 1.43E-02 | ABC transporter integral membrane protein BldKC |
|  | SCO2069 | 0.687277 | 2.68E-02 | hypothetical protein |
|  | SCO5785 | 0.686697 | 8.96E-03 | two-component regulator |
|  | SCO1791 | 0.684795 | 3.56E-02 | hypothetical protein |
|  | SCO1767 | 0.684539 | 4.99E-02 | DNA hydrolase |
|  | SCO1165 | 0.68443 | 1.14E-02 | hypothetical protein |
|  | SCO5146 | 0.684293 | 4.43E-02 | methyltransferase |
|  | SCO2837 | 0.683878 | 2.57E-02 | hypothetical protein |
|  | SCO6723 | 0.68298 | 8.19E-03 | oxidoreductase |
|  | SCO2664 | 0.681599 | 1.73E-02 | sugar-binding protein |
|  | SCO5538 | 0.680709 | 4.05E-02 | hypothetical protein |
|  | SCO1138 | 0.678671 | 3.86E-03 | hypothetical protein |
|  | SCO3061 | 0.677343 | 3.41E-02 | hypothetical protein |
|  | SCO6528 | 0.675289 | 1.18E-05 | integral membrane transport protein |
|  | SCO4089 | 0.673192 | 1.54E-02 | valine dehydrogenase |
|  | SCO2116 | 0.672998 | 2.11E-02 | hypothetical protein |
|  | SCO5196 | 0.668086 | 9.65E-03 | hypothetical protein |
|  | SCO4330 | 0.667817 | 2.56E-02 | hypothetical protein |
|  | SCO0922 | 0.663784 | 2.15E-02 | succinate dehydrogenase/fumarate reductase iron-sulfur subunit |
|  | SCO3820 | 0.661626 | 3.67E-02 | Ser/Thr protein kinase |
|  | SCO5857 | 0.661571 | 1.73E-02 | FAD-dependent oxidoreductase |
|  | SCO5363 | 0.661338 | 3.77E-02 | hypothetical protein |
|  | SCO3959 | 0.661029 | 1.14E-03 | hypothetical protein |
|  | SCO2595 | 0.659063 | 3.77E-02 | GTPase ObgE |
|  | SCO2110 | 0.653661 | 3.94E-02 | Ser/Thr protein kinase |
|  | SCO3621 | 0.652895 | 1.04E-02 | serine-threonine protein kinase |
|  | SCO5555 | 0.652463 | 4.31E-02 | hypothetical protein |
|  | SCO4724 | 0.652236 | 3.91E-02 | methionine aminopeptidase |
|  | SCO7327 | 0.651555 | 7.82E-03 | two-component system sensory histidine kinase |
|  | SCO1870 | 0.650559 | 5.49E-03 | hypothetical protein |
|  | SCO6989 | 0.646528 | 1.37E-02 | hypothetical protein |
|  | SCO2061 | 0.646415 | 4.18E-02 | ABC transport ATP-binding subunit |
|  | SCO4590 | 0.644814 | 8.43E-03 | hypothetical protein |
|  | SCO2892 | 0.644652 | 1.14E-02 | hypothetical protein |
|  | SCO2088 | 0.644208 | 4.18E-02 | UDP-N-acetylmuramoylalanyl-D-glutamyl-2%2C6-diaminopimelate- D-alanyl-alanyl ligase |
|  | SCO1780 | 0.643117 | 3.12E-02 | DNA repair protein |
|  | SCO2183 | 0.642182 | 4.47E-02 | 2-oxoacid dehydrogenase subunit E1 |
|  | SCO5734 | 0.641942 | 4.73E-02 | ATP/GTP binding protein membrane protein |
|  | SCO2897 | 0.641449 | 4.28E-03 | penicillin-binding protein |
|  | SCO2316 | 0.641246 | 3.48E-02 | hypothetical protein |
|  | SCO6140 | 0.640827 | 1.36E-02 | two-component system response regulator |
|  | SCO7614 | 0.638683 | 4.24E-02 | regulatory protein |
|  | SCO4440 | 0.638565 | 3.09E-02 | hypothetical protein |
|  | SCO6973 | 0.638429 | 7.77E-03 | hypothetical protein |
|  | SCO3800 | 0.637902 | 2.17E-05 | acyl-CoA dehydrogenase |
|  | SCO1201 | 0.637473 | 2.91E-03 | reductase |
|  | SCO1349 | 0.635447 | 2.09E-02 | hypothetical protein |
|  | SCO0726 | 0.633556 | 3.66E-02 | oxidoreductase |
|  | SCO4184 | 0.633208 | 1.32E-02 | aerial mycelium formation protein |
|  | SCO2725 | 0.632198 | 4.76E-02 | lipoprotein |
|  | SCO3704 | 0.631695 | 1.35E-02 | substrate-binding transport protein |
|  | SCO6695 | 0.631259 | 4.33E-02 | hypothetical protein |
|  | SCO4869 | 0.630928 | 3.25E-02 | methylmalonyl CoA mutase |
|  | SCO5332 | 0.630834 | 4.45E-02 | hypothetical protein |
|  | SCO5386 | 0.629717 | 2.73E-02 | anti-sigma factor antagonist |
|  | SCO7060 | 0.62959 | 2.29E-02 | hypothetical protein |
|  | SCO5870 | 0.629205 | 3.28E-03 | ABC transporter ATP-binding protein |
|  | SCO2455 | 0.628562 | 3.45E-03 | spermidine synthase |
|  | SCO1653 | 0.628409 | 3.02E-02 | hypothetical protein |
|  | SCO2960 | 0.628334 | 1.94E-02 | hypothetical protein |
|  | SCO5575 | 0.628142 | 1.14E-02 | transmembrane protein |
|  | SCO5191 | 0.627099 | 2.95E-02 | hypothetical protein |
|  | SCO3400 | 0.626773 | 2.63E-02 | dihydroneopterin aldolase |
|  | SCO3893 | 0.626055 | 4.31E-02 | hypothetical protein |
|  | SCO0851 | 0.625533 | 4.38E-02 | PfkB-family carbohydrate kinase |
|  | SCO3098 | 0.625328 | 4.73E-02 | hypothetical protein |
|  | SCO0729 | 0.622684 | 3.97E-02 | hypothetical protein |
|  | SCO0289 | 0.622001 | 1.25E-02 | transcriptional regulator |
|  | SCO7233 | 0.619518 | 3.33E-02 | hypothetical protein |
|  | SCO5721 | 0.618625 | 4.76E-02 | hypothetical protein |
|  | SCO1659 | 0.615798 | 2.79E-02 | glycerol uptake facilitator protein |
|  | SCO5563 | 0.615682 | 4.28E-02 | phosphomethylpyrimidine kinase |
|  | SCO2318 | 0.614147 | 6.94E-03 | glycosyl transferase |
|  | SCO3879 | 0.612435 | 3.56E-02 | chromosomal replication initiation protein |
|  | SCO7596 | 0.612429 | 1.17E-02 | integral membrane transport protein |
|  | SCO5659 | 0.612215 | 9.02E-03 | hypothetical protein |
|  | SCO4236 | 0.610931 | 4.02E-02 | 23S rRNA (guanosine(2251)-2'-O)-methyltransferase RlmB |
|  | SCO4186 | 0.609441 | 6.94E-03 | hypothetical protein |
|  | SCO0230 | 0.607541 | 2.99E-02 | TetR family transcriptional regulator |
|  | SCO2193 | 0.605496 | 2.55E-02 | lipoyltransferase |
|  | SCO4779 | 0.605423 | 1.12E-02 | Ser/Thr protein kinase |
|  | SCO4172 | 0.603996 | 1.26E-02 | hypothetical protein |
|  | SCO7298 | 0.601723 | 3.17E-02 | thioredoxin reductase |
|  | SCO4438 | 0.599367 | 4.78E-02 | hypothetical protein |
|  | SCO5415 | 0.597117 | 4.93E-04 | isobutyryl-CoA mutase A |
|  | SCO2320 | 0.596123 | 2.09E-02 | hypothetical protein |
|  | SCO1996 | 0.595897 | 4.99E-02 | dephospho-CoA kinase |
|  | SCO4472 | 0.595154 | 4.05E-02 | hypothetical protein |
|  | SCO3956 | 0.594372 | 1.52E-02 | ABC transporter ATP-binding protein |
|  | SCO5817 | 0.593763 | 1.75E-02 | DNA hydrolase |
|  | SCO5781 | 0.592544 | 2.75E-02 | hypothetical protein |
|  | SCO3048 | 0.586902 | 2.71E-02 | hypothetical protein |
|  | SCO2839 | 0.582331 | 1.46E-02 | lipoprotein |
|  | SCO5140 | 0.582023 | 2.23E-02 | hypothetical protein |
|  | SCO1827 | 0.581415 | 1.33E-02 | DNA polymerase III subunit epsilon |
|  | SCO3401 | 0.580976 | 1.32E-02 | 2-amino-4-hydroxy-6-hydroxymethyldihydropteridine diphosphokinase |
|  | SCO3359 | 0.578621 | 3.21E-02 | sensory histidine kinase contains hydrophobic membrane spanning regions |
|  | SCO5138 | 0.578177 | 1.73E-02 | hypothetical protein |
|  | SCO7285 | 0.575444 | 2.40E-02 | hydroxylase |
|  | SCO4531 | 0.575152 | 4.24E-02 | septum determining protein |
|  | SCO0508 | 0.575025 | 2.40E-02 | tetR family transcriptional regulator |
|  | SCO1863 | 0.574382 | 2.69E-02 | hypothetical protein |
|  | SCO3885 | 0.57349 | 2.73E-02 | 16S rRNA methyltransferase GidB |
|  | SCO5748 | 0.571361 | 3.70E-02 | sensory histidine kinase |
|  | SCO1779 | 0.56867 | 4.67E-02 | hypothetical protein |
|  | SCO4528 | 0.567608 | 1.06E-02 | hypothetical protein |
|  | SCO0954 | 0.566239 | 1.99E-03 | acetyltransferase |
|  | SCO2516 | 0.564364 | 9.46E-03 | hypothetical protein |
|  | SCO6776 | 0.559787 | 4.47E-02 | hypothetical protein |
|  | SCO2057 | 0.556164 | 4.53E-02 | hypothetical protein |
|  | SCO1169 | 0.556089 | 1.65E-02 | xylose isomerase |
|  | SCO3895 | 0.554187 | 4.68E-02 | hypothetical protein |
|  | SCO4042 | 0.553803 | 2.74E-02 | hypothetical protein |
|  | SCO0072 | 0.553664 | 1.55E-02 | hypothetical protein |
|  | SCO5657 | 0.553269 | 4.99E-02 | aldehyde dehydrogenase |
|  | SCO1323 | 0.552695 | 3.56E-02 | hypothetical protein |
|  | SCO4075 | 0.552116 | 8.58E-03 | ABC transporter ATP-binding protein |
|  | SCO4904 | 0.550783 | 3.41E-02 | hypothetical protein |
|  | SCO0563 | 0.550338 | 3.30E-02 | hypothetical protein |
|  | SCO7439 | 0.550257 | 4.52E-02 | hypothetical protein |
|  | SCO2862 | 0.54968 | 4.73E-02 | hypothetical protein |
|  | SCO5179 | 0.5483 | 2.67E-02 | peptidase |
|  | SCO4566 | 0.547036 | 2.77E-02 | NADH dehydrogenase subunit E |
|  | SCO5257 | 0.54675 | 4.67E-02 | methyltransferase |
|  | SCO6005 | 0.546526 | 1.27E-02 | lipoprotein |
|  | SCO7824 | 0.546343 | 2.23E-02 | TetR family transcriptional regulator |
|  | SCO4118 | 0.544253 | 3.46E-02 | TetR family transcriptional regulator |
|  | SCO5190 | 0.544078 | 1.92E-02 | DNA-binding protein |
|  | SCO2863 | 0.541633 | 1.94E-02 | helicase |
|  | SCO1658 | 0.540508 | 1.54E-02 | glycerol operon regulatory protein |
|  | SCO1789 | 0.539349 | 1.78E-02 | hypothetical protein |
|  | SCO2494 | 0.53886 | 3.83E-03 | pyruvate phosphate dikinase |
|  | SCO4533 | 0.538416 | 3.16E-02 | hypothetical protein |
|  | SCO4782 | 0.536946 | 4.99E-02 | hypothetical protein |
|  | SCO6821 | 0.536057 | 1.77E-02 | hypothetical protein |
|  | SCO3106 | 0.533851 | 4.67E-02 | lipoprotein |
|  | SCO6045 | 0.53098 | 1.55E-02 | hypothetical protein |
|  | SCO3896 | 0.530016 | 4.61E-02 | RNA nucleotidyltransferase |
|  | SCO6020 | 0.528746 | 1.27E-02 | transcriptional regulator |
|  | SCO1211 | 0.528207 | 4.52E-02 | polypeptide deformylase |
|  | SCO2770 | 0.528084 | 2.73E-02 | agmatinase |
|  | SCO2726 | 0.523222 | 2.73E-02 | methylmalonic acid semialdehyde dehydrogenase |
|  | SCO2092 | 0.522862 | 4.38E-02 | S-adenosyl-methyltransferase MraW |
|  | SCO3868 | 0.522739 | 3.71E-02 | hypothetical protein |
|  | SCO1409 | 0.518042 | 4.14E-02 | hypothetical protein |
|  | SCO2665 | 0.517726 | 3.12E-02 | hypothetical protein |
|  | SCO5487 | 0.517406 | 3.40E-02 | hypothetical protein |
|  | SCO2004 | 0.51548 | 3.02E-02 | formate dehydrogenase |
|  | SCO4669 | 0.514442 | 1.86E-02 | hypothetical protein |
|  | SCO3046 | 0.513286 | 1.80E-02 | hypothetical protein |
|  | SCO3166 | 0.513221 | 2.43E-04 | membrane transport protein |
|  | SCO1709 | 0.513152 | 1.33E-02 | integral membrane transport protein |
|  | SCO1871 | 0.510908 | 4.76E-02 | aldehyde dehydrogenase |
|  | SCO0974 | 0.506307 | 1.84E-02 | hypothetical protein |
|  | SCO4752 | 0.505775 | 4.89E-02 | DNA-binding/iron metalloprotein/AP endonuclease |
|  | SCO3050 | 0.505671 | 4.52E-02 | hypothetical protein |
|  | SCO3286 | 0.504949 | 4.63E-02 | hypothetical protein |
|  | SCO2771 | 0.501474 | 3.44E-02 | hypothetical protein |
| Downregulated | SCO4945 | -0.50065 | 1.73E-02 | dehydrogenase |
|  | SCO0854 | -0.50358 | 4.79E-02 | hypothetical protein |
|  | SCO6530 | -0.50802 | 4.51E-02 | hypothetical protein |
|  | SCO6393 | -0.51625 | 3.14E-03 | transposase |
|  | SCO2254 | -0.51724 | 3.81E-02 | transmembrane efflux protein |
|  | SCO5009 | -0.51822 | 1.55E-02 | secretory protein |
|  | SCO3540 | -0.51875 | 4.52E-02 | proteinase |
|  | SCO3765 | -0.52721 | 3.57E-02 | hypothetical protein |
|  | SCO4689 | -0.52828 | 3.03E-02 | hypothetical protein |
|  | SCO2014 | -0.53111 | 4.68E-02 | pyruvate kinase |
|  | SCO1575 | -0.53572 | 1.65E-02 | thiamine biosynthesis lipoprotein |
|  | SCO6394 | -0.54269 | 1.26E-02 | IS element ATP binding protein |
|  | SCO0783 | -0.54322 | 1.44E-02 | tetracycline resistance protein |
|  | SCO0976 | -0.54657 | 1.46E-02 | hypothetical protein |
|  | SCO0199 | -0.54788 | 4.51E-02 | alcohol dehydrogenase |
|  | SCO2527 | -0.54857 | 1.29E-02 | hypothetical protein |
|  | SCO0491 | -0.54971 | 2.95E-02 | ABC transporter |
|  | SCO2649 | -0.55065 | 3.09E-04 | 4-alpha-glucanotransferase |
|  | SCO3383 | -0.55584 | 2.23E-02 | pantoate--beta-alanine ligase |
|  | SCO5774 | -0.55893 | 2.43E-02 | glutamate permease |
|  | SCO0588 | -0.56313 | 1.27E-02 | sensor kinase |
|  | SCO6054 | -0.56884 | 4.52E-02 | transmembrane transport protein |
|  | SCO6073 | -0.57098 | 1.15E-02 | cyclase |
|  | SCO4242 | -0.57131 | 2.73E-02 | hypothetical protein |
|  | SCO6149 | -0.57164 | 1.05E-02 | ribosome-associated GTPase |
|  | SCO6222 | -0.5745 | 6.94E-03 | aminotransferase AlaT |
|  | SCO2011 | -0.57813 | 3.31E-02 | ABC transporter ATP-binding protein |
|  | SCO2026 | -0.57886 | 4.63E-02 | glutamate synthase |
|  | SCO7703 | -0.5801 | 1.26E-02 | integral membrane transport protein |
|  | SCO4831 | -0.58382 | 4.64E-04 | glycine/betaine ABC transporter integral membrane protein |
|  | SCO7517 | -0.58793 | 3.94E-02 | hypothetical protein |
|  | SCO4679 | -0.58866 | 1.77E-02 | hypothetical protein |
|  | SCO5451 | -0.59653 | 5.97E-03 | ABC transporter |
|  | SCO1067 | -0.59732 | 3.64E-02 | integral membrane transport protein |
|  | SCO5929 | -0.59835 | 1.25E-02 | oxidoreductase |
|  | SCO7721 | -0.60276 | 1.55E-03 | hypothetical protein |
|  | SCO4829 | -0.6042 | 5.51E-03 | oxidoreductase |
|  | SCO0213 | -0.60429 | 8.96E-03 | nitrate/nitrite transporter |
|  | SCO3814 | -0.61431 | 3.59E-02 | DNA-binding protein |
|  | SCO1786 | -0.61461 | 2.67E-02 | iron-siderophore uptake system transmembrane protein |
|  | SCO4501 | -0.61889 | 3.56E-02 | 3-ketoacyl-ACP reductase |
|  | SCO1396 | -0.62296 | 1.84E-03 | D-alanyl-D-alanine dipeptidase |
|  | SCO1864 | -0.62721 | 1.02E-02 | acetyltransferase |
|  | SCO5207 | -0.62762 | 3.17E-02 | hypothetical protein |
|  | SCO3953 | -0.63121 | 1.24E-03 | RNA 2'-phosphotransferase-like protein |
|  | SCO0216 | -0.63447 | 5.31E-03 | nitrate reductase subunit alpha NarG2 |
|  | SCO0273 | -0.63863 | 3.15E-02 | substrate binding protein |
|  | SCO0149 | -0.63904 | 1.81E-02 | hypothetical protein |
|  | SCO0488 | -0.64234 | 9.86E-03 | hydrolase |
|  | SCO2765 | -0.64282 | 6.94E-03 | hypothetical protein |
|  | SCO6529 | -0.64396 | 1.33E-02 | ATP/GTP binding protein |
|  | SCO5250 | -0.64423 | 3.08E-03 | polyprenyl synthetase |
|  | SCO2883 | -0.64527 | 1.06E-02 | cytochrome P450 |
|  | SCO2009 | -0.64747 | 1.12E-02 | branched-chain amino acid ABC transporter permease |
|  | SCO3406 | -0.65624 | 1.33E-02 | hypothetical protein |
|  | SCO6797 | -0.65678 | 9.49E-03 | ATP/GTP binding protein |
|  | SCO1617 | -0.65794 | 2.97E-02 | hypothetical protein |
|  | SCO5006 | -0.67196 | 6.52E-04 | septum site-determining protein |
|  | SCO6660 | -0.67796 | 1.15E-02 | hypothetical protein |
|  | SCO2655 | -0.68313 | 1.36E-02 | hypothetical protein |
|  | SCO2781 | -0.69042 | 5.33E-03 | hypothetical protein |
|  | SCO2528 | -0.69248 | 1.77E-02 | 2-isopropylmalate synthase |
|  | SCO0585 | -0.69669 | 3.44E-03 | ATP/GTP binding protein |
|  | SCO0985 | -0.69672 | 2.12E-04 | 5-methyltetrahydropteroyltriglutamate/homocysteine S-methyltransferase |
|  | SCO7699 | -0.69726 | 2.12E-02 | nucleotide-binding protein |
|  | SCO0494 | -0.70008 | 3.78E-02 | iron-siderophore binding lipoprotein |
|  | SCO1985 | -0.7009 | 8.89E-03 | hypothetical protein |
|  | SCO1558 | -0.70252 | 1.44E-03 | ABC transporter permease |
|  | SCO6055 | -0.70493 | 1.25E-02 | carbonic anhydrase |
|  | SCO4245 | -0.70627 | 1.96E-05 | hypothetical protein |
|  | SCO1567 | -0.70836 | 2.22E-04 | transmembrane-transport protein |
|  | SCO6096 | -0.70968 | 3.61E-02 | lipoprotein |
|  | SCO5404 | -0.71315 | 1.51E-05 | two-component sensor kinase |
|  | SCO1374 | -0.71572 | 3.17E-05 | hypothetical protein |
|  | SCO4854 | -0.71981 | 9.50E-03 | hypothetical protein |
|  | SCO5221 | -0.72878 | 3.11E-05 | polypeptide deformylase |
|  | SCO5174 | -0.72888 | 7.13E-04 | transferase |
|  | SCO5292 | -0.72924 | 1.19E-04 | ATP/GTP-binding protein |
|  | SCO0993 | -0.73088 | 3.21E-04 | hypothetical protein |
|  | SCO0220 | -0.73972 | 6.94E-03 | hypothetical protein |
|  | SCO0217 | -0.74035 | 9.76E-04 | nitrate reductase subunit beta NarH2 |
|  | SCO6102 | -0.74873 | 2.60E-02 | nitrite/sulfite reductase |
|  | SCO0490 | -0.75207 | 3.99E-04 | esterase |
|  | SCO5389 | -0.75374 | 7.07E-03 | hypothetical protein |
|  | SCO1487 | -0.75495 | 3.60E-04 | aspartate carbamoyltransferase catalytic subunit |
|  | SCO3710 | -0.75602 | 5.86E-03 | large integral membrane protein |
|  | SCO2384 | -0.75661 | 8.96E-03 | hypothetical protein |
|  | SCO1134 | -0.7575 | 1.91E-03 | oxidoreductase%2C iron-sulfur binding subunit |
|  | SCO7016 | -0.76386 | 1.15E-04 | LacI family transcriptional regulator |
|  | SCO1132 | -0.76531 | 1.47E-06 | oxidoreductase |
|  | SCO2010 | -0.76792 | 5.70E-04 | branched-chain amino acid ABC transporter permease |
|  | SCO2716 | -0.77354 | 3.54E-02 | hypothetical protein |
|  | SCO5405 | -0.77465 | 1.14E-02 | transcriptional regulator |
|  | SCO0931 | -0.77472 | 7.01E-05 | proline-rich protein |
|  | SCO1968 | -0.77869 | 1.72E-03 | hydrolase |
|  | SCO4159 | -0.78042 | 9.49E-03 | transcriptional regulator |
|  | SCO1673 | -0.78087 | 7.90E-03 | hypothetical protein |
|  | SCO2108 | -0.78155 | 5.87E-03 | hypothetical protein |
|  | SCO0593 | -0.78345 | 6.16E-04 | hypothetical protein |
|  | SCO4348 | -0.78505 | 8.68E-04 | hypothetical protein |
|  | SCO1570 | -0.78958 | 4.59E-03 | argininosuccinate lyase |
|  | SCO4048 | -0.79543 | 1.12E-02 | hypothetical protein |
|  | SCO4007 | -0.79934 | 1.19E-06 | hypothetical protein |
|  | SCO7842 | -0.80224 | 6.36E-06 | transposase |
|  | SCO0005 | -0.80366 | 5.19E-06 | transposase |
|  | SCO1580 | -0.81532 | 6.94E-03 | N-acetyl-gamma-glutamyl-phosphate reductase |
|  | SCO4937 | -0.8192 | 3.13E-02 | hypothetical protein |
|  | SCO0492 | -0.81923 | 2.00E-07 | peptide synthetase |
|  | SCO3350 | -0.82767 | 8.03E-04 | hypothetical protein |
|  | SCO6094 | -0.83408 | 6.94E-03 | transport system integral membrane protein |
|  | SCO1559 | -0.83532 | 1.06E-02 | ABC transporter ATP-binding protein |
|  | SCO1105 | -0.84096 | 1.89E-07 | hypothetical protein |
|  | SCO2487 | -0.84696 | 4.52E-02 | nitrite reductase large subunit NirB |
|  | SCO4164 | -0.84721 | 2.44E-03 | thiosulfate sulfurtransferase |
|  | SCO2449 | -0.84764 | 1.15E-04 | hypothetical protein |
|  | SCO6099 | -0.84943 | 3.81E-03 | adenylyl-sulfate kinase |
|  | SCO1244 | -0.85097 | 1.24E-03 | biotin synthase |
|  | SCO1579 | -0.85187 | 6.01E-04 | bifunctional ornithine acetyltransferase/N-acetylglutamate synthase |
|  | SCO3764 | -0.85248 | 3.10E-08 | hypothetical protein |
|  | SCO7700 | -0.85546 | 1.29E-03 | cyclase |
|  | SCO7434 | -0.85837 | 2.86E-04 | lipoprotein |
|  | SCO4999 | -0.86083 | 1.36E-02 | hypothetical protein |
|  | SCO0774 | -0.87313 | 1.89E-07 | cytochrome P450 |
|  | SCO6100 | -0.87723 | 3.80E-03 | phosphoadenosine phosphosulfate reductase |
|  | SCO3928 | -0.87859 | 2.73E-03 | thiamine biosynthesis protein ThiC |
|  | SCO5175 | -0.88275 | 2.09E-06 | hypothetical protein |
|  | SCO0991 | -0.88614 | 1.65E-03 | hypothetical protein |
|  | SCO5249 | -0.88905 | 4.62E-05 | nucleotide-binding protein |
|  | SCO0888 | -0.89753 | 6.29E-04 | hypothetical protein |
|  | SCO0219 | -0.90091 | 2.63E-04 | nitrate reductase subunit delta NarI2 |
|  | SCO5640 | -0.90549 | 1.34E-02 | hypothetical protein |
|  | SCO7841 | -0.94719 | 2.28E-05 | ATP/GTP-binding protein |
|  | SCO1785 | -0.96052 | 8.48E-05 | iron-siderophore uptake system ATP-binding protein |
|  | SCO1866 | -0.96154 | 7.36E-04 | L-ectoine synthase |
|  | SCO2269 | -0.96949 | 9.86E-03 | hypothetical protein |
|  | SCO0631 | -0.97713 | 2.37E-02 | hypothetical protein |
|  | SCO0958 | -0.9788 | 1.61E-07 | hypothetical protein |
|  | SCO7709 | -0.97882 | 2.65E-06 | MarR family transcriptional regulator |
|  | SCO0006 | -0.9907 | 5.51E-06 | ATP/GTP-binding protein |
|  | SCO2213 | -0.99254 | 3.91E-04 | regulatory protein |
|  | SCO7014 | -1.0074 | 4.66E-07 | LacI family transcriptional regulator |
|  | SCO6663 | -1.00908 | 1.49E-06 | transketolase |
|  | SCO2272 | -1.00975 | 1.08E-03 | binding-protein-dependent transport lipoprotein |
|  | SCO0995 | -1.01011 | 1.23E-03 | methyltransferase |
|  | SCO1867 | -1.01121 | 1.90E-04 | hydroxylase |
|  | SCO1947 | -1.01961 | 1.17E-04 | glyceraldehyde-3-phosphate dehydrogenase |
|  | SCO5535 | -1.03382 | 3.84E-04 | carboxyl transferase |
|  | SCO6097 | -1.03994 | 1.18E-04 | sulfate adenylyltransferase subunit 1 |
|  | SCO2780 | -1.04514 | 8.24E-06 | hypothetical protein |
|  | SCO2911 | -1.05744 | 3.61E-07 | hypothetical protein |
|  | SCO2256 | -1.06568 | 2.11E-11 | 3-methyl-2-oxobutanoate hydroxymethyltransferase |
|  | SCO6098 | -1.06687 | 6.33E-05 | sulfate adenylyltransferase subunit 2 |
|  | SCO5772 | -1.08675 | 2.13E-05 | hypothetical protein |
|  | SCO2016 | -1.0882 | 8.81E-10 | monooxygenase |
|  | SCO5977 | -1.09302 | 4.27E-14 | amino acid permease |
|  | SCO2912 | -1.09334 | 6.35E-05 | hypothetical protein |
|  | SCO5976 | -1.09387 | 8.40E-07 | ornithine carbamoyltransferase |
|  | SCO7398 | -1.09592 | 1.83E-12 | iron-hydroxamate transporter permease subunit |
|  | SCO1133 | -1.09704 | 5.15E-09 | oxidoreductase%2C molybdopterin binding subunit |
|  | SCO6426 | -1.10794 | 2.07E-02 | hypothetical protein |
|  | SCO0587 | -1.10904 | 3.96E-06 | hypothetical protein |
|  | SCO5578 | -1.11206 | 1.15E-06 | sugar transporter |
|  | SCO7153 | -1.12814 | 1.80E-07 | sugar transporter |
|  | SCO1086 | -1.12848 | 4.87E-05 | hypothetical protein |
|  | SCO3253 | -1.13698 | 4.03E-02 | SpdD protein |
|  | SCO0586 | -1.13735 | 8.88E-07 | hypothetical protein |
|  | SCO0994 | -1.14354 | 1.93E-11 | hypothetical protein |
|  | SCO0799 | -1.15637 | 2.32E-03 | hypothetical protein |
|  | SCO3711 | -1.15979 | 4.43E-07 | small membrane protein |
|  | SCO6056 | -1.20128 | 4.44E-09 | hypothetical protein |
|  | SCO0218 | -1.20791 | 3.80E-08 | nitrate reductase subunit delta NarJ2 |
|  | SCO0681 | -1.26722 | 2.62E-06 | ferredoxin/ferredoxin-NADP reductase |
|  | SCO4337 | -1.32105 | 6.46E-10 | integral membrane efflux protein |
|  | SCO5177 | -1.33871 | 1.17E-09 | hypothetical protein |
|  | SCO4347 | -1.35506 | 2.37E-20 | hypothetical protein |
|  | SCO0459 | -1.44625 | 4.38E-09 | hypothetical protein |
|  | SCO0499 | -1.46622 | 2.71E-07 | formyltransferase |
|  | SCO0992 | -1.48106 | 8.82E-18 | cysteine synthase |
|  | SCO0073 | -1.51153 | 8.48E-13 | hypothetical protein |
|  | SCO1294 | -1.53573 | 2.74E-05 | cystathionine gamma-synthase |
|  | SCO5176 | -1.60894 | 1.09E-15 | reductase |
|  | SCO2958 | -1.72813 | 2.00E-11 | bifunctional uroporphyrinogen-III synthetase/response regulator domain-containing protein |
|  | SCO7400 | -1.72889 | 8.39E-13 | ABC transporter ATP-binding protein |
|  | SCO2383 | -1.74162 | 3.99E-17 | hypothetical protein |
|  | SCO2198 | -1.81798 | 1.89E-13 | glutamine synthetase |
|  | SCO1293 | -1.83038 | 7.38E-17 | hypothetical protein |
|  | SCO2471 | -1.83141 | 7.44E-08 | hypothetical protein |
|  | SCO7399 | -1.89778 | 5.56E-16 | binding-protein-dependent transport lipoprotein |
|  | SCO2784 | -1.95187 | 3.34E-04 | aceytltranferase |
|  | SCO2782 | -1.9557 | 1.88E-11 | pyridoxal-dependent decarboxylase |
|  | SCO2785 | -2.04246 | 3.47E-15 | hypothetical protein |
|  | SCO0498 | -2.18168 | 4.51E-32 | peptide monooxygenase |
|  | SCO2271 | -2.24949 | 1.86E-20 | hypothetical protein |
|  | SCO2783 | -2.26307 | 1.90E-25 | monooxygenase |
|  | SCO2270 | -2.46119 | 1.92E-29 | hypothetical protein |
|  | SCO2211 | -3.73809 | 9.64E-80 | hypothetical protein |
|  | SCO7598 | -3.86577 | 3.93E-03 | hypothetical protein |
|  | SCO5585 | -4.13687 | 1.90E-59 | PII uridylyl-transferase |
|  | SCO5584 | -4.36288 | 7.98E-56 | nitrogen regulatory protein P-II |
|  | SCO5583 | -5.36105 | 2.11E-208 | ammonium transporter |
|  | SCO2210 | -6.04839 | 2.99E-03 | glutamine synthetase |

0h-1h

|  | Locus_tag | Log2 fold-change | FDR | Product |
| --- | --- | --- | --- | --- |
| Upregulated | SCO2113 | 2.929 | 9.42E-26 | bacterioferritin |
|  | SCO3299 | 2.553 | 6.80E-39 | hypothetical protein |
|  | SCO1905 | 2.482 | 1.56E-60 | hypothetical protein |
|  | SCO4979 | 2.407 | 1.94E-26 | phosphoenolpyruvate carboxykinase |
|  | SCO1700 | 2.362 | 1.11E-28 | hypothetical protein |
|  | SCO2146 | 2.347 | 1.08E-29 | aminotransferase |
|  | SCO4784 | 2.267 | 2.53E-22 | hypothetical protein |
|  | SCO3285 | 2.184 | 7.64E-51 | large glycine/alanine rich protein |
|  | SCO1699 | 2.146 | 1.21E-25 | transcriptional regulator |
|  | SCO5973 | 2.113 | 1.32E-41 | phosphatase |
|  | SCO5972 | 2.084 | 7.81E-37 | 3' terminal RNA ribose 2'-O-methyltransferase Hen1 |
|  | SCO5191 | 2.080 | 1.06E-20 | hypothetical protein |
|  | SCO3825 | 2.068 | 1.22E-34 | ABC-transporter transmembrane protein |
|  | SCO3286 | 2.036 | 3.96E-26 | hypothetical protein |
|  | SCO3824 | 2.006 | 4.43E-32 | ABC transporter ATP-binding protein |
|  | SCO1698 | 2.003 | 2.92E-20 | hypothetical protein |
|  | SCO6529 | 1.932 | 1.23E-22 | ATP/GTP binding protein |
|  | SCO0783 | 1.924 | 1.40E-29 | tetracycline resistance protein |
|  | SCO0795 | 1.894 | 4.38E-27 | hypothetical protein |
|  | SCO5190 | 1.867 | 1.65E-24 | DNA-binding protein |
|  | SCOt59 | 1.857 | 1.50E-09 | tRNA-Gln |
|  | SCO4225 | 1.822 | 2.34E-08 | hypothetical protein |
|  | SCO2145 | 1.733 | 1.55E-13 | glycerate kinase |
|  | SCO1174 | 1.730 | 5.89E-13 | aldehyde dehydrogenase |
|  | SCO5796 | 1.703 | 9.45E-11 | hypothetical protein |
|  | SCO4783 | 1.687 | 2.13E-11 | hypothetical protein |
|  | SCO1086 | 1.665 | 6.37E-11 | hypothetical protein |
|  | SCO1413 | 1.647 | 5.01E-21 | hypothetical protein |
|  | SCO0922 | 1.636 | 3.64E-12 | succinate dehydrogenase/fumarate reductase iron-sulfur subunit |
|  | SCO6682 | 1.618 | 1.04E-06 | hypothetical protein |
|  | SCO1963 | 1.578 | 6.30E-13 | integral membrane export protein |
|  | SCO6530 | 1.578 | 1.39E-16 | hypothetical protein |
|  | SCO0909 | 1.564 | 7.97E-11 | hypothetical protein |
|  | SCO7511 | 1.558 | 1.87E-09 | glyceraldehyde 3-phosphate dehydrogenase |
|  | SCO6512 | 1.534 | 2.36E-16 | ABC transporter ATP-binding protein |
|  | SCO1340 | 1.531 | 1.42E-11 | hypothetical protein |
|  | SCO0924 | 1.520 | 8.74E-07 | cytochrome B subunit |
|  | SCO7460 | 1.509 | 1.22E-05 | lipoprotein |
|  | SCO5521 | 1.479 | 4.94E-10 | hypothetical protein |
|  | SCO6429 | 1.472 | 1.05E-08 | hypothetical protein |
|  | SCO3287 | 1.470 | 3.67E-05 | serine/arginine rich protein |
|  | SCO7461 | 1.461 | 1.35E-05 | hydrolase |
|  | SCO5451 | 1.457 | 1.39E-16 | ABC transporter |
|  | SCO0106 | 1.446 | 1.24E-09 | insertion element transposase |
|  | SCO0408 | 1.437 | 2.41E-11 | methyltransferase |
|  | SCO1575 | 1.416 | 5.05E-16 | thiamine biosynthesis lipoprotein |
|  | SCO0908 | 1.405 | 3.68E-11 | hypothetical protein |
|  | SCO6729 | 1.403 | 8.74E-07 | hypothetical protein |
|  | SCO4683 | 1.383 | 4.02E-10 | glutamate dehydrogenase |
|  | SCO2027 | 1.376 | 2.60E-14 | hypothetical protein |
|  | SCO0854 | 1.373 | 3.02E-12 | hypothetical protein |
|  | SCO2100 | 1.351 | 1.02E-05 | transcriptional regulator |
|  | SCO6681 | 1.347 | 6.03E-09 | Ser/Thr protein kinase |
|  | SCO4224 | 1.339 | 1.80E-06 | hypothetical protein |
|  | SCO4963 | 1.321 | 2.80E-09 | ABC transporter ATP-binding protein |
|  | SCO2591 | 1.300 | 4.77E-06 | hypothetical protein |
|  | SCO7653 | 1.298 | 1.80E-05 | hypothetical protein |
|  | SCO5861 | 1.297 | 1.66E-09 | hypothetical protein |
|  | SCO1558 | 1.290 | 8.16E-12 | ABC transporter permease |
|  | SCO1729 | 1.266 | 3.40E-11 | hypothetical protein |
|  | SCO4765 | 1.263 | 5.99E-12 | hypothetical protein |
|  | SCO6447 | 1.260 | 8.30E-09 | NAD(P)H oxidoreductase |
|  | SCO7612 | 1.240 | 5.79E-06 | hypothetical protein |
|  | SCO1341 | 1.216 | 2.90E-08 | lipoprotein |
|  | SCO7731 | 1.215 | 6.48E-11 | hypothetical protein |
|  | SCO3006 | 1.208 | 3.18E-13 | acetyltransferase |
|  | SCO4293 | 1.207 | 3.03E-09 | threonine synthase |
|  | SCO6435 | 1.191 | 1.22E-05 | hypothetical protein |
|  | SCO3631 | 1.188 | 3.53E-07 | hypothetical protein |
|  | SCO6295 | 1.186 | 5.02E-15 | ABC transporter ATP-binding protein |
|  | SCO5157 | 1.185 | 6.78E-06 | metal-transport protein |
|  | SCO1580 | 1.179 | 1.54E-05 | N-acetyl-gamma-glutamyl-phosphate reductase |
|  | SCO4924 | 1.179 | 2.86E-03 | hypothetical protein |
|  | SCO1586 | 1.176 | 4.66E-08 | hypothetical protein |
|  | SCO1585 | 1.171 | 3.10E-05 | hypothetical protein |
|  | SCO6517 | 1.169 | 1.27E-08 | uvrA-like protein |
|  | SCO4964 | 1.168 | 1.60E-11 | integral membrane transport protein |
|  | SCO0607 | 1.168 | 3.38E-03 | lipoprotein |
|  | SCO1578 | 1.167 | 4.73E-05 | acetylglutamate kinase |
|  | SCO1576 | 1.164 | 1.99E-04 | arginine repressor |
|  | SCO6434 | 1.163 | 3.27E-07 | oxidoreductase |
|  | SCO0187 | 1.155 | 7.68E-04 | phytoene synthase |
|  | SCO7507 | 1.149 | 2.98E-08 | dioxygenase |
|  | SCO4264 | 1.149 | 2.40E-15 | aminoglycoside phosphotransferase |
|  | SCO1988 | 1.149 | 3.84E-07 | hypothetical protein |
|  | SCO1577 | 1.147 | 1.24E-04 | acetylornithine aminotransferase |
|  | SCO2379 | 1.142 | 2.85E-08 | acetyltransferase |
|  | SCO7606 | 1.141 | 4.68E-08 | amino acid binding protein |
|  | SCO0906 | 1.140 | 3.16E-05 | hypothetical protein |
|  | SCO2248 | 1.137 | 8.12E-08 | hypothetical protein |
|  | SCO1175 | 1.136 | 1.88E-04 | hypothetical protein |
|  | SCO1084 | 1.134 | 2.34E-07 | thioredoxin |
|  | SCO2257 | 1.126 | 1.01E-06 | ABC transporter ATP-binding protein |
|  | SCO2247 | 1.118 | 2.40E-08 | hypothetical protein |
|  | SCO7613 | 1.116 | 6.03E-04 | hypothetical protein |
|  | SCO0186 | 1.113 | 4.06E-04 | phytoene dehydrogenase |
|  | SCO1579 | 1.112 | 1.68E-06 | bifunctional ornithine acetyltransferase/N-acetylglutamate synthase |
|  | SCO1986 | 1.107 | 1.61E-04 | hypothetical protein |
|  | SCO1545 | 1.106 | 2.97E-05 | acetyltransferase |
|  | SCO1773 | 1.102 | 1.22E-04 | L-alanine dehydrogenase |
|  | SCO1147 | 1.099 | 3.92E-14 | ABC transporter transmembrane subunit |
|  | SCO7806 | 1.096 | 4.95E-05 | DNA-binding protein |
|  | SCO0608 | 1.093 | 3.17E-04 | regulatory protein |
|  | SCO1562 | 1.090 | 3.03E-05 | hypothetical protein |
|  | SCO3953 | 1.081 | 1.60E-11 | RNA 2'-phosphotransferase-like protein |
|  | SCO2117 | 1.075 | 1.57E-10 | anthranilate synthase |
|  | SCO5147 | 1.073 | 3.91E-06 | RNA polymerase sigma factor SigE |
|  | SCO5189 | 1.067 | 2.59E-04 | hypothetical protein |
|  | SCO6433 | 1.066 | 1.01E-06 | hypothetical protein |
|  | SCO3366 | 1.058 | 4.56E-05 | exporter |
|  | SCO6431 | 1.051 | 1.12E-06 | peptide synthase |
|  | SCO4908 | 1.050 | 5.68E-06 | RNA polymerase sigma factor |
|  | SCO7478 | 1.044 | 1.33E-06 | phosphotransferase |
|  | SCO5047 | 1.041 | 9.84E-04 | fructose 1%2C6-bisphosphatase II |
|  | SCO1816 | 1.041 | 5.72E-09 | hypothetical protein |
|  | SCO1731 | 1.037 | 2.06E-08 | hypothetical protein |
|  | SCO6464 | 1.036 | 1.08E-05 | SIR2 family transcriptional regulator |
|  | SCO0907 | 1.035 | 7.14E-07 | dehydrogenase |
|  | SCO4055 | 1.027 | 6.66E-11 | alcohol dehydrogenase |
|  | SCO4186 | 1.023 | 1.99E-07 | hypothetical protein |
|  | SCO7721 | 1.021 | 2.15E-10 | hypothetical protein |
|  | SCO0409 | 1.020 | 1.79E-05 | spore-associated protein |
|  | SCO7652 | 1.019 | 7.73E-04 | acetyltransferase |
|  | SCO3562 | 1.009 | 4.62E-13 | integral membrane transport protein |
|  | SCO6437 | 1.005 | 1.06E-10 | hypothetical protein |
|  | SCO7447 | 1.001 | 3.93E-05 | acetyltranferase |
|  | SCO4278 | 0.998 | 1.48E-06 | peptidyl-tRNA hydrolase domain-containing protein |
|  | SCO5974 | 0.998 | 6.28E-03 | integral membrane ion exchanger |
|  | SCO6436 | 0.996 | 1.59E-05 | tRNA synthetase |
|  | SCO1148 | 0.988 | 3.48E-11 | ABC transporter |
|  | SCO0955 | 0.983 | 2.62E-03 | hypothetical protein |
|  | SCO3606 | 0.983 | 1.24E-04 | regulator |
|  | SCO2755 | 0.979 | 1.12E-05 | acetyltransferase |
|  | SCO5450 | 0.972 | 4.27E-12 | ABC transporter |
|  | SCO5676 | 0.966 | 5.10E-07 | 4-aminobutyrate aminotransferase |
|  | SCO5449 | 0.965 | 5.17E-08 | ABC transporter |
|  | SCO2463 | 0.965 | 9.55E-05 | ABC transporter |
|  | SCO1405 | 0.962 | 5.76E-07 | HSP90 family protein |
|  | SCO1624 | 0.953 | 1.24E-04 | acetyltransferase |
|  | SCO4564 | 0.948 | 1.77E-05 | NADH dehydrogenase subunit C |
|  | SCO2373 | 0.947 | 8.42E-04 | tetracenomycin C efflux protein |
|  | SCO6952 | 0.946 | 2.83E-05 | hypothetical protein |
|  | SCO5044 | 0.943 | 1.86E-03 | fumarate hydratase class I |
|  | SCO7265 | 0.939 | 2.45E-04 | hypothetical protein |
|  | SCO5024 | 0.936 | 1.44E-04 | oxidoreductase |
|  | SCO0685 | 0.933 | 5.47E-04 | hypothetical protein |
|  | SCOt11 | 0.921 | 2.54E-02 | tRNA-Met |
|  | SCO2183 | 0.921 | 1.90E-03 | 2-oxoacid dehydrogenase subunit E1 |
|  | SCO6617 | 0.920 | 5.46E-03 | hypothetical protein |
|  | SCO2876 | 0.916 | 1.76E-06 | acetyltransferase |
|  | SCO0796 | 0.914 | 1.70E-06 | hypothetical protein |
|  | SCO5473 | 0.906 | 4.35E-04 | ATP/GTP binding protein |
|  | SCO4226 | 0.904 | 3.45E-04 | hypothetical protein |
|  | SCO4054 | 0.901 | 9.30E-03 | hypothetical protein |
|  | SCO3167 | 0.901 | 4.53E-04 | TetR family transcriptional regulator |
|  | SCO1647 | 0.891 | 5.25E-05 | hypothetical protein |
|  | SCO7710 | 0.887 | 2.40E-06 | phosphotransferase |
|  | SCO3602 | 0.880 | 3.29E-08 | transmembrane transport protein |
|  | SCO2258 | 0.879 | 8.61E-04 | ABC transporter |
|  | SCO3360 | 0.877 | 2.03E-06 | hypothetical protein |
|  | SCO7366 | 0.877 | 3.87E-03 | hypothetical protein |
|  | SCO1563 | 0.873 | 3.00E-02 | acetyltransferase |
|  | SCO3917 | 0.872 | 2.25E-05 | hypothetical protein |
|  | SCO6008 | 0.870 | 1.44E-02 | transcriptional repressor protein |
|  | SCO7651 | 0.868 | 1.24E-04 | TetR family transcriptional regulator |
|  | SCO1406 | 0.868 | 2.66E-04 | hypothetical protein |
|  | SCO7205 | 0.868 | 6.12E-05 | hydrolase |
|  | SCO2496 | 0.862 | 8.12E-04 | hypothetical protein |
|  | SCO5107 | 0.860 | 5.40E-04 | succinate dehydrogenase flavoprotein subunit |
|  | SCO0921 | 0.860 | 9.10E-05 | hypothetical protein |
|  | SCO3097 | 0.859 | 7.09E-04 | hypothetical protein |
|  | SCO5783 | 0.859 | 3.44E-02 | hypothetical protein |
|  | SCO0181 | 0.851 | 7.52E-06 | hypothetical protein |
|  | SCO3607 | 0.850 | 5.93E-03 | hypothetical protein |
|  | SCO5784 | 0.849 | 1.22E-04 | two-component sensor |
|  | SCO0381 | 0.847 | 4.88E-03 | glycosyl transferase |
|  | SCO4572 | 0.843 | 2.92E-02 | NADH dehydrogenase subunit K |
|  | SCO5148 | 0.842 | 7.78E-03 | hypothetical protein |
|  | SCO0684 | 0.841 | 1.51E-02 | hypothetical protein |
|  | SCO0382 | 0.841 | 2.27E-03 | UDP-glucose/GDP-mannose dehydrogenase |
|  | SCO1048 | 0.837 | 1.51E-02 | hypothetical protein |
|  | SCO3788 | 0.832 | 1.51E-02 | hypothetical protein |
|  | SCO1987 | 0.830 | 9.04E-03 | hypothetical protein |
|  | SCO0177 | 0.827 | 1.77E-04 | hypothetical protein |
|  | SCO2999 | 0.826 | 4.88E-03 | hypothetical protein |
|  | SCO7036 | 0.825 | 1.26E-02 | argininosuccinate synthase |
|  | SCO3111 | 0.823 | 1.69E-03 | ABC transporter ATP-binding protein |
|  | SCO1559 | 0.819 | 1.79E-02 | ABC transporter ATP-binding protein |
|  | SCO5999 | 0.818 | 3.25E-03 | aconitate hydratase |
|  | SCO2917 | 0.818 | 6.03E-03 | nicotinate phosphoribosyltransferase |
|  | SCO2372 | 0.814 | 2.04E-02 | small hydrophobic protein |
|  | SCO6432 | 0.813 | 1.01E-04 | peptide synthase |
|  | SCO5326 | 0.812 | 2.79E-03 | hypothetical protein |
|  | SCO3711 | 0.807 | 9.43E-04 | small membrane protein |
|  | SCO6446 | 0.807 | 1.51E-05 | hypothetical protein |
|  | SCO6228 | 0.805 | 1.40E-03 | hypothetical protein |
|  | SCO1404 | 0.805 | 9.53E-04 | hypothetical protein |
|  | SCO4311 | 0.804 | 5.80E-05 | hypothetical protein |
|  | SCO3977 | 0.803 | 1.00E-02 | protease |
|  | SCO4011 | 0.802 | 2.92E-02 | hypothetical protein |
|  | SCO5679 | 0.802 | 4.01E-03 | aldehyde dehydrogenase |
|  | SCO3710 | 0.801 | 3.54E-03 | large integral membrane protein |
|  | SCO4440 | 0.795 | 5.89E-03 | hypothetical protein |
|  | SCO1989 | 0.794 | 6.76E-03 | aminopeptidase |
|  | SCO1570 | 0.793 | 5.50E-03 | argininosuccinate lyase |
|  | SCO1765 | 0.792 | 3.22E-03 | hypothetical protein |
|  | SCO0174 | 0.792 | 2.26E-04 | DNA-binding protein |
|  | SCO1904 | 0.788 | 1.36E-02 | transcriptional regulator |
|  | SCO6009 | 0.785 | 2.40E-02 | solute-binding protein |
|  | SCO1403 | 0.785 | 1.61E-02 | hypothetical protein |
|  | SCO4903 | 0.778 | 3.87E-03 | hypothetical protein |
|  | SCO5976 | 0.777 | 1.44E-03 | ornithine carbamoyltransferase |
|  | SCO3105 | 0.775 | 2.13E-03 | hypothetical protein |
|  | SCO4222 | 0.774 | 4.05E-04 | hypothetical protein |
|  | SCO0166 | 0.768 | 8.12E-04 | regulator |
|  | SCO4980 | 0.767 | 3.33E-04 | hypothetical protein |
|  | SCO1902 | 0.767 | 4.79E-02 | hypothetical protein |
|  | SCO1085 | 0.764 | 1.51E-03 | acyltransferase |
|  | SCO1569 | 0.764 | 1.91E-02 | oxidoreductase |
|  | SCO4565 | 0.759 | 2.25E-05 | NADH dehydrogenase subunit D |
|  | SCO6683 | 0.759 | 2.84E-03 | ABC transporter ATP-binding protein |
|  | SCO2770 | 0.758 | 5.56E-04 | agmatinase |
|  | SCO2464 | 0.755 | 2.38E-06 | ABC transporter |
|  | SCO1640 | 0.755 | 3.00E-02 | hypothetical protein |
|  | SCO3656 | 0.750 | 1.71E-05 | hypothetical protein |
|  | SCO4676 | 0.746 | 1.66E-03 | hypothetical protein |
|  | SCO5660 | 0.743 | 5.29E-04 | peptidase |
|  | SCO5025 | 0.740 | 8.56E-04 | transcriptional regulator |
|  | SCO1334 | 0.734 | 3.79E-02 | hypothetical protein |
|  | SCO4006 | 0.733 | 2.38E-06 | long-chain-fatty-acid--CoA ligase |
|  | SCO3277 | 0.731 | 1.29E-02 | phosphotransferase |
|  | SCO1083 | 0.724 | 1.08E-02 | flavin-dependent reductase |
|  | SCO1795 | 0.723 | 2.29E-03 | hypothetical protein |
|  | SCO0954 | 0.714 | 2.89E-05 | acetyltransferase |
|  | SCO6304 | 0.712 | 1.21E-03 | oxidoreductase |
|  | SCO4409 | 0.711 | 6.22E-03 | RNA polymerase sigma factor |
|  | SCO6821 | 0.709 | 6.80E-04 | hypothetical protein |
|  | SCO3940 | 0.702 | 3.33E-02 | transmembrane protein |
|  | SCO1322 | 0.698 | 1.49E-03 | hypothetical protein |
|  | SCO5332 | 0.696 | 3.66E-02 | hypothetical protein |
|  | SCO5863 | 0.694 | 2.83E-02 | two-component sensor (kinase) |
|  | SCO4563 | 0.694 | 2.15E-02 | NADH dehydrogenase subunit B |
|  | SCO4954 | 0.693 | 5.59E-03 | hypothetical protein |
|  | SCO5166 | 0.690 | 2.37E-02 | helicase |
|  | SCO3385 | 0.687 | 2.08E-03 | L-allo-threonine aldolase |
|  | SCO1656 | 0.685 | 8.68E-03 | hydrolase |
|  | SCO6805 | 0.684 | 4.14E-04 | integral membrane efflux protein |
|  | SCO5785 | 0.682 | 1.17E-02 | two-component regulator |
|  | SCO2404 | 0.681 | 3.93E-04 | sugar-binding receptor |
|  | SCO4092 | 0.677 | 5.98E-03 | ATP-dependent helicase |
|  | SCO3007 | 0.674 | 1.58E-04 | hypothetical protein |
|  | SCO4098 | 0.673 | 1.76E-03 | acetyltransferase |
|  | SCO1544 | 0.673 | 2.43E-02 | hypothetical protein |
|  | SCO5977 | 0.673 | 9.39E-06 | amino acid permease |
|  | SCO3284 | 0.669 | 1.87E-05 | hypothetical protein |
|  | SCO0208 | 0.668 | 4.55E-05 | pyruvate phosphate dikinase |
|  | SCO2862 | 0.664 | 1.69E-02 | hypothetical protein |
|  | SCO3165 | 0.662 | 9.09E-03 | hypothetical protein |
|  | SCO1307 | 0.662 | 1.21E-02 | hypothetical protein |
|  | SCO3601 | 0.661 | 1.95E-03 | hypothetical protein |
|  | SCO2625 | 0.661 | 4.62E-04 | hypothetical protein |
|  | SCO1927 | 0.661 | 2.57E-03 | AAC(3) family N-acetyltransferase |
|  | SCO4976 | 0.659 | 2.06E-04 | hypothetical protein |
|  | SCO2318 | 0.658 | 3.79E-03 | glycosyl transferase |
|  | SCO6011 | 0.654 | 4.89E-02 | ABC transporter |
|  | SCO1165 | 0.653 | 2.53E-02 | hypothetical protein |
|  | SCO3321 | 0.653 | 3.16E-02 | redoxin |
|  | SCO2527 | 0.653 | 1.38E-03 | hypothetical protein |
|  | SCO0107 | 0.652 | 4.02E-02 | aminoglycoside nucleotidyltransferase |
|  | SCO5436 | 0.650 | 1.34E-02 | sodium:dicarboxylate symporter |
|  | SCO1596 | 0.648 | 9.14E-03 | two-component sensor |
|  | SCO2343 | 0.647 | 9.14E-03 | acetyltransferase |
|  | SCO0597 | 0.644 | 1.43E-02 | hypothetical protein |
|  | SCO2524 | 0.642 | 2.61E-02 | hypothetical protein |
|  | SCO3056 | 0.641 | 2.50E-02 | hypothetical protein |
|  | SCO2309 | 0.640 | 4.54E-04 | transmembrane transport protein |
|  | SCO7197 | 0.640 | 5.40E-04 | amino acid ABC transporter permease |
|  | SCO0215 | 0.635 | 5.09E-04 | hypothetical protein |
|  | SCO0593 | 0.632 | 7.95E-03 | hypothetical protein |
|  | SCO5004 | 0.624 | 1.35E-02 | hypothetical protein |
|  | SCO6430 | 0.623 | 4.15E-02 | hypothetical protein |
|  | SCO1726 | 0.623 | 9.99E-04 | ATPase |
|  | SCO5222 | 0.621 | 1.56E-03 | lyase |
|  | SCO7722 | 0.619 | 1.35E-02 | hypothetical protein |
|  | SCO0784 | 0.619 | 4.89E-02 | hypothetical protein |
|  | SCO5949 | 0.619 | 1.78E-02 | hypothetical protein |
|  | SCO3110 | 0.618 | 3.99E-02 | ABC transporter |
|  | SCO2236 | 0.618 | 3.70E-02 | hypothetical protein |
|  | SCO7824 | 0.618 | 9.17E-03 | TetR family transcriptional regulator |
|  | SCO4609 | 0.615 | 3.61E-02 | heat shock protein HtpX |
|  | SCO4566 | 0.608 | 1.61E-02 | NADH dehydrogenase subunit E |
|  | SCO5009 | 0.606 | 3.45E-03 | secretory protein |
|  | SCO5851 | 0.604 | 1.22E-02 | hypothetical protein |
|  | SCO0180 | 0.604 | 2.74E-02 | hypothetical protein |
|  | SCO1144 | 0.601 | 1.69E-03 | ABC transporter ATP-binding protein |
|  | SCO5817 | 0.600 | 2.43E-02 | DNA hydrolase |
|  | SCO3563 | 0.597 | 3.44E-02 | acetyl-CoA synthetase |
|  | SCO6005 | 0.595 | 7.06E-03 | lipoprotein |
|  | SCO0170 | 0.595 | 3.47E-02 | hypothetical protein |
|  | SCO5811 | 0.593 | 4.22E-03 | transcriptional regulator |
|  | SCO6513 | 0.591 | 1.65E-02 | hypothetical protein |
|  | SCO2465 | 0.589 | 3.62E-02 | RNA polymerase principal sigma factor |
|  | SCO5333 | 0.586 | 3.58E-02 | hypothetical protein |
|  | SCO1817 | 0.584 | 1.04E-02 | hypothetical protein |
|  | SCO4309 | 0.584 | 4.09E-02 | hypothetical protein |
|  | SCO4279 | 0.583 | 4.00E-02 | acetyltransferase |
|  | SCO3323 | 0.579 | 2.35E-02 | RNA polymerase sigma factor |
|  | SCO2311 | 0.577 | 4.01E-03 | hypothetical protein |
|  | SCO6045 | 0.575 | 9.54E-03 | hypothetical protein |
|  | SCO4827 | 0.574 | 4.80E-02 | malate dehydrogenase |
|  | SCO4020 | 0.571 | 7.51E-03 | two component system response regulator |
|  | SCO3289 | 0.564 | 1.12E-02 | large membrane protein |
|  | SCO4075 | 0.563 | 8.65E-03 | ABC transporter ATP-binding protein |
|  | SCO7005 | 0.562 | 6.28E-03 | oxidoreductase |
|  | SCO7367 | 0.559 | 4.22E-03 | membrane efflux protein |
|  | SCO6149 | 0.551 | 2.00E-02 | ribosome-associated GTPase |
|  | SCO5629 | 0.538 | 6.65E-04 | ATP /GTP-binding protein |
|  | SCO4630 | 0.534 | 3.47E-02 | hypothetical protein |
|  | SCO4188 | 0.530 | 6.07E-03 | GntR family transcriptional regulator |
|  | SCO2861 | 0.527 | 2.79E-02 | hypothetical protein |
|  | SCO3288 | 0.527 | 4.46E-02 | hypothetical protein |
|  | SCO3826 | 0.518 | 8.81E-03 | ion channel membrane protein |
|  | SCO2018 | 0.514 | 5.02E-02 | aminopeptidase |
|  | SCP2.02 | 0.508 | 1.36E-02 | putative is1648 transposase |
|  | SCO5810 | 0.504 | 2.30E-02 | transmembrane efflux protein |
|  | SCO4157 | 0.501 | 5.50E-03 | protease |
|  | SCO4290 | 0.501 | 1.15E-02 | trehalose-phosphate synthase |
|  | SCO1727 | 0.500 | 2.80E-02 | hypothetical protein |
| Downregulated | SCO6564 | -0.509 | 3.30E-02 | 3-oxoacyl-ACP synthase III |
|  | SCO4740 | -0.510 | 2.42E-02 | glucosamine--fructose-6-phosphate aminotransferase |
|  | SCO5516 | -0.511 | 1.24E-02 | integral membrane efflux protein |
|  | SCO1620 | -0.516 | 1.29E-02 | glycine/betaine ABC transporter permease |
|  | SCO3311 | -0.536 | 2.86E-02 | delta-aminolevulinic acid dehydratase |
|  | SCO1683 | -0.538 | 1.07E-03 | amino acid permease |
|  | SCO4347 | -0.567 | 7.16E-04 | hypothetical protein |
|  | SCO0104 | -0.582 | 3.87E-02 | hydrolase |
|  | SCO1105 | -0.594 | 5.64E-04 | hypothetical protein |
|  | SCO1374 | -0.608 | 6.03E-04 | hypothetical protein |
|  | SCO2256 | -0.616 | 4.06E-04 | 3-methyl-2-oxobutanoate hydroxymethyltransferase |
|  | SCO0496 | -0.624 | 1.61E-02 | iron-siderophore permease transmembrane protein |
|  | SCO1487 | -0.628 | 6.16E-03 | aspartate carbamoyltransferase catalytic subunit |
|  | SCO5281 | -0.635 | 4.47E-02 | alpha-ketoglutarate decarboxylase |
|  | SCO2995 | -0.641 | 1.12E-03 | ABC transporter |
|  | SCO6453 | -0.651 | 3.72E-02 | transport permease |
|  | SCO1946 | -0.651 | 3.61E-02 | phosphoglycerate kinase |
|  | SCO1621 | -0.662 | 1.10E-02 | glycine/betaine transport ATP-binding protein |
|  | SCO4739 | -0.672 | 2.00E-02 | lipoprotein |
|  | SCO4950 | -0.677 | 3.29E-02 | nitrate reductase subunit gamma NarI3 |
|  | SCO1441 | -0.679 | 1.76E-02 | bifunctional 3%2C4-dihydroxy-2-butanone 4-phosphate synthase/GTP cyclohydrolase II protein |
|  | SCO2267 | -0.691 | 2.27E-03 | heme oxygenase |
|  | SCO1776 | -0.707 | 1.44E-02 | CTP synthetase |
|  | SCO2389 | -0.710 | 3.79E-02 | acyl carrier protein |
|  | SCO0958 | -0.710 | 3.62E-04 | hypothetical protein |
|  | SCO1246 | -0.718 | 2.51E-03 | dithiobiotin synthetase |
|  | SCO2241 | -0.722 | 6.03E-03 | glutamine synthetase |
|  | SCO1481 | -0.729 | 1.45E-02 | orotidine 5'-phosphate decarboxylase |
|  | SCO5515 | -0.749 | 2.50E-02 | D-3-phosphoglycerate dehydrogenase |
|  | SCO4948 | -0.771 | 5.89E-03 | nitrate reductase subunit beta NarH3 |
|  | SCO4949 | -0.775 | 3.70E-02 | nitrate reductase subunit delta NarJ3 |
|  | SCO5650 | -0.783 | 2.68E-02 | hypothetical protein |
|  | SCO6451 | -0.783 | 2.33E-03 | substrate binding protein |
|  | SCO1916 | -0.784 | 1.17E-02 | 2%2C3%2C4%2C5-tetrahydropyridine-2%2C6-dicarboxylate N-succinyltransferase |
|  | SCO6770 | -0.792 | 1.41E-02 | DNA-binding protein |
|  | SCO2388 | -0.797 | 3.93E-02 | 3-oxoacyl-ACP synthase III |
|  | SCO3127 | -0.812 | 1.60E-04 | phosphoenolpyruvate carboxylase |
|  | SCO2276 | -0.812 | 1.53E-04 | hypothetical protein |
|  | SCO2195 | -0.824 | 7.15E-03 | hypothetical protein |
|  | SCO2390 | -0.836 | 9.11E-03 | 3-oxoacyl-ACP synthase |
|  | SCO2528 | -0.850 | 2.66E-03 | 2-isopropylmalate synthase |
|  | SCO1482 | -0.873 | 6.55E-05 | dihydroorotate dehydrogenase 2 |
|  | SCO2630 | -0.881 | 7.64E-04 | biotin synthase |
|  | SCO0488 | -0.892 | 9.00E-05 | hydrolase |
|  | SCO4048 | -0.905 | 3.02E-03 | hypothetical protein |
|  | SCO5536 | -0.929 | 6.83E-03 | hypothetical protein |
|  | SCO2025 | -0.934 | 7.51E-04 | glutamate synthase |
|  | SCO0073 | -0.940 | 1.22E-05 | hypothetical protein |
|  | SCO4501 | -0.956 | 3.13E-04 | 3-ketoacyl-ACP reductase |
|  | SCO5535 | -0.959 | 1.48E-03 | carboxyl transferase |
|  | SCO5470 | -0.974 | 9.56E-05 | serine hydroxymethyltransferase |
|  | SCO4047 | -0.976 | 6.59E-05 | hypothetical protein |
|  | SCO4947 | -0.979 | 2.69E-11 | nitrate reductase subunit alpha NarG3 |
|  | SCO1245 | -1.011 | 1.56E-05 | adenosylmethionine--8-amino-7-oxononanoate aminotransferase BioA |
|  | SCO2383 | -1.012 | 7.17E-06 | hypothetical protein |
|  | SCO1814 | -1.023 | 2.71E-03 | enoyl-ACP reductase |
|  | SCO4838 | -1.044 | 1.14E-06 | hypothetical protein |
|  | SCO2026 | -1.059 | 2.25E-05 | glutamate synthase |
|  | SCO6426 | -1.069 | 9.42E-03 | hypothetical protein |
|  | SCO1235 | -1.108 | 1.69E-03 | urease subunit beta |
|  | SCO1244 | -1.121 | 5.50E-06 | biotin synthase |
|  | SCO0888 | -1.157 | 2.57E-06 | hypothetical protein |
|  | SCO0631 | -1.160 | 1.46E-04 | hypothetical protein |
|  | SCO2633 | -1.166 | 3.68E-05 | superoxide dismutase |
|  | SCO0497 | -1.191 | 1.71E-04 | iron-siderophore permease transmembrane protein |
|  | SCO2268 | -1.247 | 3.56E-08 | hypothetical protein |
|  | SCO2789 | -1.247 | 1.02E-09 | glucosamine-fructose-6-phosphate aminotransferase |
|  | SCO2272 | -1.250 | 1.71E-05 | binding-protein-dependent transport lipoprotein |
|  | SCO4159 | -1.265 | 1.94E-06 | transcriptional regulator |
|  | SCO1294 | -1.294 | 6.43E-04 | cystathionine gamma-synthase |
|  | SCO6158 | -1.316 | 2.33E-14 | hypothetical protein |
|  | SCO5830 | -1.378 | 8.42E-10 | hypothetical protein |
|  | SCO1947 | -1.410 | 1.05E-08 | glyceraldehyde-3-phosphate dehydrogenase |
|  | SCO2275 | -1.411 | 8.86E-16 | lipoprotein |
|  | SCO0491 | -1.524 | 8.54E-14 | ABC transporter |
|  | SCO0495 | -1.569 | 6.97E-08 | iron-siderophore ABC-transporter ATP-binding protein |
|  | SCO2781 | -1.584 | 4.86E-14 | hypothetical protein |
|  | SCO2958 | -1.587 | 6.16E-10 | bifunctional uroporphyrinogen-III synthetase/response regulator domain-containing protein |
|  | SCO0490 | -1.615 | 9.30E-18 | esterase |
|  | SCO2471 | -1.638 | 1.70E-06 | hypothetical protein |
|  | SCO1293 | -1.725 | 1.16E-15 | hypothetical protein |
|  | SCO1787 | -1.768 | 5.93E-14 | iron-siderophore uptake system transmembrane protein |
|  | SCO1623 | -1.770 | 1.00E-10 | hypothetical protein |
|  | SCO2785 | -1.964 | 2.43E-14 | hypothetical protein |
|  | SCO0492 | -1.971 | 1.09E-42 | peptide synthetase |
|  | SCO2784 | -1.988 | 5.66E-13 | aceytltranferase |
|  | SCO0799 | -1.994 | 1.05E-13 | hypothetical protein |
|  | SCO7398 | -2.013 | 1.58E-40 | iron-hydroxamate transporter permease subunit |
|  | SCO2782 | -2.098 | 2.39E-13 | pyridoxal-dependent decarboxylase |
|  | SCO0681 | -2.385 | 3.42E-22 | ferredoxin/ferredoxin-NADP reductase |
|  | SCO2783 | -2.400 | 1.71E-29 | monooxygenase |
|  | SCO1786 | -2.507 | 5.41E-30 | iron-siderophore uptake system transmembrane protein |
|  | SCO0493 | -2.512 | 1.85E-23 | ABC-transporter transmembrane protein |
|  | SCO0489 | -2.533 | 1.33E-14 | hypothetical protein |
|  | SCO2271 | -2.632 | 2.13E-28 | hypothetical protein |
|  | SCO2198 | -2.701 | 7.94E-30 | glutamine synthetase |
|  | SCO1785 | -2.748 | 8.66E-37 | iron-siderophore uptake system ATP-binding protein |
|  | SCO2270 | -2.754 | 2.92E-37 | hypothetical protein |
|  | SCO2211 | -3.363 | 2.56E-68 | hypothetical protein |
|  | SCO0494 | -3.376 | 4.33E-39 | iron-siderophore binding lipoprotein |
|  | SCO0499 | -3.493 | 1.50E-39 | formyltransferase |
|  | SCO0459 | -3.495 | 1.37E-50 | hypothetical protein |
|  | SCO2780 | -3.574 | 1.45E-64 | hypothetical protein |
|  | SCO5584 | -3.713 | 5.70E-51 | nitrogen regulatory protein P-II |
|  | SCO7400 | -3.798 | 1.86E-57 | ABC transporter ATP-binding protein |
|  | SCO5585 | -3.803 | 3.70E-51 | PII uridylyl-transferase |
|  | SCO0498 | -3.884 | 5.48E-98 | peptide monooxygenase |
|  | SCO7399 | -4.136 | 6.64E-71 | binding-protein-dependent transport lipoprotein |
|  | SCO5583 | -4.931 | 1.17E-18 | ammonium transporter |
|  | SCO2210 | -6.000 | 2.99E-03 | glutamine synthetase |

1h-2h

|  | Locus_tag | log2  Fold-change | FDR | Product |
| --- | --- | --- | --- | --- |
| upregulated | SCO4425 | 2.733 | 2.14E-18 | sigma-like protein |
|  | SCO0494 | 2.675 | 2.43E-24 | iron-siderophore binding lipoprotein |
|  | SCO2780 | 2.529 | 1.06E-31 | hypothetical protein |
|  | SCO4225 | 2.447 | 3.79E-15 | hypothetical protein |
|  | SCO7460 | 2.362 | 2.97E-14 | lipoprotein |
|  | SCO7399 | 2.238 | 4.38E-20 | binding-protein-dependent transport lipoprotein |
|  | SCO3230 | 2.207 | 3.97E-02 | CDA peptide synthetase I |
|  | SCO1773 | 2.148 | 1.09E-17 | L-alanine dehydrogenase |
|  | SCO7613 | 2.100 | 8.03E-14 | hypothetical protein |
|  | SCO0493 | 2.089 | 4.92E-16 | ABC-transporter transmembrane protein |
|  | SCO7400 | 2.070 | 2.10E-16 | ABC transporter ATP-binding protein |
|  | SCO0459 | 2.049 | 7.53E-17 | hypothetical protein |
|  | SCO0499 | 2.027 | 4.76E-13 | formyltransferase |
|  | SCO7461 | 1.987 | 5.09E-11 | hydrolase |
|  | SCO1786 | 1.892 | 1.01E-16 | iron-siderophore uptake system transmembrane protein |
|  | SCO3900 | 1.873 | 9.72E-09 | hypothetical protein |
|  | SCO0495 | 1.797 | 2.63E-10 | iron-siderophore ABC-transporter ATP-binding protein |
|  | SCO1785 | 1.787 | 3.79E-15 | iron-siderophore uptake system ATP-binding protein |
|  | SCO4224 | 1.784 | 1.42E-11 | hypothetical protein |
|  | SCO6682 | 1.779 | 1.42E-08 | hypothetical protein |
|  | SCO1440 | 1.745 | 1.98E-07 | 6%2C7-dimethyl-8-ribityllumazine synthase |
|  | SCO0607 | 1.711 | 9.83E-07 | lipoprotein |
|  | SCO0498 | 1.702 | 4.54E-18 | peptide monooxygenase |
|  | SCO1787 | 1.675 | 1.65E-12 | iron-siderophore uptake system transmembrane protein |
|  | SCO0736 | 1.667 | 7.53E-06 | hypothetical protein |
|  | SCO1550 | 1.666 | 1.42E-04 | small membrane protein |
|  | SCO1623 | 1.582 | 1.43E-08 | hypothetical protein |
|  | SCO7612 | 1.544 | 4.65E-10 | hypothetical protein |
|  | SCOt40 | 1.473 | 2.33E-03 | tRNA-Gly |
|  | SCO4011 | 1.470 | 1.37E-07 | hypothetical protein |
|  | SCO4635 | 1.454 | 5.56E-04 | 50S ribosomal protein L33 |
|  | SCO4924 | 1.397 | 7.29E-05 | hypothetical protein |
|  | SCO4710 | 1.386 | 5.53E-04 | 50S ribosomal protein L29 |
|  | SCO3356 | 1.358 | 4.23E-05 | ECF sigma factor |
|  | SCO6729 | 1.354 | 1.50E-06 | hypothetical protein |
|  | SCO5830 | 1.351 | 2.49E-09 | hypothetical protein |
|  | SCO3899 | 1.324 | 2.91E-07 | hypothetical protein |
|  | SCO2275 | 1.319 | 1.61E-13 | lipoprotein |
|  | SCO5841 | 1.289 | 4.28E-05 | phosphocarrier protein HPr |
|  | SCO3862 | 1.278 | 2.20E-03 | hypothetical protein |
|  | SCO3924 | 1.275 | 1.59E-04 | hypothetical protein |
|  | SCO1638 | 1.256 | 1.63E-03 | peptidyl-prolyl cis-trans isomerase |
|  | SCO2648 | 1.245 | 2.93E-04 | hypothetical protein |
|  | SCO2947 | 1.244 | 2.67E-03 | hypothetical protein |
|  | SCO5436 | 1.228 | 6.16E-09 | sodium:dicarboxylate symporter |
|  | SCO0497 | 1.212 | 1.14E-04 | iron-siderophore permease transmembrane protein |
|  | SCOr09 | 1.209 | 2.66E-03 | 16S ribosomal RNA |
|  | SCO2066 | 1.189 | 3.43E-04 | hypothetical protein |
|  | SCO3870 | 1.181 | 1.95E-03 | hypothetical protein |
|  | SCO4713 | 1.178 | 3.04E-03 | 50S ribosomal protein L24 |
|  | SCO4847 | 1.153 | 5.63E-05 | D-alanyl-D-alanine carboxypeptidase |
|  | SCO0492 | 1.152 | 2.16E-14 | peptide synthetase |
|  | SCO4720 | 1.146 | 2.95E-03 | 50S ribosomal protein L30 |
|  | SCO6509 | 1.140 | 2.67E-03 | hydrophobic protein |
|  | SCO0681 | 1.118 | 5.57E-05 | ferredoxin/ferredoxin-NADP reductase |
|  | SCO4725 | 1.117 | 2.36E-03 | translation initiation factor IF-1 |
|  | SCO2633 | 1.111 | 9.58E-05 | superoxide dismutase |
|  | SCO1916 | 1.106 | 4.32E-05 | 2%2C3%2C4%2C5-tetrahydropyridine-2%2C6-dicarboxylate N-succinyltransferase |
|  | SCO4652 | 1.102 | 4.61E-03 | 50S ribosomal protein L10 |
|  | SCO1319 | 1.096 | 1.26E-03 | hypothetical protein |
|  | SCO0609 | 1.093 | 7.25E-04 | hypothetical protein |
|  | SCOt36 | 1.091 | 1.96E-02 | tRNA-Arg |
|  | SCO4716 | 1.091 | 2.67E-03 | 30S ribosomal protein S8 |
|  | SCO5875 | 1.084 | 2.01E-04 | potassium uptake protein |
|  | SCO4137 | 1.081 | 2.03E-03 | hypothetical protein |
|  | SCO1490 | 1.080 | 6.58E-03 | transcription antitermination protein NusB |
|  | SCO2627 | 1.077 | 2.67E-03 | ribose-5-phosphate isomerase B |
|  | SCO6722 | 1.067 | 5.89E-04 | regulator |
|  | SCO4091 | 1.067 | 3.29E-03 | DNA-binding protein |
|  | SCO5145 | 1.064 | 1.79E-02 | hypothetical protein |
|  | SCO1254 | 1.061 | 4.66E-03 | adenylosuccinate lyase |
|  | SCO6452 | 1.055 | 6.87E-04 | transport permease |
|  | SCO1944 | 1.054 | 7.28E-03 | preprotein translocase subunit SecG |
|  | SCO2105 | 1.046 | 1.39E-02 | transcriptional regulator |
|  | SCO3194 | 1.044 | 1.77E-04 | lipoprotein |
|  | SCO3909 | 1.044 | 1.03E-02 | 50S ribosomal protein L9 |
|  | SCO2789 | 1.040 | 8.34E-07 | glucosamine-fructose-6-phosphate aminotransferase |
|  | SCO4506 | 1.039 | 1.87E-04 | hypothetical protein |
|  | SCO6158 | 1.038 | 1.26E-08 | hypothetical protein |
|  | SCO0608 | 1.036 | 6.53E-04 | regulatory protein |
|  | SCO3546 | 1.029 | 1.84E-03 | hypothetical protein |
|  | SCO4077 | 1.026 | 2.40E-02 | phosphoribosylformylglycinamidine synthase subunit PurS |
|  | SCO1700 | 1.023 | 1.77E-05 | hypothetical protein |
|  | SCO0703 | 1.009 | 4.61E-03 | regulator |
|  | SCO5464 | 1.005 | 2.80E-03 | hypothetical protein |
|  | SCO5797 | 1.004 | 1.05E-03 | hypothetical protein |
|  | SCO2303 | 1.001 | 5.97E-03 | hypothetical protein |
|  | SCO3575 | 0.997 | 2.67E-03 | hypothetical protein |
|  | SCO6225 | 0.995 | 1.47E-04 | hypothetical protein |
|  | SCO1514 | 0.984 | 5.60E-03 | adenine phosphoribosyltransferase |
|  | SCO3822 | 0.980 | 9.99E-03 | hypothetical protein |
|  | SCOs01 | 0.978 | 2.48E-02 | ribonuclease P RNA |
|  | SCO0491 | 0.974 | 1.33E-05 | ABC transporter |
|  | SCO4813 | 0.973 | 4.64E-03 | phosphoribosylglycinamide formyltransferase |
|  | SCO4492 | 0.971 | 1.19E-03 | octaprenyl carboxylase |
|  | SCO3327 | 0.971 | 1.74E-02 | hypothetical protein |
|  | SCOr13, SCOr14, SCOr15 | 0.971 | 5.03E-03 | 16S ribosomal RNA, 23S ribosomal RNA, 5S ribosomal RNA |
|  | SCO2888 | 0.966 | 1.35E-02 | hypothetical protein |
|  | SCO4992 | 0.963 | 1.63E-02 | hypothetical protein |
|  | SCO4561 | 0.962 | 7.20E-03 | hypothetical protein |
|  | SCO1699 | 0.962 | 2.88E-05 | transcriptional regulator |
|  | SCO1391 | 0.954 | 3.18E-03 | phosphoenolpyruvate-protein phosphotransferase |
|  | SCO4037 | 0.952 | 1.18E-02 | small membrane protein |
|  | SCO4721 | 0.949 | 6.58E-03 | 50S ribosomal protein L15 |
|  | SCOr10, SCOr11, SCOr12 | 0.948 | 9.10E-03 | 5S ribosomal RNA, 23S ribosomal RNA, 16S ribosomal RNA |
|  | SCO5742 | 0.944 | 1.01E-02 | hypothetical protein |
|  | SCO6224 | 0.944 | 1.22E-03 | hypothetical protein |
|  | SCO1602 | 0.941 | 2.25E-03 | hypothetical protein |
|  | SCO1790 | 0.940 | 7.18E-03 | hypothetical protein |
|  | SCOr4, SCOr5, SCOr6 | 0.935 | 7.96E-03 | 5S ribosomal RNA, 23S ribosomal RNA, 16S ribosomal RNA |
|  | SCO3906 | 0.934 | 1.51E-02 | 30S ribosomal protein S6 |
|  | SCO2019 | 0.933 | 4.12E-03 | chorismate mutase |
|  | SCO2899 | 0.929 | 1.70E-02 | GroES family molecular chaperone |
|  | SCO3396 | 0.928 | 2.58E-05 | hypothetical protein |
|  | SCO4885 | 0.927 | 5.54E-04 | lipoprotein |
|  | SCO3881 | 0.924 | 1.55E-02 | ribonuclease P component |
|  | SCO1548 | 0.917 | 9.99E-03 | hypothetical protein |
|  | SCO7398 | 0.917 | 7.88E-08 | iron-hydroxamate transporter permease subunit |
|  | SCO3397 | 0.915 | 1.34E-04 | integral membrane lysyl-tRNA synthetase |
|  | SCOr16, SCOr17, SCOr18 | 0.915 | 7.51E-03 | 16S ribosomal RNA, 23S ribosomal RNA, 5S ribosomal RNA |
|  | SCOt31 | 0.912 | 2.55E-02 | tRNA-Leu |
|  | SCO2041 | 0.911 | 1.03E-02 | hypothetical protein |
|  | SCO1375 | 0.910 | 1.21E-02 | hypothetical protein |
|  | SCO7657 | 0.909 | 4.66E-03 | hypothetical protein |
|  | SCO4719 | 0.906 | 1.57E-02 | 30S ribosomal protein S5 |
|  | SCO5151 | 0.904 | 6.08E-03 | hypothetical protein |
|  | SCO2370 | 0.904 | 3.12E-02 | hypothetical protein |
|  | SCO3877 | 0.902 | 1.57E-02 | 6-phosphogluconate dehydrogenase |
|  | SCO1600 | 0.902 | 2.96E-02 | translation initiation factor IF-3 |
|  | SCO3092 | 0.900 | 7.21E-03 | oxidoreductase |
|  | SCOr1, SCOr2, SCOr3 | 0.898 | 7.55E-03 | 5S ribosomal RNA, 23S ribosomal RNA, 16S ribosomal RNA |
|  | SCO1495 | 0.897 | 4.52E-02 | shikimate kinase |
|  | SCO3897 | 0.896 | 9.79E-03 | hypothetical protein |
|  | SCO3290 | 0.894 | 9.49E-06 | hypothetical protein |
|  | SCO2781 | 0.894 | 1.77E-04 | hypothetical protein |
|  | SCOr08 | 0.894 | 1.18E-02 | 23S ribosomal RNA |
|  | SCO2788 | 0.890 | 2.84E-03 | hypothetical protein |
|  | SCO4715 | 0.890 | 1.23E-02 | 30S ribosomal protein S14 |
|  | SCO5230 | 0.888 | 3.97E-02 | hypothetical protein |
|  | SCO4686 | 0.887 | 1.91E-02 | hypothetical protein |
|  | SCO0695 | 0.886 | 4.45E-03 | hypothetical protein |
|  | SCO7322 | 0.885 | 3.09E-02 | anti-sigma factor |
|  | SCO1775 | 0.883 | 1.35E-02 | hypothetical protein |
|  | SCO1442 | 0.883 | 2.86E-03 | hypothetical protein |
|  | SCO2198 | 0.883 | 2.12E-03 | glutamine synthetase |
|  | SCO6997 | 0.881 | 3.12E-02 | hypothetical protein |
|  | SCO1601 | 0.881 | 1.83E-02 | hypothetical protein |
|  | SCO5720 | 0.881 | 6.50E-03 | hypothetical protein |
|  | SCO0999 | 0.880 | 1.18E-02 | superoxide dismutase |
|  | SCO6770 | 0.877 | 3.29E-03 | DNA-binding protein |
|  | SCO5492 | 0.875 | 7.74E-03 | short chain dehydrogenase |
|  | SCO3880 | 0.875 | 1.59E-02 | 50S ribosomal protein L34 |
|  | SCO2034 | 0.870 | 2.17E-02 | prolipoprotein diacylglyceryl transferase |
|  | SCO2089 | 0.869 | 3.04E-03 | UDP-N-acetylmuramoylalanyl-D-glutamate--2%2C6-diaminopimelate ligase |
|  | SCO6990 | 0.864 | 5.07E-04 | membrane transport protein |
|  | SCO0490 | 0.863 | 5.62E-05 | esterase |
|  | SCO4033 | 0.862 | 2.91E-02 | hypothetical protein |
|  | SCO1114 | 0.862 | 1.13E-02 | uracil-DNA glycosylase |
|  | SCO6989 | 0.861 | 7.62E-04 | hypothetical protein |
|  | SCO2546 | 0.860 | 4.77E-04 | adenosine deaminase |
|  | SCO1492 | 0.859 | 1.58E-02 | peptidase |
|  | SCO3289 | 0.858 | 4.97E-06 | large membrane protein |
|  | SCO1793 | 0.857 | 7.09E-03 | hypothetical protein |
|  | SCO2457 | 0.851 | 2.83E-03 | lipoprotein |
|  | SCO4653 | 0.851 | 2.18E-02 | 50S ribosomal protein L7/L12 |
|  | SCO3974 | 0.850 | 3.30E-03 | hypothetical protein |
|  | SCO1400 | 0.848 | 4.39E-02 | hypothetical protein |
|  | SCO3887 | 0.847 | 1.89E-02 | partitioning or sporulation protein |
|  | SCO5568 | 0.845 | 8.52E-03 | phosphopantetheine adenylyltransferase |
|  | SCO5481 | 0.844 | 3.65E-04 | hypothetical protein |
|  | SCO2593 | 0.844 | 1.54E-02 | hypothetical protein |
|  | SCO1395 | 0.841 | 1.08E-02 | mutT-like protein |
|  | SCO3898 | 0.840 | 3.74E-05 | hypothetical protein |
|  | SCO0799 | 0.838 | 1.32E-02 | hypothetical protein |
|  | SCO3124 | 0.835 | 1.74E-02 | 50S ribosomal protein L25/general stress protein Ctc |
|  | SCO5724 | 0.833 | 3.41E-02 | hypothetical protein |
|  | SCO5798 | 0.830 | 7.51E-03 | hypothetical protein |
|  | SCO2129 | 0.829 | 1.83E-02 | hypothetical protein |
|  | SCO6482 | 0.829 | 2.92E-02 | hypothetical protein |
|  | SCO3579 | 0.824 | 4.84E-02 | regulatory protein |
|  | SCO2025 | 0.824 | 3.52E-03 | glutamate synthase |
|  | SCO5504 | 0.823 | 6.50E-03 | hypothetical protein |
|  | SCO2949 | 0.823 | 4.18E-03 | UDP-N-acetylglucosamine 1-carboxyvinyltransferase |
|  | SCO4923 | 0.820 | 1.83E-02 | hypothetical protein |
|  | SCO6139 | 0.819 | 6.77E-03 | sensor kinase |
|  | SCO3357 | 0.815 | 3.81E-03 | hypothetical protein |
|  | SCO3097 | 0.815 | 1.24E-03 | hypothetical protein |
|  | SCO0536 | 0.814 | 1.57E-02 | hypothetical protein |
|  | SCO5588 | 0.812 | 6.02E-03 | lipoprotein |
|  | SCO2950 | 0.810 | 2.13E-02 | DNA-binding protein HU (hs1) |
|  | SCO1470 | 0.809 | 2.17E-02 | hypothetical protein |
|  | SCO1791 | 0.809 | 9.34E-03 | hypothetical protein |
|  | SCO5833 | 0.808 | 1.09E-02 | hypothetical protein |
|  | SCO5570 | 0.808 | 4.32E-02 | hypothetical protein |
|  | SCO4371 | 0.806 | 4.16E-03 | hypothetical protein |
|  | SCO0462 | 0.802 | 1.18E-02 | oxidoreductase |
|  | SCO4726 | 0.801 | 3.12E-02 | 50S ribosomal protein L36 |
|  | SCO4848 | 0.800 | 5.69E-03 | hypothetical protein |
|  | SCO1691 | 0.799 | 1.19E-02 | TetR family transcriptional regulator |
|  | SCO3629 | 0.797 | 3.63E-03 | adenylosuccinate synthetase |
|  | SCO7233 | 0.797 | 3.34E-03 | hypothetical protein |
|  | SCO5413 | 0.797 | 2.17E-02 | MarR-transcriptional regulator |
|  | SCO3677 | 0.796 | 2.41E-02 | purine phosphoribosyltransferase |
|  | SCO2154 | 0.796 | 3.10E-02 | hypothetical protein |
|  | SCO2456 | 0.795 | 3.88E-03 | hypothetical protein |
|  | SCO6589 | 0.792 | 3.27E-02 | elongation factor G |
|  | SCO5115 | 0.792 | 1.19E-02 | ABC transporter intracellular ATPase subunit BldKD |
|  | SCO1401 | 0.791 | 2.94E-02 | hypothetical protein |
|  | SCO3811 | 0.789 | 1.34E-04 | D-alanyl-D-alanine carboxypeptidase |
|  | SCO1755 | 0.787 | 6.44E-03 | hypothetical protein |
|  | SCO4509 | 0.786 | 2.34E-02 | hypothetical protein |
|  | SCO4636 | 0.786 | 3.10E-02 | hypothetical protein |
|  | SCO2268 | 0.783 | 2.32E-03 | hypothetical protein |
|  | SCO6757 | 0.783 | 3.61E-02 | ABC transporter membrane protein |
|  | SCO5541 | 0.783 | 4.50E-02 | ATP-GTP binding protein |
|  | SCO4637 | 0.780 | 1.06E-02 | hypothetical protein |
|  | SCO3613 | 0.779 | 2.84E-02 | RNA polymerase sigma factor |
|  | SCO2458 | 0.778 | 1.48E-02 | hypothetical protein |
|  | SCO1235 | 0.778 | 4.65E-02 | urease subunit beta |
|  | SCO1441 | 0.775 | 2.89E-03 | bifunctional 3%2C4-dihydroxy-2-butanone 4-phosphate synthase/GTP cyclohydrolase II protein |
|  | SCO4161 | 0.775 | 2.17E-02 | molybdopterin converting factor |
|  | SCO1464 | 0.775 | 2.53E-02 | ribulose-phosphate 3-epimerase |
|  | SCO3676 | 0.775 | 1.77E-02 | hypothetical protein |
|  | SCO1945 | 0.775 | 3.51E-02 | triosephosphate isomerase |
|  | SCO3619 | 0.774 | 4.45E-02 | hypothetical protein |
|  | SCO6031 | 0.773 | 3.43E-02 | uroporphyrinogen decarboxylase |
|  | SCO2088 | 0.772 | 1.03E-02 | UDP-N-acetylmuramoylalanyl-D-glutamyl-2%2C6-diaminopimelate- D-alanyl-alanyl ligase |
|  | SCO1320 | 0.768 | 3.86E-03 | hypothetical protein |
|  | SCO4718 | 0.767 | 3.13E-02 | 50S ribosomal protein L18 |
|  | SCO1390 | 0.763 | 2.12E-02 | PTS system sugar phosphotransferase component IIA |
|  | SCO3288 | 0.762 | 2.77E-04 | hypothetical protein |
|  | SCO3180 | 0.762 | 4.94E-02 | molybdenum cofactor biosynthesis protein MoaC |
|  | SCO5459 | 0.760 | 5.03E-03 | enoyl-CoA hydratase |
|  | SCO1806 | 0.759 | 1.42E-02 | ABC transporter ATP-binding protein |
|  | SCO5112 | 0.757 | 1.03E-02 | ABC transporter integral membrane protein BldKA |
|  | SCO5491 | 0.751 | 3.13E-02 | hypothetical protein |
|  | SCO0627 | 0.751 | 4.49E-02 | ATP-utilizing protein |
|  | SCO5856 | 0.750 | 7.20E-03 | hypothetical protein |
|  | SCO2067 | 0.750 | 5.55E-03 | hypothetical protein |
|  | SCO5470 | 0.749 | 4.75E-03 | serine hydroxymethyltransferase |
|  | SCO5515 | 0.748 | 1.60E-02 | D-3-phosphoglycerate dehydrogenase |
|  | SCO4424 | 0.746 | 1.90E-03 | hypothetical protein |
|  | SCO5537 | 0.745 | 3.49E-02 | ATP/GTP binding protein |
|  | SCO7284 | 0.743 | 4.01E-02 | ribonuclease H |
|  | SCO6774 | 0.743 | 1.04E-02 | hypothetical protein |
|  | SCO5313 | 0.743 | 7.32E-03 | hypothetical protein |
|  | SCO1566 | 0.740 | 3.00E-02 | acyltransferase |
|  | SCO1710 | 0.738 | 9.27E-04 | integral membrane transport protein |
|  | SCO3678 | 0.737 | 2.13E-02 | deoxycytidine triphosphate deaminase |
|  | SCO4087 | 0.736 | 3.33E-02 | phosphoribosylaminoimidazole synthetase |
|  | SCO2243 | 0.733 | 4.05E-02 | TetR family transcriptional regulator |
|  | SCO2628 | 0.731 | 2.36E-02 | amino acid permease |
|  | SCO3761 | 0.727 | 3.27E-02 | hypothetical protein |
|  | SCO5664 | 0.721 | 2.81E-02 | hypothetical protein |
|  | SCO2069 | 0.720 | 1.83E-02 | hypothetical protein |
|  | SCO3342 | 0.718 | 6.60E-03 | glycine-rich hypothetical protein |
|  | SCO5876 | 0.717 | 1.79E-02 | potassium uptake protein |
|  | SCO3572 | 0.716 | 3.18E-02 | hydrolase |
|  | SCO1875 | 0.712 | 5.61E-03 | penicillin binding protein |
|  | SCO3911 | 0.711 | 3.06E-03 | replicative DNA helicase |
|  | SCO6431 | 0.710 | 2.59E-03 | peptide synthase |
|  | SCO6763 | 0.709 | 4.20E-02 | polyprenyl synthatase |
|  | SCO5393 | 0.709 | 3.74E-02 | ABC transporter ATP-binding protein |
|  | SCO0735 | 0.709 | 8.07E-03 | oxidoreductase |
|  | SCO0260 | 0.707 | 4.12E-02 | hypothetical protein |
|  | SCO4172 | 0.705 | 2.40E-03 | hypothetical protein |
|  | SCO3127 | 0.704 | 1.50E-03 | phosphoenolpyruvate carboxylase |
|  | SCO5679 | 0.703 | 1.12E-02 | aldehyde dehydrogenase |
|  | SCO4439 | 0.702 | 2.53E-02 | D-alanyl-D-alanine carboxypeptidase |
|  | SCO4002 | 0.702 | 3.72E-03 | hypothetical protein |
|  | SCO5114 | 0.701 | 1.18E-02 | ABC transporter integral membrane protein BldKC |
|  | SCO6715 | 0.698 | 3.97E-02 | transcriptional regulator |
|  | SCO6430 | 0.697 | 8.98E-03 | hypothetical protein |
|  | SCO1527 | 0.697 | 4.64E-02 | membrane transferase |
|  | SCO4648 | 0.696 | 2.36E-02 | 50S ribosomal protein L11 |
|  | SCO5425 | 0.695 | 7.20E-03 | phosphate acetyltransferase |
|  | SCO3807 | 0.692 | 8.25E-03 | hydrolase |
|  | SCO5195 | 0.692 | 4.52E-02 | hypothetical protein |
|  | SCO1714 | 0.690 | 1.74E-02 | hypothetical protein |
|  | SCO2116 | 0.689 | 1.74E-02 | hypothetical protein |
|  | SCO4838 | 0.688 | 3.92E-03 | hypothetical protein |
|  | SCO2061 | 0.688 | 2.81E-02 | ABC transport ATP-binding subunit |
|  | SCO6451 | 0.688 | 8.15E-03 | substrate binding protein |
|  | SCO6585 | 0.680 | 4.12E-02 | succinyl-CoA synthetase subunit beta |
|  | SCO4444 | 0.680 | 1.32E-02 | glutathione peroxidase |
|  | SCO2839 | 0.680 | 2.96E-03 | lipoprotein |
|  | SCO2276 | 0.677 | 2.94E-03 | hypothetical protein |
|  | SCO1653 | 0.677 | 1.70E-02 | hypothetical protein |
|  | SCO3093 | 0.676 | 3.76E-02 | hydrolase |
|  | SCO2616 | 0.676 | 3.63E-02 | hypothetical protein |
|  | SCO5203 | 0.675 | 3.13E-02 | hypothetical protein |
|  | SCO4524 | 0.675 | 4.67E-02 | hypothetical protein |
|  | SCO1481 | 0.670 | 2.13E-02 | orotidine 5'-phosphate decarboxylase |
|  | SCO4884 | 0.670 | 7.65E-03 | lipoprotein |
|  | SCO1399 | 0.668 | 3.34E-02 | ATP/GTP-binding protein |
|  | SCO3913 | 0.667 | 4.71E-02 | hypothetical protein |
|  | SCO5150 | 0.663 | 4.26E-02 | sec-independent translocase |
|  | SCO3358 | 0.663 | 4.58E-02 | two-component system response regulator |
|  | SCO6218 | 0.662 | 7.20E-03 | phosphatase |
|  | SCO6454 | 0.661 | 1.18E-02 | ABC transporter ATP-binding protein |
|  | SCO2866 | 0.661 | 4.64E-02 | hypothetical protein |
|  | SCO0072 | 0.657 | 2.67E-03 | hypothetical protein |
|  | SCO4737 | 0.656 | 2.34E-02 | hypothetical protein |
|  | SCO5860 | 0.653 | 2.01E-02 | SuhB protein |
|  | SCO2892 | 0.648 | 1.04E-02 | hypothetical protein |
|  | SCO1776 | 0.645 | 2.17E-02 | CTP synthetase |
|  | SCO3182 | 0.644 | 4.94E-02 | UTP-glucose-1-phosphate uridylyltransferase |
|  | SCO3044 | 0.644 | 1.01E-02 | hypothetical protein |
|  | SCO2505 | 0.642 | 6.10E-03 | ABC-transporter metal-binding lipoprotein |
|  | SCO7477 | 0.642 | 1.29E-02 | hypothetical protein |
|  | SCO1780 | 0.642 | 3.19E-02 | DNA repair protein |
|  | SCO2042 | 0.641 | 4.33E-02 | hypothetical protein |
|  | SCO4223 | 0.639 | 7.07E-04 | AraC family transcription regulator |
|  | SCO5862 | 0.639 | 2.58E-02 | two-component regulator CutR |
|  | SCO4904 | 0.639 | 1.03E-02 | hypothetical protein |
|  | SCO2158 | 0.638 | 1.77E-02 | kinase |
|  | SCO3378 | 0.637 | 4.65E-02 | small membrane protein |
|  | SCO7453 | 0.637 | 7.20E-03 | hypothetical protein |
|  | SCO4263 | 0.634 | 2.75E-04 | transcriptional regulator |
|  | SCO2837 | 0.634 | 4.37E-02 | hypothetical protein |
|  | SCO7459 | 0.634 | 2.70E-02 | ABC transporter ATP-binding protein |
|  | SCO3111 | 0.632 | 2.15E-02 | ABC transporter ATP-binding protein |
|  | SCO3080 | 0.630 | 5.06E-03 | hypothetical protein |
|  | SCO5458 | 0.630 | 2.45E-02 | lipoprotein |
|  | SCO4669 | 0.628 | 2.52E-03 | hypothetical protein |
|  | SCO1482 | 0.627 | 8.35E-03 | dihydroorotate dehydrogenase 2 |
|  | SCO6773 | 0.625 | 5.39E-03 | peptidase |
|  | SCO6433 | 0.624 | 8.70E-03 | hypothetical protein |
|  | SCO6453 | 0.624 | 3.18E-02 | transport permease |
|  | SCO3359 | 0.622 | 1.86E-02 | sensory histidine kinase contains hydrophobic membrane spanning regions |
|  | SCO2085 | 0.622 | 3.05E-02 | cell division protein |
|  | SCO4947 | 0.622 | 1.22E-04 | nitrate reductase subunit alpha NarG3 |
|  | SCO1124 | 0.621 | 4.86E-02 | hypothetical protein |
|  | SCO2060 | 0.620 | 3.99E-02 | integral membrane transport protein |
|  | SCO5672 | 0.619 | 4.95E-02 | hypothetical protein |
|  | SCO4925 | 0.618 | 4.45E-02 | hypothetical protein |
|  | SCO3722 | 0.617 | 1.16E-02 | transcriptional regulator |
|  | SCO2848 | 0.615 | 1.34E-02 | hypothetical protein |
|  | SCO4599 | 0.615 | 5.01E-02 | NADH dehydrogenase subunit NuoA2 |
|  | SCO4013 | 0.612 | 4.50E-02 | penicillin-binding protein |
|  | SCO4068 | 0.609 | 4.00E-02 | phosphoribosylamine--glycine ligase |
|  | SCO7385 | 0.609 | 3.34E-02 | hypothetical protein |
|  | SCO0496 | 0.608 | 1.55E-02 | iron-siderophore permease transmembrane protein |
|  | SCO6091 | 0.608 | 2.00E-02 | hypothetical protein |
|  | SCO4909 | 0.607 | 4.39E-03 | ATP-binding protein |
|  | SCO1777 | 0.607 | 1.96E-02 | hypothetical protein |
|  | SCO0229 | 0.605 | 1.51E-02 | short chain dehydrogenase |
|  | SCO7462 | 0.603 | 1.56E-02 | hypothetical protein |
|  | SCO5155 | 0.598 | 6.50E-03 | lipoprotein |
|  | SCO2057 | 0.589 | 3.19E-02 | hypothetical protein |
|  | SCO4493 | 0.589 | 4.65E-02 | transcription regulator AsnC |
|  | SCO3612 | 0.588 | 1.49E-02 | hypothetical protein |
|  | SCO1245 | 0.587 | 3.12E-02 | adenosylmethionine--8-amino-7-oxononanoate aminotransferase BioA |
|  | SCO0688 | 0.586 | 3.49E-03 | hypothetical protein |
|  | SCO5116 | 0.586 | 3.47E-02 | peptide transport system ATP-binding subunit |
|  | SCO5144 | 0.585 | 3.31E-02 | acyl CoA isomerase |
|  | SCO6436 | 0.584 | 2.52E-02 | tRNA synthetase |
|  | SCO0918 | 0.583 | 1.45E-03 | excinuclease ABC subunit A |
|  | SCO1698 | 0.583 | 3.08E-02 | hypothetical protein |
|  | SCO3896 | 0.582 | 2.53E-02 | RNA nucleotidyltransferase |
|  | SCO4590 | 0.581 | 1.94E-02 | hypothetical protein |
|  | SCO6432 | 0.580 | 9.86E-03 | peptide synthase |
|  | SCO2287 | 0.580 | 2.34E-02 | oxidoreductase |
|  | SCO4184 | 0.580 | 2.63E-02 | aerial mycelium formation protein |
|  | SCO3577 | 0.577 | 2.73E-02 | ion-transporting ATPase |
|  | SCO1115 | 0.576 | 3.52E-02 | hypothetical protein |
|  | SCO2516 | 0.576 | 7.20E-03 | hypothetical protein |
|  | SCO4047 | 0.574 | 4.85E-02 | hypothetical protein |
|  | SCO3311 | 0.572 | 1.04E-02 | delta-aminolevulinic acid dehydratase |
|  | SCO4993 | 0.566 | 6.70E-04 | metal ion transport protein |
|  | SCO3149 | 0.559 | 2.74E-02 | dimethyladenosine transferase |
|  | SCO3046 | 0.558 | 8.34E-03 | hypothetical protein |
|  | SCO4059 | 0.557 | 2.25E-02 | transcriptional regulator |
|  | SCO1337 | 0.554 | 2.80E-02 | oxidoreductase |
|  | SCO6462 | 0.550 | 3.34E-02 | methylated-DNA-protein-cysteine methyltransferase |
|  | SCO2939 | 0.550 | 2.73E-02 | hypothetical protein |
|  | SCO6140 | 0.549 | 4.25E-02 | two-component system response regulator |
|  | SCO4171 | 0.548 | 3.39E-02 | hypothetical protein |
|  | SCO2175 | 0.538 | 2.35E-02 | nicotinate-nucleotide--dimethylbenzimidazole phosphoribosyltransferase |
|  | SCO3282 | 0.536 | 1.83E-02 | precorrin-8X methylmutase |
|  | SCO1826 | 0.534 | 4.07E-02 | hypothetical protein |
|  | SCO5138 | 0.532 | 3.19E-02 | hypothetical protein |
|  | SCO3864 | 0.532 | 3.53E-02 | transcriptional regulator |
|  | SCO2334 | 0.531 | 2.65E-02 | hypothetical protein |
|  | SCO3176 | 0.531 | 1.01E-02 | hypothetical protein |
|  | SCO4752 | 0.528 | 3.93E-02 | DNA-binding/iron metalloprotein/AP endonuclease |
|  | SCO0465 | 0.527 | 3.12E-02 | non-heme chloroperoxidase |
|  | SCO3703 | 0.526 | 2.45E-02 | substrate binding protein |
|  | SCO4747 | 0.526 | 3.12E-02 | ATP/GTP binding protein |
|  | SCO1101 | 0.525 | 2.90E-02 | hypothetical protein |
|  | SCO3868 | 0.519 | 3.96E-02 | hypothetical protein |
|  | SCO6687 | 0.518 | 6.79E-03 | DNA-binding protein |
|  | SCO4740 | 0.511 | 1.57E-02 | glucosamine--fructose-6-phosphate aminotransferase |
|  | SCO2897 | 0.505 | 3.41E-02 | penicillin-binding protein |
|  | SCO4850 | 0.501 | 2.27E-02 | TetR family transcriptional regulator |
| downregulated | SCO0171 | -0.504 | 2.33E-02 | nicotinate phosphoribosyltransferase |
|  | SCO5005 | -0.505 | 4.39E-02 | hypothetical protein |
|  | SCO4290 | -0.505 | 7.55E-03 | trehalose-phosphate synthase |
|  | SCO3656 | -0.509 | 8.14E-03 | hypothetical protein |
|  | SCO5442 | -0.517 | 6.59E-03 | trehalose synthase |
|  | SCO1369 | -0.521 | 2.56E-02 | two component system histidine kinase |
|  | SCO1568 | -0.521 | 3.65E-02 | TetR family transcriptional regulator |
|  | SCO6110 | -0.522 | 2.99E-02 | sugar kinase |
|  | SCO4571 | -0.523 | 2.53E-02 | NADH dehydrogenase subunit J |
|  | SCO6029 | -0.525 | 4.52E-02 | two-component regulator |
|  | SCO6359 | -0.525 | 2.32E-02 | hypothetical protein |
|  | SCO4976 | -0.530 | 4.98E-03 | hypothetical protein |
|  | SCO2883 | -0.531 | 4.61E-02 | cytochrome P450 |
|  | SCO1198 | -0.535 | 3.38E-02 | acyl-CoA dehydrogenase |
|  | SCO5028 | -0.535 | 2.31E-03 | ATP-binding protein |
|  | SCO7519 | -0.542 | 2.27E-02 | sugar acetyltransferase |
|  | SCO1889 | -0.545 | 1.79E-02 | dihydropicolinate synthase |
|  | SCO3007 | -0.548 | 3.52E-03 | hypothetical protein |
|  | SCO5629 | -0.548 | 4.33E-04 | ATP /GTP-binding protein |
|  | SCO1817 | -0.550 | 1.47E-02 | hypothetical protein |
|  | SCO1085 | -0.550 | 3.39E-02 | acyltransferase |
|  | SCO5929 | -0.552 | 2.36E-02 | oxidoreductase |
|  | SCO0985 | -0.553 | 5.60E-03 | 5-methyltetrahydropteroyltriglutamate/homocysteine S-methyltransferase |
|  | SCO3104 | -0.554 | 2.10E-04 | hypothetical protein |
|  | SCO4630 | -0.554 | 1.65E-02 | hypothetical protein |
|  | SCO0972 | -0.558 | 2.94E-02 | transposase remnant |
|  | SCO2827 | -0.559 | 8.81E-04 | hypothetical protein |
|  | SCO4237 | -0.559 | 3.50E-02 | hypothetical protein |
|  | SCO6394 | -0.559 | 9.15E-03 | IS element ATP binding protein |
|  | SCO0198 | -0.559 | 4.66E-02 | hypothetical protein |
|  | SCO1981 | -0.560 | 2.24E-02 | hypothetical protein |
|  | SCO5975 | -0.561 | 1.01E-02 | arginine deiminase |
|  | SCO7367 | -0.562 | 3.52E-03 | membrane efflux protein |
|  | SCO6504 | -0.563 | 5.85E-03 | hypothetical protein |
|  | SCO7842 | -0.564 | 3.65E-03 | transposase |
|  | SCO3383 | -0.570 | 1.83E-02 | pantoate--beta-alanine ligase |
|  | SCO3119 | -0.571 | 1.14E-02 | hypothetical protein |
|  | SCO0073 | -0.571 | 3.48E-02 | hypothetical protein |
|  | SCO4565 | -0.573 | 3.04E-03 | NADH dehydrogenase subunit D |
|  | SCO0005 | -0.573 | 2.86E-03 | transposase |
|  | SCO0617 | -0.574 | 8.86E-03 | phosphoketolase |
|  | SCO3350 | -0.574 | 3.51E-02 | hypothetical protein |
|  | SCO2524 | -0.575 | 3.85E-02 | hypothetical protein |
|  | SCO5801 | -0.575 | 2.88E-02 | hypothetical protein |
|  | SCO1630 | -0.575 | 1.92E-02 | hypothetical protein |
|  | SCO0149 | -0.578 | 3.83E-02 | hypothetical protein |
|  | SCO0203 | -0.580 | 1.51E-02 | two-component sensor |
|  | SCO6809 | -0.581 | 4.39E-02 | integral membrane transport protein |
|  | SCO2529 | -0.582 | 1.52E-03 | metalloprotease |
|  | SCO1648 | -0.582 | 3.09E-02 | ATPase AAA |
|  | SCO0428 | -0.583 | 4.31E-02 | tetR family transcriptional regulator |
|  | SCO2924 | -0.585 | 2.73E-02 | regulator |
|  | SCO6777 | -0.586 | 7.62E-03 | beta-lactamase |
|  | SCO6808 | -0.586 | 1.79E-02 | transcription regulator ArsR |
|  | SCO6211 | -0.587 | 6.48E-03 | uricase |
|  | SCO4609 | -0.588 | 3.18E-02 | heat shock protein HtpX |
|  | SCO0588 | -0.591 | 7.65E-03 | sensor kinase |
|  | SCO6519 | -0.593 | 1.45E-02 | lyase |
|  | SCO1489 | -0.596 | 4.75E-02 | DNA-binding protein |
|  | SCO6305 | -0.601 | 7.20E-03 | hypothetical protein |
|  | SCO0208 | -0.602 | 3.60E-04 | pyruvate phosphate dikinase |
|  | SCO0855 | -0.603 | 4.16E-02 | acetyltransferase |
|  | SCP2.02 | -0.604 | 1.46E-03 | putative is1648 transposase |
|  | SCO1627 | -0.607 | 3.13E-02 | ATP-GTP binding protein |
|  | SCO5578 | -0.608 | 2.24E-02 | sugar transporter |
|  | SCO4006 | -0.609 | 1.77E-04 | long-chain-fatty-acid--CoA ligase |
|  | SCO0210 | -0.614 | 4.68E-02 | hypothetical protein |
|  | SCO5221 | -0.615 | 7.54E-04 | polypeptide deformylase |
|  | SCO7011 | -0.617 | 1.83E-02 | membrane transport protein |
|  | SCO0166 | -0.617 | 1.10E-02 | regulator |
|  | SCO1143 | -0.617 | 4.93E-02 | hypothetical protein |
|  | SCO1726 | -0.619 | 1.18E-03 | ATPase |
|  | SCO4784 | -0.621 | 3.19E-02 | hypothetical protein |
|  | SCO1796 | -0.623 | 1.56E-02 | hypothetical protein |
|  | SCO5024 | -0.623 | 2.20E-02 | oxidoreductase |
|  | SCO2655 | -0.625 | 2.80E-02 | hypothetical protein |
|  | SCO1404 | -0.630 | 1.39E-02 | hypothetical protein |
|  | SCO0784 | -0.631 | 3.09E-02 | hypothetical protein |
|  | SCO2108 | -0.633 | 3.41E-02 | hypothetical protein |
|  | SCO4279 | -0.634 | 1.50E-02 | acetyltransferase |
|  | SCO7153 | -0.635 | 1.03E-02 | sugar transporter |
|  | SCO0907 | -0.638 | 6.48E-03 | dehydrogenase |
|  | SCO4092 | -0.641 | 7.67E-03 | ATP-dependent helicase |
|  | SCO6354 | -0.642 | 2.01E-03 | two-component regulator |
|  | SCO2649 | -0.642 | 1.16E-05 | 4-alpha-glucanotransferase |
|  | SCO4131 | -0.643 | 4.80E-04 | hypothetical protein |
|  | SCO7722 | -0.645 | 7.65E-03 | hypothetical protein |
|  | SCO6464 | -0.646 | 1.48E-02 | SIR2 family transcriptional regulator |
|  | SCO5506 | -0.647 | 2.72E-06 | regulatory protein |
|  | SCO1322 | -0.652 | 3.39E-03 | hypothetical protein |
|  | SCO5700 | -0.653 | 4.83E-03 | kinase |
|  | SCO6055 | -0.654 | 2.27E-02 | carbonic anhydrase |
|  | SCO6094 | -0.655 | 4.65E-02 | transport system integral membrane protein |
|  | SCO2520 | -0.657 | 1.90E-03 | hypothetical protein |
|  | SCO4690 | -0.657 | 3.34E-03 | hypothetical protein |
|  | SCO3206 | -0.657 | 3.58E-03 | transmembrane efflux protein |
|  | SCO5222 | -0.657 | 6.41E-04 | lyase |
|  | SCO4610 | -0.658 | 4.14E-05 | hypothetical protein |
|  | SCO4689 | -0.660 | 3.81E-03 | hypothetical protein |
|  | SCO6811 | -0.661 | 3.33E-02 | hypothetical protein |
|  | SCO1657 | -0.662 | 3.47E-02 | methionine synthase |
|  | SCO2697 | -0.664 | 4.37E-02 | hypothetical protein |
|  | SCO2465 | -0.666 | 8.52E-03 | RNA polymerase principal sigma factor |
|  | SCO1108 | -0.667 | 1.03E-02 | regulatory protein |
|  | SCO5511 | -0.669 | 2.97E-02 | membrane associated phophodiesterase |
|  | SCO4679 | -0.669 | 5.13E-03 | hypothetical protein |
|  | SCO0181 | -0.672 | 9.05E-04 | hypothetical protein |
|  | SCO4207 | -0.672 | 3.45E-02 | hypothetical protein |
|  | SCO3284 | -0.673 | 1.82E-05 | hypothetical protein |
|  | SCO0220 | -0.675 | 1.55E-02 | hypothetical protein |
|  | SCO2449 | -0.677 | 3.55E-03 | hypothetical protein |
|  | SCO4594 | -0.679 | 8.02E-04 | 2-oxoglutarate ferredoxin oxidoreductase subunit beta |
|  | SCO4676 | -0.681 | 4.27E-03 | hypothetical protein |
|  | SCO0177 | -0.682 | 3.34E-03 | hypothetical protein |
|  | SCO3928 | -0.683 | 2.93E-02 | thiamine biosynthesis protein ThiC |
|  | SCO3826 | -0.687 | 1.06E-04 | ion channel membrane protein |
|  | SCO0219 | -0.688 | 9.15E-03 | nitrate reductase subunit delta NarI2 |
|  | SCO2372 | -0.690 | 4.71E-02 | small hydrophobic protein |
|  | SCO0906 | -0.690 | 2.70E-02 | hypothetical protein |
|  | SCO0834 | -0.690 | 3.06E-03 | hypothetical protein |
|  | SCO4563 | -0.691 | 1.70E-02 | NADH dehydrogenase subunit B |
|  | SCO4308 | -0.692 | 7.54E-04 | transcriptional regulator |
|  | SCO6228 | -0.694 | 8.08E-03 | hypothetical protein |
|  | SCO5250 | -0.697 | 9.84E-04 | polyprenyl synthetase |
|  | SCO6981 | -0.698 | 1.57E-03 | ABC transporter ATP-binding protein |
|  | SCO4245 | -0.703 | 2.09E-05 | hypothetical protein |
|  | SCO2920 | -0.706 | 1.34E-02 | protease |
|  | SCO6805 | -0.708 | 2.29E-04 | integral membrane efflux protein |
|  | SCO1963 | -0.708 | 6.08E-03 | integral membrane export protein |
|  | SCO1647 | -0.711 | 2.31E-03 | hypothetical protein |
|  | SCO2877 | -0.711 | 1.90E-02 | hypothetical protein |
|  | SCO4222 | -0.712 | 1.52E-03 | hypothetical protein |
|  | SCO1927 | -0.712 | 8.97E-04 | AAC(3) family N-acetyltransferase |
|  | SCO5252 | -0.713 | 2.39E-02 | hypothetical protein |
|  | SCO0215 | -0.714 | 6.68E-05 | hypothetical protein |
|  | SCO7205 | -0.717 | 1.62E-03 | hydrolase |
|  | SCO3340 | -0.719 | 1.72E-03 | hypothetical protein |
|  | SCO7366 | -0.720 | 2.17E-02 | hypothetical protein |
|  | SCO7016 | -0.722 | 3.06E-04 | LacI family transcriptional regulator |
|  | SCO7700 | -0.722 | 9.32E-03 | cyclase |
|  | SCO5207 | -0.727 | 9.10E-03 | hypothetical protein |
|  | SCO5166 | -0.729 | 9.13E-03 | helicase |
|  | SCO6108 | -0.730 | 1.24E-02 | esterase |
|  | SCO1812 | -0.730 | 4.06E-05 | integral membrane transporter |
|  | SCO2383 | -0.730 | 2.85E-03 | hypothetical protein |
|  | SCO0921 | -0.732 | 1.54E-03 | hypothetical protein |
|  | SCO1406 | -0.732 | 3.04E-03 | hypothetical protein |
|  | SCO6353 | -0.734 | 4.83E-05 | two-component sensor |
|  | SCO3165 | -0.735 | 2.36E-03 | hypothetical protein |
|  | SCO6269 | -0.735 | 5.43E-05 | 2-oxoglutarate ferredoxin oxidoreductase subunit beta |
|  | SCO4564 | -0.736 | 1.90E-03 | NADH dehydrogenase subunit C |
|  | SCO7468 | -0.737 | 9.55E-04 | flavin-binding monooxygenase |
|  | SCO6096 | -0.742 | 2.72E-02 | lipoprotein |
|  | SCO1903 | -0.743 | 3.34E-03 | transport associated protein |
|  | SCO3765 | -0.748 | 1.06E-03 | hypothetical protein |
|  | SCO6222 | -0.755 | 1.15E-04 | aminotransferase AlaT |
|  | SCO1134 | -0.757 | 1.92E-03 | oxidoreductase%2C iron-sulfur binding subunit |
|  | SCO5174 | -0.758 | 3.60E-04 | transferase |
|  | SCO7609 | -0.769 | 2.59E-03 | esterase |
|  | SCO4070 | -0.772 | 6.86E-03 | hypothetical protein |
|  | SCO6663 | -0.774 | 5.21E-04 | transketolase |
|  | SCO3952 | -0.776 | 2.86E-03 | hypothetical protein |
|  | SCO5025 | -0.776 | 3.78E-04 | transcriptional regulator |
|  | SCO7651 | -0.777 | 9.79E-04 | TetR family transcriptional regulator |
|  | SCO2496 | -0.780 | 3.10E-03 | hypothetical protein |
|  | SCO1132 | -0.784 | 6.32E-07 | oxidoreductase |
|  | SCO5811 | -0.785 | 4.28E-05 | transcriptional regulator |
|  | SCO4347 | -0.788 | 6.59E-07 | hypothetical protein |
|  | SCO5010 | -0.790 | 1.68E-05 | hypothetical protein |
|  | SCO3607 | -0.792 | 8.96E-03 | hypothetical protein |
|  | SCO5107 | -0.794 | 1.56E-03 | succinate dehydrogenase flavoprotein subunit |
|  | SCO6952 | -0.794 | 7.86E-04 | hypothetical protein |
|  | SCO4188 | -0.797 | 3.21E-06 | GntR family transcriptional regulator |
|  | SCO5175 | -0.798 | 2.64E-05 | hypothetical protein |
|  | SCO0174 | -0.804 | 1.72E-04 | DNA-binding protein |
|  | SCO1968 | -0.805 | 1.05E-03 | hydrolase |
|  | SCO6513 | -0.806 | 2.76E-04 | hypothetical protein |
|  | SCO7434 | -0.807 | 7.53E-04 | lipoprotein |
|  | SCO2464 | -0.811 | 3.50E-07 | ABC transporter |
|  | SCO1567 | -0.812 | 1.06E-05 | transmembrane-transport protein |
|  | SCO0200 | -0.816 | 6.44E-03 | hypothetical protein |
|  | SCO0585 | -0.819 | 2.75E-04 | ATP/GTP binding protein |
|  | SCO6073 | -0.827 | 5.04E-05 | cyclase |
|  | SCO3035 | -0.828 | 1.29E-03 | hypothetical protein |
|  | SCO3366 | -0.828 | 2.67E-03 | exporter |
|  | SCO0204 | -0.829 | 4.75E-03 | LuxR family transcriptional regulator |
|  | SCO1799 | -0.829 | 1.27E-05 | hypothetical protein |
|  | SCO5004 | -0.831 | 2.02E-04 | hypothetical protein |
|  | SCO7841 | -0.832 | 3.00E-04 | ATP/GTP-binding protein |
|  | SCO1474 | -0.839 | 1.21E-03 | hypothetical protein |
|  | SCO2466 | -0.839 | 1.02E-02 | hypothetical protein |
|  | SCO0622 | -0.839 | 1.14E-03 | TetR family transcriptional regulator |
|  | SCO3360 | -0.840 | 9.42E-06 | hypothetical protein |
|  | SCO2625 | -0.840 | 3.07E-06 | hypothetical protein |
|  | SCO1867 | -0.847 | 2.84E-03 | hydroxylase |
|  | SCO5015 | -0.853 | 7.55E-05 | hypothetical protein |
|  | SCO0685 | -0.853 | 1.89E-03 | hypothetical protein |
|  | SCO3764 | -0.856 | 2.12E-08 | hypothetical protein |
|  | SCO0217 | -0.858 | 6.76E-05 | nitrate reductase subunit beta NarH2 |
|  | SCO5473 | -0.860 | 8.96E-04 | ATP/GTP binding protein |
|  | SCO7652 | -0.861 | 6.08E-03 | acetyltransferase |
|  | SCO1624 | -0.866 | 6.44E-04 | acetyltransferase |
|  | SCO0993 | -0.866 | 8.19E-06 | hypothetical protein |
|  | SCO0006 | -0.867 | 1.14E-04 | ATP/GTP-binding protein |
|  | SCO6304 | -0.868 | 4.26E-05 | oxidoreductase |
|  | SCO2765 | -0.868 | 5.72E-05 | hypothetical protein |
|  | SCO1457 | -0.869 | 2.14E-04 | transporter |
|  | SCO3606 | -0.869 | 9.44E-04 | regulator |
|  | SCO0525 | -0.873 | 1.75E-03 | hypothetical protein |
|  | SCO3601 | -0.875 | 1.50E-05 | hypothetical protein |
|  | SCO6099 | -0.878 | 2.46E-03 | adenylyl-sulfate kinase |
|  | SCO1987 | -0.879 | 4.35E-03 | hypothetical protein |
|  | SCO7447 | -0.884 | 5.07E-04 | acetyltranferase |
|  | SCO1866 | -0.885 | 2.40E-03 | L-ectoine synthase |
|  | SCO1988 | -0.890 | 2.14E-04 | hypothetical protein |
|  | SCO0199 | -0.890 | 2.04E-04 | alcohol dehydrogenase |
|  | SCO1307 | -0.893 | 1.59E-04 | hypothetical protein |
|  | SCO5448 | -0.903 | 1.61E-02 | ABC transporter |
|  | SCO2343 | -0.903 | 7.47E-05 | acetyltransferase |
|  | SCO2016 | -0.904 | 8.15E-07 | monooxygenase |
|  | SCO1816 | -0.906 | 8.28E-07 | hypothetical protein |
|  | SCO4164 | -0.914 | 8.10E-04 | thiosulfate sulfurtransferase |
|  | SCO4311 | -0.917 | 2.75E-06 | hypothetical protein |
|  | SCO3323 | -0.919 | 1.35E-05 | RNA polymerase sigma factor |
|  | SCO2113 | -0.921 | 4.98E-03 | bacterioferritin |
|  | SCO2911 | -0.922 | 1.62E-05 | hypothetical protein |
|  | SCO4337 | -0.924 | 5.55E-05 | integral membrane efflux protein |
|  | SCO2236 | -0.925 | 1.60E-04 | hypothetical protein |
|  | SCO0924 | -0.927 | 7.62E-03 | cytochrome B subunit |
|  | SCO5861 | -0.927 | 5.57E-05 | hypothetical protein |
|  | SCO2117 | -0.929 | 8.24E-08 | anthranilate synthase |
|  | SCO3296 | -0.931 | 3.52E-03 | oxidoreductase |
|  | SCO7014 | -0.933 | 4.13E-06 | LacI family transcriptional regulator |
|  | SCO1144 | -0.934 | 5.70E-08 | ABC transporter ATP-binding protein |
|  | SCO7709 | -0.937 | 8.37E-06 | MarR family transcriptional regulator |
|  | SCO2248 | -0.939 | 2.61E-05 | hypothetical protein |
|  | SCO6102 | -0.939 | 2.96E-03 | nitrite/sulfite reductase |
|  | SCO1569 | -0.949 | 1.05E-03 | oxidoreductase |
|  | SCO3277 | -0.950 | 3.33E-04 | phosphotransferase |
|  | SCO5006 | -0.952 | 1.37E-07 | septum site-determining protein |
|  | SCO4468 | -0.957 | 9.92E-04 | hypothetical protein |
|  | SCO6097 | -0.962 | 4.68E-04 | sulfate adenylyltransferase subunit 1 |
|  | SCO1083 | -0.968 | 1.34E-04 | flavin-dependent reductase |
|  | SCO0922 | -0.973 | 1.82E-04 | succinate dehydrogenase/fumarate reductase iron-sulfur subunit |
|  | SCO0991 | -0.977 | 3.55E-04 | hypothetical protein |
|  | SCO5177 | -0.979 | 2.88E-05 | hypothetical protein |
|  | SCO1405 | -0.986 | 2.52E-07 | HSP90 family protein |
|  | SCO2463 | -0.987 | 5.44E-05 | ABC transporter |
|  | SCO5949 | -0.987 | 1.78E-05 | hypothetical protein |
|  | SCO1545 | -0.987 | 2.75E-04 | acetyltransferase |
|  | SCO1731 | -0.990 | 2.09E-07 | hypothetical protein |
|  | SCO7265 | -1.002 | 6.31E-05 | hypothetical protein |
|  | SCO5774 | -1.004 | 2.55E-06 | glutamate permease |
|  | SCO1673 | -1.007 | 2.10E-04 | hypothetical protein |
|  | SCO0908 | -1.008 | 7.81E-06 | hypothetical protein |
|  | SCO0216 | -1.020 | 3.79E-07 | nitrate reductase subunit alpha NarG2 |
|  | SCO0213 | -1.021 | 3.59E-07 | nitrate/nitrite transporter |
|  | SCO2311 | -1.024 | 2.74E-09 | hypothetical protein |
|  | SCO6446 | -1.032 | 1.26E-08 | hypothetical protein |
|  | SCO0587 | -1.033 | 2.27E-05 | hypothetical protein |
|  | SCO2876 | -1.038 | 6.98E-08 | acetyltransferase |
|  | SCO7507 | -1.039 | 1.16E-06 | dioxygenase |
|  | SCO6098 | -1.039 | 1.03E-04 | sulfate adenylyltransferase subunit 2 |
|  | SCO7653 | -1.045 | 1.05E-03 | hypothetical protein |
|  | SCO7197 | -1.048 | 5.21E-10 | amino acid ABC transporter permease |
|  | SCO1586 | -1.056 | 2.61E-06 | hypothetical protein |
|  | SCO1133 | -1.059 | 1.75E-08 | oxidoreductase%2C molybdopterin binding subunit |
|  | SCO6517 | -1.064 | 3.56E-07 | uvrA-like protein |
|  | SCO5249 | -1.068 | 3.11E-07 | nucleotide-binding protein |
|  | SCO1341 | -1.070 | 2.23E-06 | lipoprotein |
|  | SCO1148 | -1.076 | 7.34E-13 | ABC transporter |
|  | SCO3132 | -1.078 | 1.35E-05 | trans-aconitate 2-methyltransferase |
|  | SCO3788 | -1.083 | 5.50E-04 | hypothetical protein |
|  | SCO6797 | -1.086 | 9.37E-07 | ATP/GTP binding protein |
|  | SCO3917 | -1.090 | 3.00E-08 | hypothetical protein |
|  | SCO2027 | -1.091 | 5.54E-09 | hypothetical protein |
|  | SCO6100 | -1.096 | 1.15E-04 | phosphoadenosine phosphosulfate reductase |
|  | SCO0995 | -1.100 | 3.00E-04 | methyltransferase |
|  | SCO3631 | -1.101 | 3.62E-06 | hypothetical protein |
|  | SCO2591 | -1.103 | 1.77E-04 | hypothetical protein |
|  | SCO0186 | -1.113 | 3.94E-04 | phytoene dehydrogenase |
|  | SCO4964 | -1.121 | 1.68E-10 | integral membrane transport protein |
|  | SCO6149 | -1.123 | 2.74E-09 | ribosome-associated GTPase |
|  | SCO5009 | -1.124 | 2.63E-10 | secretory protein |
|  | SCO0185 | -1.126 | 4.70E-03 | geranylgeranyl pyrophosphate synthase |
|  | SCO3562 | -1.135 | 2.41E-16 | integral membrane transport protein |
|  | SCO2912 | -1.144 | 2.23E-05 | hypothetical protein |
|  | SCO0218 | -1.148 | 1.96E-07 | nitrate reductase subunit delta NarJ2 |
|  | SCO1585 | -1.152 | 5.25E-05 | hypothetical protein |
|  | SCO0796 | -1.154 | 8.76E-10 | hypothetical protein |
|  | SCO0190 | -1.161 | 4.90E-06 | methyltransferase |
|  | SCO1557 | -1.168 | 2.88E-05 | lipoprotein |
|  | SCO5176 | -1.171 | 2.17E-08 | reductase |
|  | SCO3608 | -1.187 | 4.11E-04 | hypothetical protein |
|  | SCO0586 | -1.197 | 1.47E-07 | hypothetical protein |
|  | SCO2527 | -1.201 | 3.82E-11 | hypothetical protein |
|  | SCO4963 | -1.204 | 9.92E-08 | ABC transporter ATP-binding protein |
|  | SCO1729 | -1.209 | 5.73E-10 | hypothetical protein |
|  | SCO2379 | -1.221 | 6.36E-09 | acetyltransferase |
|  | SCO7710 | -1.229 | 5.55E-11 | phosphotransferase |
|  | SCO0187 | -1.239 | 2.75E-04 | phytoene synthase |
|  | SCO4007 | -1.256 | 1.81E-16 | hypothetical protein |
|  | SCO1576 | -1.270 | 3.09E-05 | arginine repressor |
|  | SCO3287 | -1.288 | 4.37E-04 | serine/arginine rich protein |
|  | SCO2755 | -1.291 | 3.77E-09 | acetyltransferase |
|  | SCO2213 | -1.294 | 7.51E-07 | regulatory protein |
|  | SCO0994 | -1.308 | 4.72E-15 | hypothetical protein |
|  | SCO5449 | -1.316 | 2.52E-14 | ABC transporter |
|  | SCO5190 | -1.323 | 2.64E-12 | DNA-binding protein |
|  | SCO4055 | -1.325 | 2.33E-17 | alcohol dehydrogenase |
|  | SCO5974 | -1.329 | 6.20E-05 | integral membrane ion exchanger |
|  | SCO1986 | -1.331 | 2.96E-06 | hypothetical protein |
|  | SCO3602 | -1.335 | 4.75E-18 | transmembrane transport protein |
|  | SCO3006 | -1.336 | 9.49E-16 | acetyltransferase |
|  | SCO5450 | -1.356 | 2.37E-22 | ABC transporter |
|  | SCO1577 | -1.356 | 2.03E-06 | acetylornithine aminotransferase |
|  | SCO7606 | -1.356 | 8.83E-11 | amino acid binding protein |
|  | SCO6512 | -1.357 | 8.83E-13 | ABC transporter ATP-binding protein |
|  | SCO4765 | -1.362 | 2.10E-13 | hypothetical protein |
|  | SCO2373 | -1.393 | 5.43E-08 | tetracenomycin C efflux protein |
|  | SCO6056 | -1.396 | 2.29E-12 | hypothetical protein |
|  | SCO7731 | -1.409 | 1.54E-13 | hypothetical protein |
|  | SCO0593 | -1.416 | 1.55E-12 | hypothetical protein |
|  | SCO4278 | -1.435 | 1.02E-12 | peptidyl-tRNA hydrolase domain-containing protein |
|  | SCO5772 | -1.445 | 1.84E-09 | hypothetical protein |
|  | SCO6295 | -1.445 | 1.73E-21 | ABC transporter ATP-binding protein |
|  | SCO6447 | -1.451 | 4.48E-11 | NAD(P)H oxidoreductase |
|  | SCO7036 | -1.452 | 1.12E-07 | argininosuccinate synthase |
|  | SCO5191 | -1.453 | 3.68E-10 | hypothetical protein |
|  | SCO4264 | -1.463 | 1.18E-23 | aminoglycoside phosphotransferase |
|  | SCO2247 | -1.473 | 2.52E-13 | hypothetical protein |
|  | SCO0909 | -1.483 | 1.18E-09 | hypothetical protein |
|  | SCO5189 | -1.514 | 1.52E-08 | hypothetical protein |
|  | SCO1084 | -1.516 | 2.93E-12 | thioredoxin |
|  | SCO3286 | -1.531 | 1.04E-14 | hypothetical protein |
|  | SCO7478 | -1.553 | 2.58E-13 | phosphotransferase |
|  | SCO4054 | -1.554 | 9.92E-08 | hypothetical protein |
|  | SCO3710 | -1.557 | 1.93E-11 | large integral membrane protein |
|  | SCO1147 | -1.573 | 1.64E-27 | ABC transporter transmembrane subunit |
|  | SCO0408 | -1.575 | 2.56E-13 | methyltransferase |
|  | SCO1570 | -1.583 | 2.57E-11 | argininosuccinate lyase |
|  | SCO7806 | -1.595 | 9.16E-10 | DNA-binding protein |
|  | SCO4293 | -1.596 | 4.54E-16 | threonine synthase |
|  | SCO7721 | -1.624 | 1.01E-24 | hypothetical protein |
|  | SCO1559 | -1.654 | 2.30E-09 | ABC transporter ATP-binding protein |
|  | SCO5796 | -1.676 | 2.12E-10 | hypothetical protein |
|  | SCO1578 | -1.680 | 2.71E-10 | acetylglutamate kinase |
|  | SCO0992 | -1.682 | 4.84E-23 | cysteine synthase |
|  | SCO3953 | -1.712 | 2.19E-26 | RNA 2'-phosphotransferase-like protein |
|  | SCO1340 | -1.724 | 1.43E-14 | hypothetical protein |
|  | SCO5977 | -1.766 | 4.70E-37 | amino acid permease |
|  | SCO5972 | -1.815 | 1.25E-27 | 3' terminal RNA ribose 2'-O-methyltransferase Hen1 |
|  | SCO0106 | -1.851 | 8.92E-14 | insertion element transposase |
|  | SCO5976 | -1.870 | 9.23E-20 | ornithine carbamoyltransferase |
|  | SCO0854 | -1.877 | 3.59E-22 | hypothetical protein |
|  | SCO2145 | -1.915 | 5.93E-16 | glycerate kinase |
|  | SCO1575 | -1.952 | 3.70E-29 | thiamine biosynthesis lipoprotein |
|  | SCO1579 | -1.964 | 5.31E-20 | bifunctional ornithine acetyltransferase/N-acetylglutamate synthase |
|  | SCO3711 | -1.967 | 1.88E-20 | small membrane protein |
|  | SCO5973 | -1.977 | 7.81E-36 | phosphatase |
|  | SCO1558 | -1.993 | 2.38E-27 | ABC transporter permease |
|  | SCO1580 | -1.995 | 1.37E-15 | N-acetyl-gamma-glutamyl-phosphate reductase |
|  | SCO0795 | -1.999 | 1.27E-28 | hypothetical protein |
|  | SCO5451 | -2.054 | 4.52E-32 | ABC transporter |
|  | SCO6530 | -2.086 | 7.64E-28 | hypothetical protein |
|  | SCO1413 | -2.094 | 2.11E-33 | hypothetical protein |
|  | SCO3824 | -2.164 | 4.35E-36 | ABC transporter ATP-binding protein |
|  | SCO3825 | -2.225 | 7.30E-38 | ABC-transporter transmembrane protein |
|  | SCO3299 | -2.243 | 9.96E-30 | hypothetical protein |
|  | SCO3285 | -2.326 | 1.42E-56 | large glycine/alanine rich protein |
|  | SCO1905 | -2.326 | 6.71E-52 | hypothetical protein |
|  | SCO2146 | -2.389 | 2.53E-30 | aminotransferase |
|  | SCO0783 | -2.467 | 1.64E-46 | tetracycline resistance protein |
|  | SCO6529 | -2.576 | 2.74E-37 | ATP/GTP binding protein |
|  | SCO1086 | -2.794 | 2.97E-30 | hypothetical protein |
